# A *Mesp1*-dependent developmental breakpoint in transcriptional and epigenomic specification of early cardiac precursors

**DOI:** 10.1242/dev.201229

**Published:** 2023-05-02

**Authors:** Alexis Leigh Krup, Sarah A. B. Winchester, Sanjeev S. Ranade, Ayushi Agrawal, W. Patrick Devine, Tanvi Sinha, Krishna Choudhary, Martin H. Dominguez, Reuben Thomas, Brian L. Black, Deepak Srivastava, Benoit G. Bruneau

**Affiliations:** ^1^ Biomedical Sciences Program, University of California, San Francisco, CA 94158, USA; ^2^ Gladstone Institutes of Cardiovascular Disease, Gladstone Institutes, San Francisco, CA 94158, USA; ^3^ Department of Pathology, University of California, San Francisco, CA 94158, USA; ^4^ Cardiovascular Research Institute, University of California, San Francisco, CA 94158, USA; ^5^ Department of Medicine, Division of Cardiology, University of California, San Francisco, CA 94158, USA; ^6^ Cardiovascular Institute and Department of Medicine, University of Pennsylvania, Philadelphia, PA 19104, USA; ^7^ Department of Biochemistry and Biophysics, University of California, San Francisco, CA 94158, USA; ^8^ Department of Pediatrics, University of California, San Francisco, CA 94158, USA; ^9^ Roddenberry Center for Stem Cell Biology and Medicine, Gladstone Institutes, San Francisco, CA 94158, USA; ^10^ Institute of Human Genetics, University of California, San Francisco, CA 94158, USA; ^11^ Eli and Edythe Broad Center of Regeneration Medicine and Stem Cell Research, University of California, San Francisco, CA 94158, USA

**Keywords:** Cardiac development, Cardiac specification, Gastrulation, Gene regulation, Mouse embryo

## Abstract

Transcriptional networks governing cardiac precursor cell (CPC) specification are incompletely understood owing, in part, to limitations in distinguishing CPCs from non-cardiac mesoderm in early gastrulation. We leveraged detection of early cardiac lineage transgenes within a granular single-cell transcriptomic time course of mouse embryos to identify emerging CPCs and describe their transcriptional profiles. *Mesp1*, a transiently expressed mesodermal transcription factor, is canonically described as an early regulator of cardiac specification. However, we observed perdurance of CPC transgene-expressing cells in *Mesp1* mutants, albeit mislocalized, prompting us to investigate the scope of the role of *Mesp1* in CPC emergence and differentiation. *Mesp1* mutant CPCs failed to robustly activate markers of cardiomyocyte maturity and crucial cardiac transcription factors, yet they exhibited transcriptional profiles resembling cardiac mesoderm progressing towards cardiomyocyte fates. Single-cell chromatin accessibility analysis defined a *Mesp1*-dependent developmental breakpoint in cardiac lineage progression at a shift from mesendoderm transcriptional networks to those necessary for cardiac patterning and morphogenesis. These results reveal *Mesp1*-independent aspects of early CPC specification and underscore a *Mesp1-*dependent regulatory landscape required for progression through cardiogenesis.

## Teaser

**Summary:** *In vivo* transcriptomic and epigenomic profiling reveals Mesp1-independent aspects of early cardiac specification and illustrates a Mesp1-dependent regulatory landscape required for progression through cardiogenesis.

## INTRODUCTION

Cardiogenesis requires precise specification and patterning of the cardiac precursor cells (CPCs) as they emerge from the gastrulating mesoderm in early embryogenesis. Errors in this process lead to congenital heart defects (CHDs), affecting 1-2% of live births worldwide (Bruneau, 2008). The genetic etiology of CHDs indicates a causative role for transcription factor haploinsufficiency, suggesting that fine dysregulation of gene expression is a crucial mechanism for disease (Zug, 2022; Nees and Chung, 2019). A thorough delineation of the transcriptional networks governing cardiogenesis is foundational to understanding how defects in this process manifest as CHDs and may inform the design of strategies to treat CHDs and heart disease broadly.

Distinguishing the earliest cardiac cells from surrounding developing mesoderm has historically been challenging owing to a paucity of CPC-specific molecular markers. Prior studies used lineage tracing of mesoderm progenitors expressing the basic helix-loop-helix (bHLH) transcription factor (TF) *Mesp1*, which is transiently expressed in cells that go on to contribute to the heart, somitic mesoderm derivatives, and craniofacial mesoderm (Devine et al., 2014; Lescroart et al., 2014; Saga et al., 1999). Such studies show that a subset of *Mesp1*^+^ cells at early gastrulation are fated for distinct cardiac substructures well before organ establishment (Devine et al., 2014; Lescroart et al., 2014; Liu, 2017; Zhang et al., 2021).

Deletion of *Mesp1* in mice variably disrupts cardiac progenitor specification and migration (Ajima et al., 2021; Saga et al., 2000, 1999; Kitajima et al., 2000; Lescroart et al., 2018). *In vitro* overexpression of *Mesp1* induces expression of cardiac TFs, indicating a potentially instructive role in cardiogenesis (Chiapparo et al., 2016; Bondue et al., 2008; Lindsley et al., 2008; Soibam et al., 2015). Gain-of-function experiments suggest a broad and important function for *Mesp1* in mesoderm differentiation, but how the *in vivo* regulatory landscape is controlled by *Mesp1* remains unclear (Costello et al., 2011; Saga et al., 1999, 2000, 1996; Liu, 2017; Ajima et al., 2021; Kitajima et al., 2000; Lin et al., 2022).

Previous studies identified a pan-cardiac enhancer of *Smarcd3*, *Smarcd-*F6, which is activated specifically and restrictively in CPCs fated to heart cells shortly after *Mesp1* expression and before expression of other early cardiac-specific TFs (Devine et al., 2014; Yuan et al., 2018). Thus, *Smarcd3*-F6 activity enables the distinction of emerging CPCs from the surrounding mesoderm. We observed *Smarcd3*-F6 activity in posterior regions of *Mesp1* knockout (KO) embryos, indicating persistence of cardiogenesis. Here, we utilized the *Smarcd3*-F6 transgene to comprehensively delineate the dynamic transcriptional and epigenomic consequences of *Mesp1* loss during early cardiogenesis, revealing *Mesp1*-independent aspects of cardiac specification. Our results challenge the dogma of a master regulator for cardiac specification by defining transcriptional phases with different vulnerabilities to *Mesp1* loss.

## RESULTS

### Computational detection of fluorescent transgene reporters enables identification of emerging cardiogenic mesoderm

**Fig. 1.**
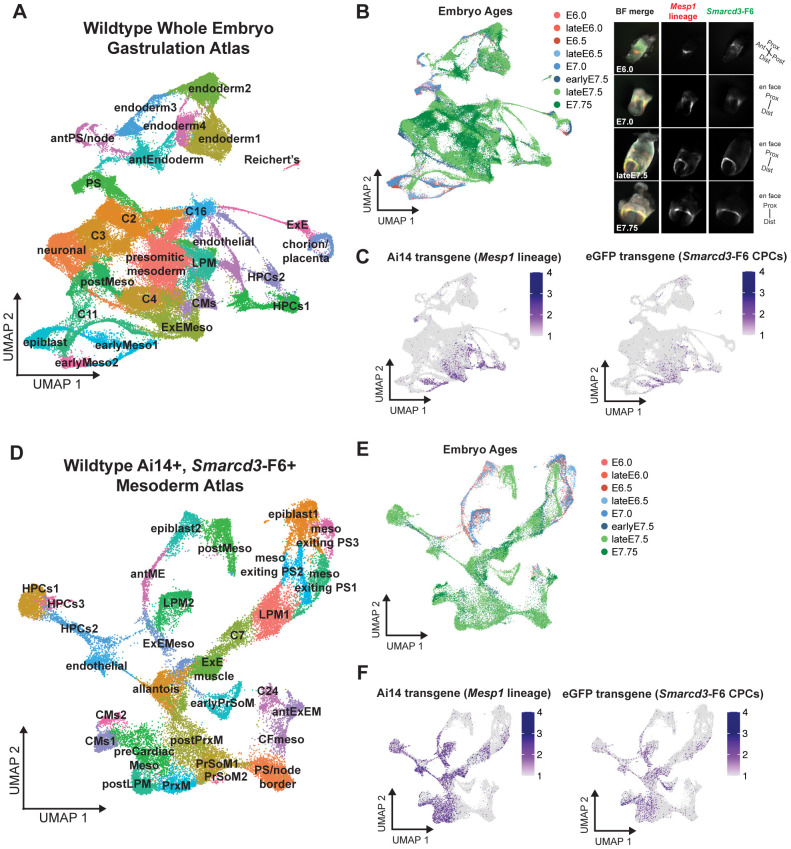
**Identification of the emerging cardiogenic mesoderm using fluorescent transgenes in whole embryo single-cell transcriptomic data.** (A) UMAP of 94,824 cells representing 27 cell types from gastrulating embryos. (B) Left: UMAP labeled with embryo ages. Right: Representative embryo images for endogenous fluorescence from Ai14 (*Mesp1* lineage) and eGFP (*Smarcd3*-F6^+^ CPCs) transgenes. Images not scaled. BF, brightfield. (C) UMAP feature plots for transgene expression in mesodermal cell types. (D) UMAP of 34,724 Ai14^+^, *Smarcd3*-F6^+^ mesodermal cells subsetted atlas, representing 30 cell types. (E) UMAP labeled with embryo ages. (F) UMAP feature plots showing transgene expression. antExEM, anterior extra-embryonic mesoderm; antPS, anterior primitive steak; CFmeso, craniofacial mesoderm; earlyMeso, early mesoderm; ExE, extra-embryonic; Meso, mesoderm; PS/node, primitive streak/node.

**Fig. 2.**
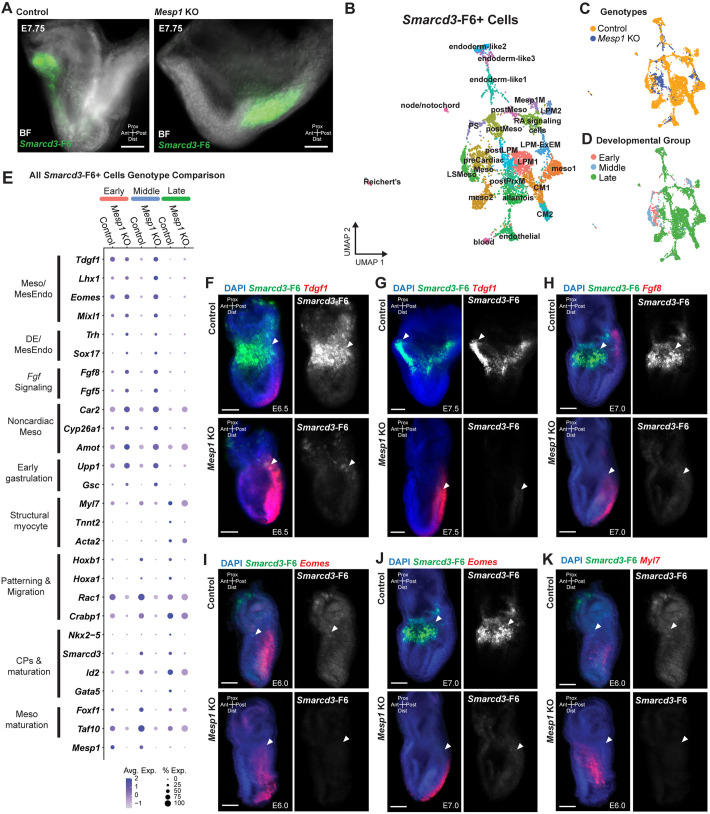
**Transcriptional profiles of *Smarcd3*-F6^+^ cells in *Mesp1* KO embryos.** (A) Fluorescence *in situ* hybridization for *Smarcd3*-F6 expression (green) in cardiac crescent-stage (E7.75) *Mesp1* KO and control littermate embryos. BF, brightfield. (B) UMAP atlas of 4868 *Smarcd3*-F6^+^ cells representing 24 cell types. (C,D) UMAPs colored by genotype (C) and relative developmental stages (D): early (E6.0-E6.5), middle (late E6.5-E7.5), late (late E7.5-early E7.75). (E) Dot plot of DGE across genotypes at relative developmental stages. Dot size represents percentage of cells expressing gene, color represents average expression level. (F-K) Multiplexed fluorescence *in situ* hybridization for *Smarcd3*-F6 (green) and *Tdgf1* (red; F,G) in representative early (F) and middle (G) stages, *Fgf8* (red; H) in middle stages, *Eomes* (red; I,J) in early (I) and middle (J) stages, *Myl7* (red; K) in early stages. Embryos in H and J, and I and K, are the same for each pair of panels, with different channels imaged. Each transcript *in situ* hybridization was repeated in at least two embryos for each genotype. Arrowheads denote *Smarcd3*-F6^+^ cardiogenic regions. Scale bars: 100 μm. Meso, mesoderm; Mesp1M, Mesp1^+^ mesoderm.

To identify emerging early cardiogenic mesoderm cells, we combined transgenic reporter detection with single-cell RNA sequencing (scRNA-seq) on a whole-embryo time course spanning early gastrulation [embryonic day (E) 6.0] until cardiac crescent stages (E7.75) (Fig. 1A,B, Fig. S1). Embryos contained a fluorescent transgene reporter for the *Mesp1* lineage via *Mesp1^Cre^;Rosa26R^Ai14^* (Saga et al., 1999; Madisen et al., 2010), and the *Smarcd3*-F6::eGFP enhancer transgene to mark CPCs constitutively (Devine et al., 2014; Yuan et al., 2018) (Fig. 1C, Fig. S1). We processed whole embryos for scRNA-seq and used computational detection of the reporters to identify the emerging cardiogenic mesoderm (Fig. 1C). Following an annotation of cell types in the whole embryo atlas (Fig. S2A, Table S1A), we subsetted Ai14^+^ and eGFP^+^ cell clusters and demonstrated that these cells resembled the emerging cardiac mesoderm (Fig. 1D-F, Fig. S2B, Table S1B). *Smarcd3*-F6^+^ cell clusters (Figs 1F and 2B, Table S1B) co-expressed early cardiac and mesoderm genes in the mesoderm exiting the primitive streak (meso exiting PS), anterior mesendoderm (antME), and genes of the lateral plate mesoderm (LPM) such as *T*, *Eomes*, *Mesp1*, *Mixl1*, and *Smarcd3* (Fig. 1D, Fig. S3A). *Smarcd3*-F6^+^ cardiomyocytes (CMs) co-expressed cardiac structural genes, such as *Myl7*, *Tnnt2*, and *Actc1* (Fig. 1D-F, Fig. S3B), and cardiac TFs, such as *Tbx5*, *Hand1*, *Nkx2-5*, and *Gata5* (Fig. 1D-F, Fig. S3C). Thus, *Smarcd3*-F6-expressing cells have transcriptional signatures of early CPCs, extending the initial description of this transgene (Devine et al., 2014) by validating high-fidelity demarcation of emerging CPCs within the mesoderm prior to expression of cardiac-specific TFs in scRNA-seq data (Figs S2B and S3). We detected some *Smarcd3*-F6^+^ hematopoietic precursors (HPCs), endothelial cells, and posterior and paraxial mesoderm (postPrxM, PrxM) (Fig. 1D,F). Given that inducible lineage labeling of *Smarcd3*-F6^+^ cells excluded lineage contributions to non-cardiac cell types (Devine et al., 2014) and *Smarcd3* gene expression is reported in the node (Takeuchi et al., 2007), we interpreted reporter detection in these cells to be the result of weak or transient transgene expression such as within cells exiting or adjacent to the embryonic node. We found gene co-expression sufficient to overcome this caveat.

### Transcriptional profiling of *Smarcd3*-F6^+^ cells shows enduring expression of cardiac genes in *Mesp1* KO embryos

To determine the requirement for *Mesp1* in establishing CPC identity, we investigated the transcriptional identities of *Smarcd3*-F6^+^ cells upon loss of *Mesp1*. We detected *Smarcd3*-F6-expressing cells in *Mesp1^Cre/Cre^* (*Mesp1* KO) embryos, although positive cells were localized posteriorly relative to control embryos at early cardiac crescent stages (Fig. 2A, Fig. S4). The persistence of *Smarcd3*-F6^+^ cells led us to hypothesize that these cells represent retained CPCs, suggesting that, as previously described (Saga et al., 2000, 1999; Ajima et al., 2021), aspects of early cardiac specification may be *Mesp1* independent.

To investigate *Mesp1*-dependent and -independent cardiogenic programs*,* we performed scRNA-seq on whole *Mesp1* KO embryos and littermate controls along a timeline of early cardiogenesis spanning early gastrulation (E6.0) to cardiac crescent formation (E7.75) (Fig. S6A). We bioinformatically identified *Smarcd3*-F6-eGFP-expressing cells from the control and *Mesp1* KO whole-embryo time course (Fig. S7A-D, Table S3A) to generate an atlas of 4868 *Smarcd3*-F6^+^ cells representing both genotypes and 24 cell types (Fig. 2B, Fig. S5A, Table S2A). The majority of *Smarcd3*-F6^+^ cells represented early cardiac mesodermal derivatives, such as the late streak mesoderm (LSMeso), *Mesp1*^+^ mesoderm (Mesp1M), posterior mesoderm (postMeso), LPM, precardiac mesoderm (preCardiacMeso), and early CMs (Fig. 2B,C, Fig. S5A). We detected cells of the allantois, LPM/extra-embryonic mesoderm (LPM-ExEM), and the node/notochord, consistent with *Smarcd3* expression in these domains (Fig. 2B, Fig. S5A,B) (Takeuchi et al., 2007; Devine et al., 2014). Similar to our findings in the wildtype (WT) atlas (Fig. 1), we also found populations of blood, endothelial cells, Reichert's membrane, postPrxM, and endoderm-like cells, potentially representing early mesendoderm cells (Fig. 2B, Fig. S5A), suggesting that, as in the WT atlas, transgene detection here results from genotype-agnostic, weak, transient expression (Fig. S5A) (Devine et al., 2014; Takeuchi et al., 2007).

To examine overall trends in differential gene expression (DGE) between *Mesp1* KO and control, we compared all *Smarcd3*-F6^+^ cells between genotypes irrespective of cell type or embryonic stage (Fig. S5B, Table S2B). *Mesp1* KO *Smarcd3*-F6^+^ cells expressed mesodermal genes of the emerging cardiac lineage, such as *Tdgf1*, *Lhx1*, *Eomes*, and *Myl7*, but mostly lacked more mature cardiac progenitor markers, such as *Nkx2-5* (Fig. 2E, Fig. S5A). Dividing the ‘all cells’ genotype analysis into relative developmental stages to separate ‘early’ embryos (E6.0-E6.5), ‘middle’ embryos (late E6.5-E7.5) and ‘late’ embryos (late E7.5 to early E7.75) revealed that genotype discrepancies in cardiac-related gene expression were minor at early stages and diverged with increasing embryonic age (Fig. 2E).

Relatedly, *Mesp1* KO cells were not represented in every *Smarcd3*-F6^+^ cell type (Fig. 2B-D, Fig. S5C). Both genotypes were present in mesoderm clusters (C1, C3), preCardiacMeso (C4), postMeso (C5, C20), retinoic acid signaling cells (C6), LSMeso (C7), allantois (C8), endothelial (C9), postPrxM (C10), the endoderm-like clusters (C11, C14, C19), the LPM-ExEM cluster (C15), the primitive streak (PS) (C17), blood (C21), and Reichert's membrane (C23) (Fig. 2B,C, Fig. S5C). Only control cells were present in LPMs (C0, C16), CMs (C2, C12), posterior lateral plate mesoderm (postLPM) (C13), Mesp1M (C18), and node/notochord (C22). Many of the control-only cell types were late-stage embryo cells (Fig. 2B-D, Fig. S5C), indicating that cell type heterogeneity is affected by loss of *Mesp1* in *Smarcd3*-F6^+^ cells with increasing severity as development progresses. Furthermore, whereas the preCardiacMeso and LSMeso cell types were represented by both genotypes in early- and middle-staged embryos, the late-stage embryo cells represented in the preCardiacMeso were exclusively *Mesp1* KO (Fig. 2B-D, Fig. S5C), indicating retention of precursor transcriptional profiles.

To understand *Mesp1*-correlated differences in emerging *Smarcd3*-F6^+^ CPCs in individual cell types we performed DGE testing within cell types present in both genotypes (Table S2C-F). Within preCardiacMeso and LSMeso cells, we found similar expression of *Tdgf1*, *Eomes* and *Fgf8*, genes involved in early mesoderm specification (Fig. S6A-C) (Probst et al., 2020; Reifers et al., 2000). Multiplexed RNA *in situ* hybridization showed co-expression of these markers with *Smarcd3*-F6 in cardiogenic regions in E6.0-E6.5 (Fig. 2F,I) and E7.0 (Fig. 2H) embryos. Notably, *Mesp1* KO embryos at early stages showed decreased or delayed expression of *Smarcd3*-F6 (Fig. 2H-K), and broad posterior expansion of *Tdgf1* (Fig. 2F) and *Fgf8* (Fig. 2H) expression beyond *Smarcd3*-F6^+^ cardiogenic regions. Additionally, *Tdgf1* and *Eomes* expression aberrantly perdured through E7.5 (Fig. 2G) and E7.0 (Fig. 2J), respectively. Other genes involved in early mesoderm specification (*Fgf10*), lineage specification and pluripotency exit (*Chchd2* and *Nme2*), and non-cardiac mesoderm genes (*Amot*) were upregulated in *Mesp1* KO relative to control cells, whereas genes involved in migration and patterning (*Lefty2*, *Rac1*, *Foxf1*) were downregulated (Fig. S6B,C) (Zhu et al., 2009, 2016; Migeotte et al., 2011; Sang et al., 2021).

We found similar expression levels of *Myl7* between genotypes (Fig. 2E,K), especially within the late stage-dominated LPM-ExEM and endoderm-like1 cells (Fig. S6A,D,E). *Mesp1* KO cells showed upregulation of early mesoderm specification genes (*Tdgf1*, *Eomes*, *Fgf8*, *S100a10*, *Ifitm2*, *Fn1*) and downregulation of morphogenesis and migration genes (*Dlk1*, *Elavl1*) (Fig. S6D,E) (Probst et al., 2020; Cheng et al., 2013; Klymiuk et al., 2012; Saykali et al., 2019; Katsanou et al., 2009).

Collectively, these analyses indicate an increasingly disrupted *Mesp1* KO transcriptional phenotype in developing *Smarcd3*-F6^+^ cells, consistent with divergent morphology at cardiac crescent stages (Fig. 2A-D).

### Alterations to cardiac mesoderm in *Mesp1* KO embryos become increasingly severe as gastrulation progresses

**Fig. 3.**
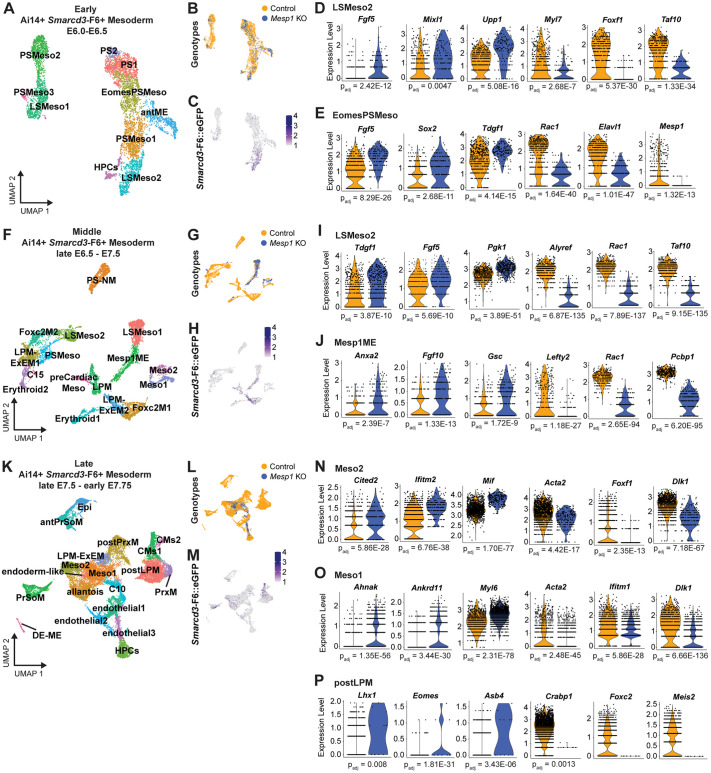
**Transcriptional profiles of cardiac mesoderm in *Mesp1* KO embryos.** (A,F,K) scRNA-seq UMAP atlases of Ai14, *Smarcd3*-F6 double-positive mesoderm cells from early (A; 5504 cells), middle (F; 7666 cells) and late (K; 22,622 cells) developmental embryo stages. (B,C,G,H,L,M) Associated UMAPs colored by genotype (B,G,L) and *Smarcd3*-F6-eGFP expression (C,H,M). (D,E) Early mesoderm DGE in LSMeso2 (D) and EomesPSMeso (E). (I,J) Middle mesoderm DGE in LSMeso2 (I) and Mesp1ME (J). (N-P) Late mesoderm DGE in Meso2 (N), Meso1 (O), and postLPM (P). Significant changes denoted with adjusted *P*<0.05. antPrSoM, anterior presomitic mesoderm; DE-ME, definitive endoderm/mesendoderm; Foxc2M, Foxc2^+^ mesoderm; Meso, mesoderm; PSMeso, primitive streak mesoderm; PS-NM, primitive streak/neuromesodermal.

Following characterization of *Mesp1* KO effects in *Smarcd3*-F6^+^ cells specifically, we investigated potential alterations to the broader mesoderm, inclusive of *Smarcd3*-F6^+^ cells and the cardiac mesoderm marked by Ai14. Dual-reporter transgene identification generated an atlas of 35,792 mesodermal cells from both control and *Mesp1* KO embryos (Fig. S7A-F, Table S3). Similar mesodermal cell types were present in the relative early- and middle-stage embryos from both genotypes, including preCardiacMeso (Fig. S7E-I). However, *Mesp1* KO late-stage embryo cells were not represented in all cell types, with CMs, PrxM, and presomitic mesoderm (PrSoM) cells only present in control (Fig. S7E-I). To resolve how these changes occur during development, we divided the mesoderm dataset into the early, middle, and late developmental stages defined in Fig. 2E. Mesodermal cells for each stage were re-clustered, and DGE was assessed between genotypes (Fig. 3, Table S3).

Within the early mesoderm (Fig. 3A, Fig. S8A, Table S3C), we identified LSMeso2 and *Eomes*^+^ primitive streak mesoderm (EomesPSMeso) as cardiac specification clusters of interest based on enriched *Smarcd3*-F6 expression (Fig. 3C). Both genotypes were present in each cell type (Fig. 3B), indicating that *Mesp1* KO cells are able to exit pluripotency and initiate cardiac mesoderm specification programs. However, *Mesp1* KO cells showed upregulation of mesendoderm and PS markers (*Fgf5*, *Mixl1*, *Upp1*, *Sox2*, *Tdgf1*), downregulation of LPM differentiation genes (*Foxf1*, *Taf10*), downregulation of migration and patterning genes (*Rac1*, *Elavl1*), and persistent but decreased expression of cardiac *Myl7* (Fig. 3D,E, Table S3D,E).

Within the middle mesoderm (Fig. 3F-H, Fig. S8B, Table S3F), we focused on the *Smarcd3*-F6-enriched *Mesp1*^+^ mesendoderm cluster (Mesp1ME) and its developmental predecessors, LSMeso2 cells. Middle-stage LSMeso2 cells (Fig. 3I, Table S3G) showed similar genotype expression trends as early-stage LSMeso cells (Fig. 3D). Mesp1ME *Mesp1* KO cells exhibited upregulation of posterior mesoderm organization genes (*Fgf10*, *Gsc*) (Probst et al., 2020; Meijer et al., 2000; Branney et al., 2009) and the non-cardiac mesoderm gene *Anxa2* (Schwartz et al., 2014; Wang et al., 2015), and downregulation of *Lefty2*, *Rac1*, and the myogenesis differentiation gene *Pcbp1* (Shi and Grifone, 2021) (Fig. 3J, Table S3H). Notably, there was an absence of *Mesp1* KO cells in *Smarcd3*-F6- and *Mesp1-*enriched clusters representing *Foxc2^+^* mesoderm cells (Fig. 3F-H, Fig. S9A). *Foxc2* operates in cardiac field diversification and morphogenesis (Seo and Kume, 2006; Lescroart et al., 2018). Examination of E6.75 embryos by immunohistochemistry and light-sheet imaging showed misaligned anterior-proximal marker domains in *Mesp1* KOs, and an absence of *Foxc2* (Fig. S9). Together, these results indicate reduced cellular diversification, and dysregulation of networks controlling cellular movements and domain boundaries in pre-cardiac-crescent *Mesp1* KO embryos.

Analysis of late mesoderm *Mesp1* KO cells revealed restricted diversity in cardiac and other mesodermal cell types (Fig. 3K,L, Fig. S8C, Table S3I). Furthermore, although both genotypes were present in *Smarcd3*-F6-enriched clusters (Meso1, Meso2) and postLPM, there were no CM *Mesp1* KO cells (Fig. 3K,M). Meso1 and Meso2 *Mesp1* KO cells had highly disrupted transcriptional profiles with upregulation of several mesodermal genes (*Cited2*, *Ifitm2*, *Mif*, *Ahnak*, *Ankrd11*, *Myl6*) (Weninger et al., 2005; Lange et al., 2003; Huang et al., 2022) and downregulation of cardiac maturation genes (*Dlk1*, *Acta2*, *Ifitm1*) (Pursani et al., 2017; Klymiuk et al., 2012) (Fig. 3N,O, Table S3J,K). Additionally, the few *Mesp1* KO cells present in the postLPM cluster showed upregulation of genes involved in mesendoderm specification and organization (*Lhx1*, *Eomes*, *Asb4*) (Fernandez-Guerrero et al., 2021) and downregulation of, or a lack of, patterning, morphogenesis and maturation genes (*Crabp1*, *Foxc2*, *Meis2*) (Fig. 3P, Table S3L).

Thus, late-stage *Mesp1* KO embryos display highly disrupted transcriptional profiles in cardiac mesoderm cells and fail to produce fully heterogeneous mesoderm and mature CMs. Additionally, we observed gross disruption of mesoderm diversification beyond purely cardiogenic cell types in middle- and late-stage *Mesp1* KOs (Fig. 3F-H,K-M, Figs S8, S9), consistent with their altered morphology.

### *Mesp1* KO cardiac mesoderm cells progress incompletely and imperfectly towards cardiomyocyte fates

**Fig. 4.**
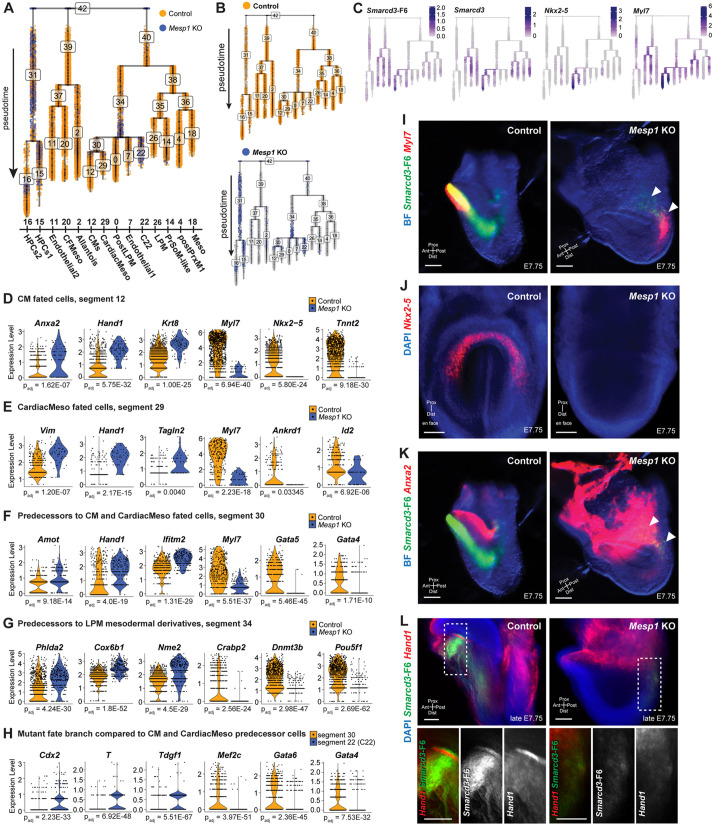
**Pseudotime trajectory analysis of mesoderm fates in *Mesp1* KO embryos.** (A,B) URD pseudotime tree for Ai14^+^, *Smarcd3*-F6^+^ cells' fate progression towards mature mesoderm fates colored by genotypes together (A) and separately (B). (C) Overlay of cardiac marker genes. (D-H) DGE in cells of shared fates and pseudotime identities: (D) CM-fated cells, (E) CardiacMeso-fated cells, (F) predecessors to CM and CardiacMeso fates, (G) predecessors to LPM derivate fates, (H) comparison of mutant fate branch C22 to CardiacMeso fate predecessors. (I,J) Multiplexed fluorescence *in situ* hybridization for *Smarcd3*-F6 (green) and *Myl7* (I), *Nkx2-5* (J), *Anxa2* (K), and *Hand1* (L) (all in red) in cardiac crescent-stage embryos. Embryos in I and K are the same, with different channels imaged. Each transcript *in situ* hybridization was repeated in at least two embryos for each genotype. Arrowheads denote *Smarcd3*-F6^+^ cardiogenic regions in *Mesp1* KO embryos. Boxed regions in L are shown at higher magnification and with separate channels below. Scale bars: 100 μm. CFMeso, craniofacial mesoderm; Meso, mesoderm.

We next investigated cardiac fate progression to understand how *Mesp1* KO embryos initiate cardiogenesis, but fail to produce mature CMs. Utilizing pseudotemporal trajectory ordering with the R package URD (Farrell et al., 2018) on the full mesoderm dataset (Fig. S7E), we defined epiblast cells, which contained cells from the earliest stage embryos, as the root (C1-Epiblast in Fig. S7E,G), and clusters containing the oldest stage embryos and the most differentiated mesodermal cells as the tips (Fig. 4A, Fig. S7E,G, Fig. S10A,B). We layered *Smarcd3*-F6-eGFP expression within tree space to identify the main cardiogenic fate paths, which co-express CM genes such as *Nkx2-5*, *Myl7*, and *Smarcd3* (Fig. 4C, Fig. S10C). Within CM and cardiac mesoderm (CardiacMeso) fate branches, *Mesp1* KO cells occupied the youngest pseudotemporal positions near the top of the branch segment and were represented more in younger pseudotime segment branches of the tree, including their own earlier-pseudotime branch fate ‘C22’, which was defined by multiple mesodermal genes not representative of any previously characterized cardiac mesoderm cell type (Fig. 4A,B, Fig. S7E,I).

Beginning at segment 34, we performed DGE analysis to compare cell types of similar fate potentials within branch segments or pseudotemporal levels of the trajectory (Fig. 4D-H). Among CM-fated cells, *Mesp1* KO cells had enriched expression for *Anxa2*, *Hand1*, *Krt8* and other extra-embryonic mesoderm genes, expressed lower levels of structural myocyte genes such as *Myl7* and *Tnnt2* relative to control, and lacked *Nkx2-5* (Fig. 4D, Table S4A). *Hand1* was similarly enriched in *Mesp1* KO CardiacMeso-fated cells, along with *Vim*, a fibroblast gene, and *Tagln2*, a gene involved in transformation and cell morphology (Fig. 4E, Table S4B). In the CardiacMeso-fated segment, *Myl7* and *Id2* were reduced relative to controls, as was *Ankrd1*, a gene implicated in sarcomere-binding and dilated cardiomyopathy that is known to be upregulated with overexpression of *Mesp1* (Bondue and Blanpain, 2010; Moulik et al., 2009) (Fig. 4E, Table S4B). In the segment 30 branch giving rise to CM and CardiacMeso fates, *Amot*, *Hand1*, and *Ifitm2*, all genes expressed in the posterior proximal extra-embryonic border of the murine embryo and extra-embryonic mesoderm (ExEMeso), were upregulated in *Mesp1* KO cells (Fig. 4F, Table S4C). By contrast, the myocyte and cardiac progenitor genes *Myl7*, *Gata5*, and *Gata4* were decreased in *Mesp1* KO cells relative to control (Fig. 4F, Table S4C). In *Mesp1* KO cells in segment 34, the predecessors to LPM mesodermal derivatives, the pronephros gene *Cox6b1*, the spongiotrophoblast and extra-embryonic energy storage gene *Phlda2*, and the embryonic stem cell self-renewal gene *Nme2* (Zhu et al., 2009) were enriched, whereas the retinoic acid gene *Crabp2* and the early gastrulation genes *Dnmt3b* and *Pou5f1* were downregulated (Fig. 4G, Table S4D). Finally, we compared the *Mesp1* KO cell-dominated segment 22 with its pseudotime-branching contemporary segment 30, and found enriched expression of the mesoderm fate-promoting gastrulation TFs *Cdx2* and *T*, along with the mesendoderm allocation gene *Tdgf1* (Fig. 4H, Table S4E). Conversely, the cardiac progenitor morphogenesis TFs *Mef2c*, *Gata6*, and *Gata4* were downregulated (Fig. 4H, Table S4E). These results indicate that *Mesp1* KO CPCs reach a cardiogenic breakpoint during gastrulation prior to cardiac crescent formation in part through a failure to express the requisite TFs.

Thus, *Mesp1* KO cardiac mesoderm fates are characterized by: (1) retained expression of some cardiac progenitor genes (*Myl7*, *Gata4/5/6*, *Id2*, *Tnnt2*), albeit at decreased levels relative to control, and absence of others (*Nkx2-5*, *Ankrd1*), and (2) ectopic enrichment of ExEMeso and other mesoderm-associated genes (*Hand1*, *Anxa2*, *Amot*, *Vim*, *Tagln2*). We used multiplexed fluorescent RNA *in situ* hybridization to validate the spatial domains of differentially expressed genes in late stages, and confirmed the presence of *Myl7*^+^ cells co-expressing *Smarcd3*-F6 in the posterior distal compartment of *Mesp1* KO embryos (Fig. 4I), along with absence of *Nkx2-5* expression (Fig. 4J). We also found ectopic *Anxa2* expression in the embryo proper, overlapping with *Smarcd3*-F6^+^ cells in their posterior position in *Mesp1* KO embryos, in contrast to the anterior extra-embryonic-restricted expression pattern of controls (Fig. 4K). Although *Hand1* was expressed at similar levels in the extra-embryonic regions of both genotypes, *Hand1* was diffusely co-expressed with *Smarcd3*-F6^+^ posterior cells in *Mesp1* KO embryos yet appeared uniquely enriched in the juxtacardiac field of control embryos only (Fig. 4L). These *in vivo* trajectory inference and *in situ* results are consistent with live cell tracking in *Mesp1* KO embryos (Dominguez et al., 2023) wherein cells exit the primitive streak, allocation of embryonic and extra-embryonic mesoderm are distinct, but mutant embryonic mesoderm cells exhibit defective migration and incoherent directionality with downregulation of migratory genes, such as *Rac1* (Table S4). Therefore, *Mesp1* KO *Smarcd3*-F6^+^ cells likely represent mutated cardiac lineage cells within the embryo proper, as opposed to these cells adopting an alternative extra-embryonic fate.

### scATAC-seq analysis reveals a regulatory barrier in *Mesp1* KO mesoderm progression towards cardiomyocyte fates

**Fig. 5.**
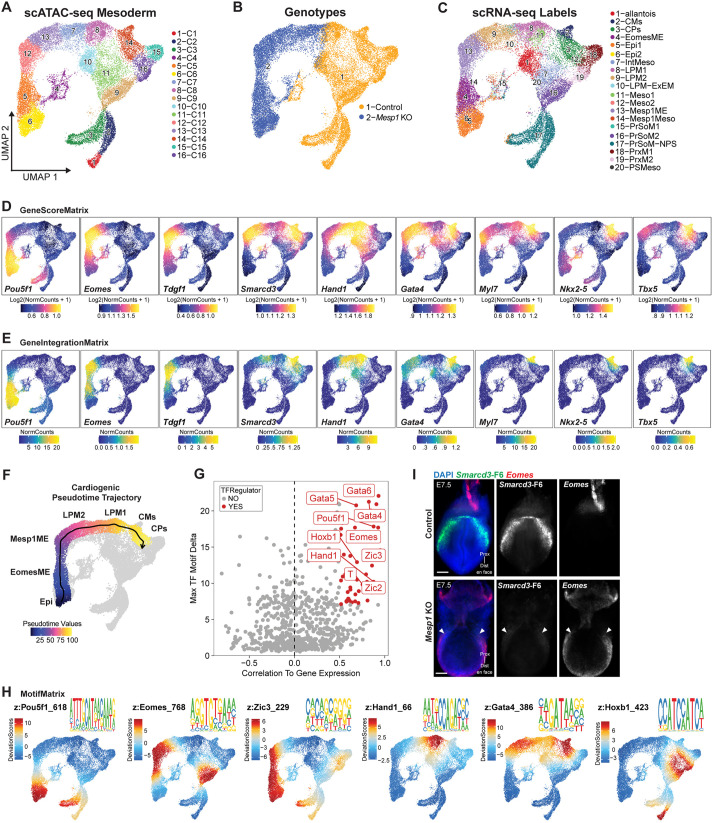
**Characterizing transcriptional drivers in *Mesp1* KO mesoderm during cardiogenesis.** (A-C) Mesoderm scATAC-seq atlas of 16 cell types (A) with UMAPs for genotypes (B) and relative cell annotations from integration with scRNA-seq (C). (D,E) GeneScoreMatrix plots for chromatin accessibility loci (D) and GeneIntegrationMatrix plots for scRNA-seq integrated cardiac mesoderm marker and TF gene expression (E). (F) *Mesp1* KO cardiac-fate trajectory path colored by pseudotime values. (G) Positive TF regulatory drivers (red) identified by maximum z-score deltas from motif variance between clusters correlated to gene expression within clusters. (H) Positive regulatory TFs' motif position weight matrix (PWM) plots with z-scores mapped in UMAP space. (I) Multiplexed fluorescence *in situ* hybridization for *Smarcd3*-F6 (green) and *Eomes* (red) in cardiac crescent-stage embryos. *In situ* hybridizations were repeated three times in embryos of each genotype. Arrowheads denote cardiogenic regions in *Mesp1* KO embryo. Scale bars: 100 μm. IntMeso, intermediate mesoderm; Meso, mesoderm; Mesp1Meso, Mesp1^+^ mesoderm; PrSoM-NPS, presomitic mesoderm/primitive streak bordering node; PSMeso, primitive streak mesoderm.

To characterize the regulatory landscape prohibiting *Mesp1* KO cells from progressing fully towards CM fates, we turned to single-cell assay for transposase accessible chromatin (scATAC-seq) (Buenrostro et al., 2015) of middle- and late-stage embryos aged E7.5-E7.75 (Fig. S11). We processed whole embryos and performed preliminary atlasing analysis using the R analysis package ArchR (Granja et al., 2021). We integrated the complementary whole embryo scRNA-seq dataset with chromatin accessibility profiles near genes (gene scores) to subset mesodermal cell-type clusters (Fig. S12A-D, Table S5A) in order to generate a subset scATAC-seq atlas of 16 mesodermal cell types (Fig. 5A). *Mesp1* KO and controls had strikingly divergent regulatory landscapes (Fig. 5B). *Mesp1* KO cells were confined to scATAC-seq clusters representing epiblast (Epi), mesendoderm, and LPM cell types, whereas control cells were represented in the LPM cell types, the more mature cardiac progenitor (CP) and CM cluster, and mesodermal derivative cell types (Fig. 5A-C). Integration with the complementary mesoderm scRNA-seq dataset (Table S5B), visualization of key marker gene scores and integrated expression (Fig. 5D,E), and Jaccard indexing (Fig. S13) were used to assign relative cell identities to each mesoderm scATAC-seq cluster (Fig. 5C). Although some cardiac TFs such as *Nkx2-5* were not active in *Mesp1* KO cells, others such as *Tbx5* exhibited chromatin accessibility in *Mesp1* KO cells but integrated gene expression only in control CM/CP cells (Fig. 5B,D,E). The cardiac TFs *Hand1* and *Gata4* had similar activity between *Mesp1* KO and control cells (Fig. 5B,D,E), and although *Mesp1* KO cells exhibited downregulation of *Smarcd3* and *Myl7* expression, chromatin accessibility for these genes was similar between genotypes (Fig. 5B,D,E). These results indicate a perdurance of active chromatin states in the transcriptional steps preceding cardiogenic differentiation.

To interrogate the developmental differences of *Mesp1* KO cells failing to mature, we performed an ArchR trajectory inference analysis assessing pseudotime along the cardiac fate path. We defined a trajectory backbone in the *Mesp1* KO cells traversing the expected differentiation path of Epi, *Eomes*^+^ mesendoderm (EomesME), Mesp1ME, lateral plate mesoderm (LPM2, LPM1), to CP and CM clusters. This revealed that although *Mesp1* KO cells traversed the expected path from epiblast to LPM, they abruptly failed to progress further towards CPs and CMs (Fig. 5F). Notably, the most mature cell identity *Mesp1* KO cells achieved (LPM1) also contained control cells capable of progressing to CPs past this point (Fig. 5F, Fig. S14), indicating that the LPM1-to-CP transition represents the breakpoint in cardiogenesis for *Mesp1* KO cells (Fig. 5F).

We assessed dynamic shifts in the correlations across pseudotime between TF gene scores and gene expression with corresponding TF motifs in accessible chromatin peaks (Fig. S15) to reveal a biologically sensical order of TF regulators involved in cardiogenesis. Notably, TFs represented in early pseudotime and *Mesp1* KO cells (*Lhx1*, *T*, *Eomes*, *Zic2/3*, *Pitx2*, *Isl1*; Fig. S15) are consistent with early mesodermal regulatory networks, indicating that aspects of these networks are either *Mesp1* independent or resilient to *Mesp1* loss. TFs represented in later pseudotime (*Gata4/5/6*, *Hoxb1*; Fig. S15) are concordant with downregulated gene expression in *Mesp1* KO CPCs and mesoderm by scRNA-seq (Figs 2-4), suggesting that failed induction of these TFs and their programs is either *Mesp1* dependent or vulnerable to secondary effects of *Mesp1* loss.

To ascertain cell type- and genotype-specific gene regulatory networks along the cardiogenic trajectory, we performed an orthogonal analysis to identify putative positive transcriptional drivers (Fig. 5G, Table S5C) and visualized the resulting TFs' motif enrichments in uniform manifold approximation and projection (UMAP) space (Fig. 5H, Fig. S15). In particular, the *Mesp1* KO Epi cluster is driven in part by the pluripotency TF POU5F1 and the MESP1 co-factor ZIC3 (Lin et al., 2022) (Fig. 5D,E,H). Mesendoderm TFs EOMES and ZIC3 are drivers of EomesME and Mesp1ME (Fig. 5D,E,H). The ExEM and first heart field TF HAND1 appeared in the ‘last-stop’ LPM1 cell types where *Mesp1* KO cells failed to progress (Fig. 5D,E,H), consistent with expression observed in *Mesp1* KO CM-fated cells (Fig. 4D-F,L). Although GATA motifs were present in LPM2 and LPM1, the latter of which contained both genotypes, GATA4 was particularly enriched in the later CP and CM fate destinations (Fig. 5H). This result, coupled with representation of *Gata4* in late trajectory pseudotime (Fig. S15A) and downregulated expression in cardiac-fated *Mesp1* KO mesoderm cells (Fig. 4F), likely signifies *Mesp1*-dependent induction and/or influence of GATA factor-associated networks within emerging CMs. Indeed, *Gata4* was shown to be activated by MESP1 during *in vitro* differentiation (Soibam et al., 2015), and although GATA4 binds the minority of MESP1-bound enhancers, it binds nearly half of enhancers opened following *in vitro* induction of *Mesp1* (Lin et al., 2022). Separately, the motif of the MESP1 target gene *Hoxb1* was distinctly present in PrSoM cell types, coincident with induction under ‘late phase’ MESP1 activity (Lin et al., 2022; Haraguchi et al., 2001) for non-cardiac mesoderm diversification (Fig. 5H). The preponderance and accordance of these results support the explanation that early cardiogenic phases proceed resilient to *Mesp1* loss; however, *Mesp1* KO cells cannot proceed to later phases.

Given the enrichment of EOMES motifs, gene score and gene expression in mesendoderm clusters (Fig. 5D,E,I), the apparent role of this TF as a positive regulatory driver (Fig. 5G,I), and its direct involvement in *Mesp1* induction (Tosic et al., 2019; Costello et al., 2011; Alexanian et al., 2017; Guo et al., 2018; Probst et al., 2020), we investigated *Eomes* as a potential driver of *Mesp1-*independent early phases of cardiogenesis. EOMES directly binds *Myl7* regulatory regions (Tosic et al., 2019) and *Eomes* loss disrupts induction of *Myl7* (Costello et al., 2011), suggesting that expression of *Myl7* in *Mesp1* KO CPCs (Fig. 2E,K, Fig. S6A,C-E, Fig. 3D, Fig. 4C-F,J) is regulated by *Eomes* at least partially independently of *Mesp1*. Furthermore, domains of *Eomes* expression anomalously endured in cardiogenic *Smarcd3*-F6^+^ regions and were ectopically expanded in lateral aspects of the embryo proper in cardiac crescent-staged *Mesp1* KOs (Fig. 5I), indicating that improper repression of *Eomes* in cardiogenic regions may underlie the halt in cardiogenesis progression.

### The disrupted regulatory landscape of *Mesp1* KO embryos is characterized by ectopic endurance of mesendoderm gene programs

**Fig. 6.**
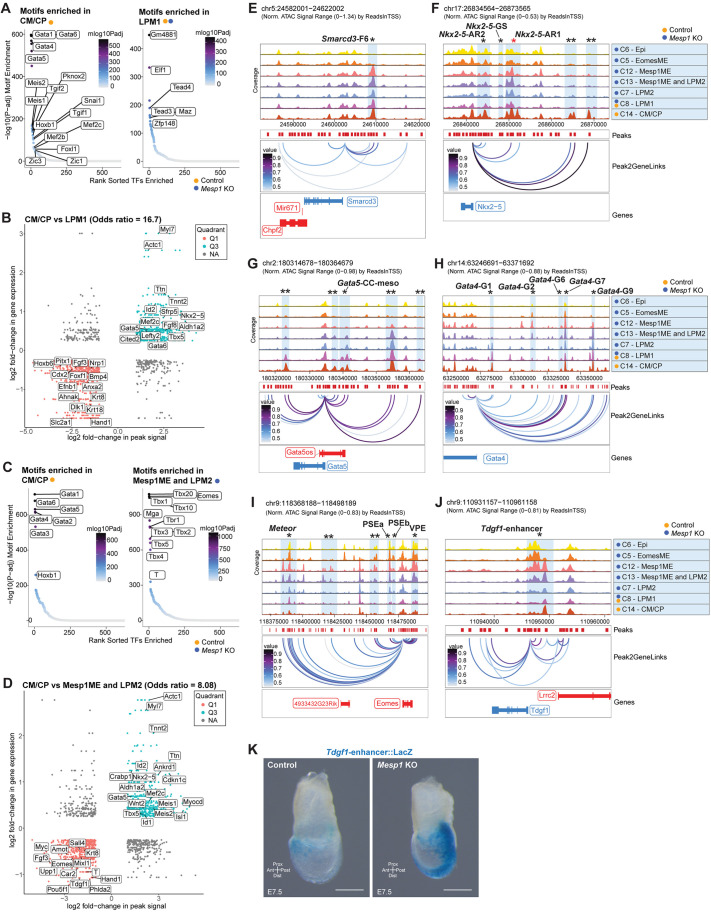
**Disrupted regulatory landscape of *Mesp1* KO mesoderm.** (A,C) Motifs enriched in differentially accessible peaks between CM/CP versus LPM1 (A) and CM/CP versus Mesp1ME and LPM2 (C). (B,D) (Peak,gene) association plots for CM/CP versus LPM1 (B) and CM/CP versus Mesp1ME and LPM2 (D). Plots show correlations between differential peak accessibility and gene expression in cell type1 versus type2. Q3 (peak, gene) pairs are significantly more accessible peaks paired with upregulated gene expression in type1 cells. Q1 (peak,gene) pairs are significantly more accessible peaks paired with upregulated gene expression in type2 cells. Odds ratio denotes observed (peak,gene) relationship probability. (E-J) Cell type genome tracks for Peak2Gene linkage predictions of regulatory connections between distal accessible regions (peaks) and nearby genes. Blue vertical bars denote predicted distal regulatory regions; single black asterisks denote characterized elements, named when available; red asterisk denotes regions with MESP1 binding; double asterisks denote uncharacterized elements. (E) *Smarcd3* linkage with F6 enhancer. (F-J) Peak linkages to genes *Nkx2-5* (F), *Gata5* (G), *Gata4* (H), *Eomes* (I), and *Tdgf1* (J)*.* (K) X-gal stain for activity of characterized *Tdgf1* enhancer, four replicate embryos per genotype. Scale bars: 200 μm.

To understand how the *Mesp1* KO disrupted regulatory landscape underlies transcriptional barriers to cardiac fate progression, we characterized cell type peak accessibility profiles and motif enrichment (Fig. S16A,B). We performed differential accessibility testing of peaks between cell types along the cardiac trajectory (Fig. S16C-G). Focusing specifically on the ‘last stop’ for *Mesp1* KO cells, we compared motif enrichment within differential peaks of CMs/CPs containing only control and LPM1 containing both control and *Mesp1* KO cells (Fig. 6A, Table S6A). In agreement with motif enrichment scores for positive TF regulators (Fig. 5G,H), GATA and MEF2C motifs were enriched in more mature CM/CPs, whereas motifs for cardiac differentiation and myogenesis-promoting TEAD factors were relatively enriched within LPM1, further substantiating retention of myocyte identity within *Mesp1* KO CPCs (Fig. 6A, Table S6A, Fig. 2E,K, Fig. S6D, Fig. 4I) (Han et al., 2020; Akerberg et al., 2019). We next performed an association analysis, and found that significantly differentially open peaks corresponded to upregulated gene expression with an odds ratio of 16.7 (Fig. 6B, Q3, Table S6B), whereas significantly differentially closed peaks corresponded to downregulated gene expression (Fig. 6B, Q1, Table S6B) in CMs/CPs relative to LPM1.

We next compared control-only CMs/CPs with *Mesp1* KO-only Mesp1ME and LPM2 (Fig. 6C,D), because these cells had similar gene scores for the *Smarcd3* locus (Fig. 5D), a proxy for *Smarcd3*-F6 enhancer activity. Motifs including those for GATA and HOX factors were relatively enriched in CMs/CPs, and T-box motifs including EOMES and T were enriched in Mesp1ME and LPM2 (Fig. 6C, Table S6C). A correlation odds ratio of 8.08 highlighted corresponding peak accessibility and gene expression enrichment for CP patterning and CM genes in control CMs/CPs (Q3: *Nkx2-5*, *Tbx5*, *Wnt2*, *Mef2c*, *Meis1*, *Ttn*, *Tnnt2*) (Fig. 6D, Table S6D) and relatively enriched peak accessibility near upregulated genes for earlier cardiac mesoderm and mesendoderm programs in Mesp1ME and LPM2 *Mesp1* KO-only cells (Q1: *Tdgf1*, *Fgf3*, *Eomes*, *Mixl1*, *T*, *Krt8*, *Hand1*, *Pou5f1*) (Fig. 6D, Table S6D).

Applying this paradigm to multiple pairwise comparisons along the cardiogenic trajectory (Fig. S16H-M, Table S6E-J) showed that the predominant regulatory signature of control CMs/CPs is characterized by TFs such as *Gata4/5/6*, *Hoxb1*, *Mef2c*, *Foxf1*, and *Tbx5*, which are required for initiation of cardiac patterning and morphogenesis programs upon formation of the cardiac crescent, subsequent heart fields, and higher-level organogenesis (Pikkarainen et al., 2004; Kokkinopoulos et al., 2015; Bruneau, 2013; Stefanovic et al., 2020; Harvey, 2002; Kelly et al., 2014). *Mesp1* KO cells were unable to activate these same regulatory programs, instead retaining TFs for mesendoderm and other mesoderm networks (*T*, *Eomes*, *Hand1*) (Fig. S16H-M, Table S6E-J). In all evaluated comparisons of motif enrichment in differentially accessible peaks, MESP1 was never amongst the highest ranked motifs (Fig. 6A,C, Fig. S16H,J,L, Table S6A,C,E,G,I).

To visualize regulatory interactions between chromatin accessibility and integrated gene expression agnostic of differential accessibility and expression testing, we utilized the ArchR pipeline's orthogonal ‘peak2gene’ linkage approach (Granja et al., 2021). This identified both known and uncharacterized distal regulatory elements (Fig. 6E-J). The *Smarcd3*-F6 enhancer (Devine et al., 2014), as expected, showed linkage to *Smarcd3* and similar accessibility across genotypes and cardiogenic cell types, including the *Mesp1* KO cells Mesp1ME, LPM2 (Fig. 6E), consistent with our detection of the transgene by scRNA-seq.

In accordance with the modular enhancer landscape of *Nkx2-5*, multiple peak linkages were defined for the *Nkx2-5* locus, including two distal uncharacterized regions (Fig. 6F). Two linkages were appropriately mapped to the characterized GATA4-, NFAT-, MESP1/MZF1- and ISL1-regulated 9 kb-upstream *Nkx2-5* cardiac enhancer sequence (*Nkx2-5*-AR1) (Lien et al., 1999; Chen and Cao, 2009; Clark et al., 2013; Doppler et al., 2014; Bondue et al., 2008) and the distal-linked AR1 peak was increased in control CM/CPs only (Fig. 6F). Similarly, the GATA4-, SMAD4-, NFAT-, ISL1-regulated *Nkx2-5*-AR2 enhancer (Searcy et al., 1998; Liberatore et al., 2002; Lien et al., 2002) and the two uncharacterized linked regions ∼25 kb and ∼30 kb-upstream of the transcription start site (TSS) showed enriched accessibility in control CM/CPs (Fig. 6F), whereas the GATA4- and SMAD1/4-responsive 6 kb-upstream enhancer (*Nkx2-5*-GS) (Brown et al., 2004) did not show accessibility in any cells (Fig. 6F). These results are consistent with the absence of *Nkx2-5* in *Mesp1* KO embryos (Fig. 4), underscore the complexity of regulation on this crucial cardiac TF, and provide further evidence for the regulatory shift between LPM1 and CM/CP cells (Fig. 5F) that *Mesp1* KOs are unable to progress through.

Examination of the *Gata5* locus reveals a linkage to the characterized cardiac crescent and mesodermal derivatives enhancer (*Gata5*-CC-meso) (MacNeill et al., 2000) as well as several uncharacterized linked distal elements with accessibility in Mesp1ME, LPM2, LPM1, and CM/CP cells (Fig. 6G). Several characterized *Gata4* enhancer regions were linked, including lateral mesoderm enhancer *Gata4*-G2 (Rojas et al., 2005) and cardiac crescent enhancer *Gata4*-G9 (Schachterle et al., 2012). The FOXF1 and GATA4-bound enhancer *Gata4*-G2 showed enriched accessibility in mesendoderm *Mesp1* KO cells, whereas ETS-activated *Gata4*-G9 was similarly accessible between Mesp1ME, LPM2, LPM1, and CM/CPs (Fig. 6H), highlighting retention of active chromatin states preceding cardiac patterning and differentiation despite loss of *Mesp1*.

Evaluation of the *Eomes* and *Tdgf1* loci, which ectopically perdure in *Mesp1* KO embryos, showed a corresponding pattern of enriched linked peaks in *Mesp1* KO cell types (Fig. 6I). *Meteor*, a distal lncRNA (Alexanian et al., 2017), was linked to *Eomes* with greatest accessibility enrichment in Epi, EomesME, and Mesp1ME *Mesp1* KO cells (Fig. 6I). Similarly, the PSEa, PSEb, and VME regulatory regions (Simon et al., 2017) were linked (Fig. 6I), substantiating an intact early cardiac mesoderm transcriptional landscape despite *Mesp1* absence; however, the early cardiac mesoderm transcriptional landscape was retained later in development than it should be for the age of these embryos. Upstream of *Tdgf1*, a previously characterized enhancer and direct MEF2C target (Barnes et al., 2016) displayed enrichment of proximal peaks in *Mesp1* KO cells (Fig. 6J), which we confirmed by increased *Tdgf1* enhancer transgene activity in posterior domains of E7.5 *Mesp1* KO embryos (Fig. 6K). Increased *Tdgf1* enhancer activity mimicked the enriched *Tdgf1* gene expression in *Mesp1* KO embryos (Fig. 2F,G), further supporting the hypothesis that early programs are de-repressed in the absence of *Mesp1*.

Examining additional linked peak profiles around differentially expressed genes revealed linkages between *Mesp1* and the characterized ‘EME’ enhancer (Haraguchi et al., 2001; Ajima et al., 2021; Costello et al., 2011; Guo et al., 2018) with enrichment in *Mesp1* KO cells, likely indicative of retained chromatin landscape or de-repression of the locus without appropriate regulation from downstream targets (Fig. S17A). We detected linkages to three uncharacterized distal regions near the *Gata6* locus, as well as the NKX2-5-targeted enhancers (Molkentin et al., 2000) with similar accessibility profiles across *Mesp1* KO Mesp1ME, LPM2 cells and the LPM1 cells containing both genotypes (Fig. S17B). We note linkages to multiple characterized *Hand1* enhancer regions (Vincentz et al., 2021, 2019; George and Firulli, 2021) across both genotypes and multiple cell types, with accessibility for some enhancers decreased in CM/CPs (Fig. S17C), consistent with activity and expression of *Hand1* in LPM1 (Fig. 5D,E,H). Peaks with similar accessibility across Mesp1ME and LPM cell types containing both genotypes were detected in linkages near *Tbx5*, including the Tbx5-CRE16 (Fig. S17D) (Smemo et al., 2012), and downregulated but retained structural myocyte genes *Tnnt2* (Fig. S17E) and *Myl7* (Fig. S17F). Increased accessibility for *Anxa2-*linked peaks in *Mesp1* KO LPM2 cells and control/*Mesp1* KO LPM1 cells was in contrast with near-inaccessibility in control CM/CPs (Fig. S17G), consistent with the upregulated *Anxa2* expression in late-stage embryo cardiogenic regions (Fig. 4K). Downstream distal peaks were linked to the *Mesp1*-induced EMT-gene *Snai1* (Fig. S15H) (Lin et al., 2022), including the MESP1-binding site. These peak2gene linkage analyses further illustrate the correlation between differential gene expression and the altered chromatin landscape in *Mesp1* KO mesoderm cells preventing progression towards mature cardiac fates while highlighting the retention of some distal-regulatory elements relevant to early cardiogenesis.

### MESP1-dependent and -independent transcription factor binding underlies differential genome accessibility between *Mesp1* KO and control cells

**Fig. 7.**
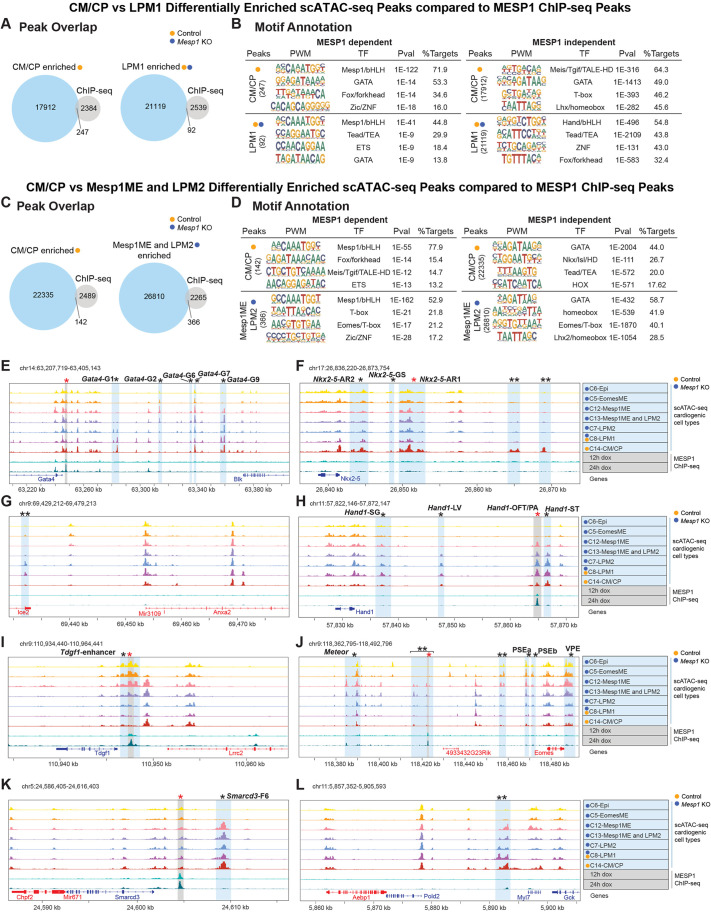
**MESP1 activity partially explains dysregulated regulatory networks in *Mesp1* KO mesoderm.** (A,C) MESP1 ChIP-seq compared with enriched peaks in differentially accessible regions between control and mutant cell types in CM/CP versus LPM1 (A) and CM/CP versus Mesp1ME and LPM2 (C). (B,D) TF binding motif annotations for MESP1-dependent and -independent differentially accessible regions. (E-L) Genome tracks of cell type scATAC-seq profiles with MESP1 ChIP-seq. Vertical bars in blue denote predicted distal regulatory elements; gray denotes MESP1 binding by ChIP-seq; single black asterisks denote characterized elements, named when available; red asterisks denote reported MESP1-binding; double asterisks denote uncharacterized elements.

To assess whether differentially accessible regions between *Mesp1* KO and control cells were a consequence of disrupted MESP1-binding sites, we compared scATAC-seq peak accessibility with an *in vitro* MESP1 ChIP-seq time series from mouse pluripotent stem cells with doxycycline-induced *Mesp1* expression at 12 h and 24 h (Lin et al., 2022). MESP1 ChIP-seq peaks overlapped with 1.3% (247/18,159) of CM/CP-enriched scATAC-seq peaks, compared to 0.43% (92/21,211) of LPM1-enriched peaks (Fig. 7A). Motif annotation of MESP1-dependent overlapping regions highlighted MESP1/bHLH and GATA factor sites in both CM/CP and LPM1 cells (Fig. 7B). Consistent with differential gene expression and peak accessibility (Fig. 6B), GATA and T-box motifs were present in MESP1-independent CM/CP-enriched peaks, and HAND motifs were enriched in LPM1 (Fig. 7B). In the comparison of cells with similar *Smarcd3* gene scores, 0.6% (142/22,477) enriched CM/CPs peaks overlapped with MESP1 peaks, relative to 1.3% (366/21,176) of peaks in Mesp1ME and LPM2 cells (Fig. 7C). MESP1 motifs were represented in MESP1-dependent CM/CP-enriched peaks, whereas, similar to Lin et al. (2022), MESP1 and ZIC motifs appeared in Mesp1ME- and LPM2-enriched peaks (Fig. 7D). GATA- and homeobox factor-binding sites appeared in both cell groups' enriched MESP1-independent peaks, and EOMES/T-box motifs were present in both MESP1-dependent and -independent Mesp1ME- and LPM2-enriched peaks (Fig. 7D). Similar peak overlap and motif binding trends were present across comparisons between genotypes along the cardiogenic trajectory (Fig. S18).

### Distal MESP1-dependent binding is associated with de-repression of mesendoderm genes

We next evaluated *in vitro* MESP1 ChIP-seq data (Lin et al., 2022) in conjunction with our scATAC-seq predicted enhancer regions near mutant-dysregulated genes. MESP1 bound directly upstream of mutant-downregulated *Gata4*, particularly evident in the ‘late induction’ 24 h MESP1 peak (Fig. 7E) (Lin et al., 2022), but no MESP1 binding was detected at previously reported binding regions near *Nkx2-5* (Fig. 7F). MESP1 binding distally upstream in mutant-enriched accessible regions near *Hand1* (Fig. 7H), *Eomes* (Fig. 7J), and within the *Tdgf1* enhancer (Fig. 7I) suggests that de-repression is associated with upregulation of these mesendoderm genes in *Mesp1* KOs (Fig. 5). Although MESP1 does not bind *Smarcd3*-F6, MESP1 bound proximal to the *Smarcd3* TSS where chromatin accessibility was similar across mutant and control cells (Fig. 7K). No MESP1 binding peaks appeared near mutant-upregulated *Anxa2* (Fig. 7G) nor mutant-expressed but downregulated *Myl7* (Fig. 7L).

## DISCUSSION

**Fig. 8.**
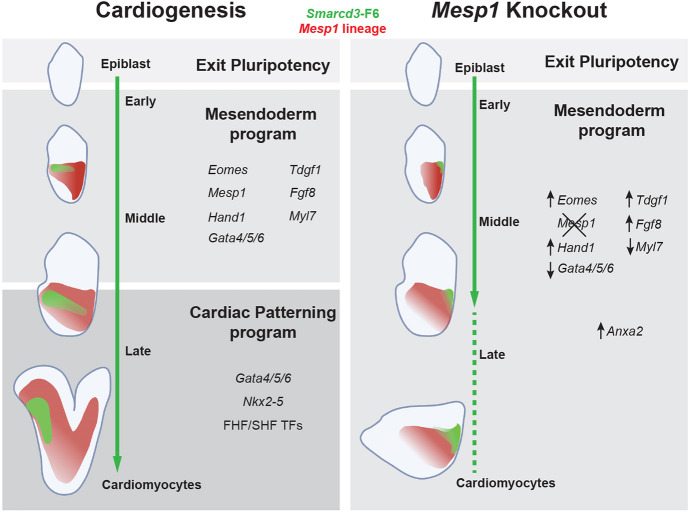
**Model for transcriptional regulatory landscape of cardiogenesis and loss of *Mesp1*.** Schematic model of gene regulatory program phases during cardiac lineage specification and differentiation. *Mesp1* KO cardiac mesoderm cells exit pluripotency, induce early cardiac specification genes under control of mesendoderm programs, yet fail to activate crucial cardiac TFs at cardiac crescent stages to initiate cardiac patterning programs.

We generated scRNA-seq and scATAC-seq datasets from whole mouse embryos in a timeline of gastrulation, creating a valuable *in vivo* resource for high-resolution studies of gene regulatory networks in early embryonic development. We utilized computational detection of CPC-labeling transgenes to show that while *Mesp1* KO embryos are capable of initiating and progressing through early cardiac mesoderm specification, a *Mesp1*-dependent regulatory barrier prevents *Mesp1* KO CPCs from progressing completely towards CM fates. We characterized improper repression of early mesendoderm programs at this breakpoint, such as how absence of *Mesp1* leads to enduring *Eomes* activity, which in turn promotes ectopic perdurance of mesendoderm transcriptional networks when embryos should instead be upregulating cardiac patterning programs. Additionally, this disrupted regulatory landscape likely contributes to *Mesp1* KO cardiac mesoderm and CPCs ectopically expressing non-cardiac mesoderm genes. Despite this ectopic expression, CPCs do not appear to deviate from a cardiac-directed mesodermal fate path transcriptionally nor epigenomically and simply halt in their developmental progression. This breakdown in lineage maturation is also evident during live-cell tracking in *Mesp1* KO embryos: *Mesp1* lineage cells exit the primitive streak at the same time as controls, are capable of migratory behaviors, but lack the crucial anterior-lateral directionality to their movements that is required for convergence at the anterior midline (Dominguez et al., 2023). Ultimately, although *Mesp1* KO embryos specify early cardiac lineage cell types upon exiting pluripotency, their deficient regulatory landscapes coincide with failed anterior migration and result in halted maturation of the cardiac lineage (Fig. 8).

Positing *Mesp1* as a master transcriptional regulator of early cardiac fate is largely informed by overexpression studies (Chiapparo et al., 2016; Bondue et al., 2008; Lindsley et al., 2008; Lin et al., 2022), in contrast to earlier *in vivo* studies that suggested a *Mesp1*-dependent role for cardiac mesoderm migration (Saga et al., 2000). Indeed, we observed downregulation of migratory genes in *Mesp1* KO embryos, and a companion work demonstrates that *Mesp1*-dependent migration patterns are required for spatial organization of CPCs during cardiogenesis (Dominguez et al., 2023). Additional interpretations in *Mesp1/Mesp2* double knockouts underscore the potential for more complex networks of TF dependency in cardiac specification that are not fully explained by regulatory hierarchies (Ajima et al., 2021; Kitajima et al., 2000; Saga, 1998). Although the concept of a master TF is a broadly applied framework for interrogation of gene regulatory networks (Cai et al., 2020; Davis and Rebay, 2017; Yin and Wang, 2014), this likely oversimplifies cardiogenesis. Indeed, our high-resolution, single-cell transcriptional and epigenomic analyses reveal both transcriptional resilience and vulnerability of early cardiogenesis in a regulatory landscape lacking *Mesp1*.

Transcriptional profiling of *Smarcd3*-F6^+^ cells highlighted that *Mesp1* KO cells were mostly represented by transcriptional signatures of cell types from early cardiogenesis, indicating that *Mesp1* KO CPCs initiate and progress through early stages of cardiac specification. This finding contrasts with the *Mesp1*-dependent failure to exit pluripotency that was previously highlighted (Lescroart et al., 2018). We interpret the failed induction of *Nkx2-5*, which is crucial for patterning of the first and second heart fields (Harvey, 2002), and the inappropriate levels of Gata factors in *Mesp1* KO CPCs as representing a breakpoint between specification and maturation phases of the cardiogenic process.

To characterize this breakpoint, we utilized complementary scATAC-seq and scRNA-seq mesoderm datasets to conclude that mesendoderm regulatory programs, instructed at least partially by *Eomes*, are responsible for the initiation of and progression through middle phases of cardiac specification prior to cardiac crescent formation. However, the perdurance of these programs, coupled with the failure of LPM to properly migrate anterior-laterally in *Mesp1* KO embryos, leads to aberrant upregulation and ectopic expression of early cardiac mesoderm, non-cardiac mesoderm, and mesendoderm genes and TFs. Additionally, we hypothesize that the posterior positioning of CPCs in *Mesp1* KO embryos further compounds cardiac maturation defects and CPC transcriptional profiles via improper exposure to signaling gradients and growth factors. The dysregulated identity of *Mesp1* KO cardiac mesoderm in this phase between middle and late embryonic stages stalls *Mesp1*-deficient cardiogenesis due to failed induction of cardiac progenitor patterning, morphogenesis, and CM maturation regulatory programs.

Although this developmental breakpoint is observed between E7.5 and E7.75, well after transient *Mesp1* expression has declined, these processes appear to be *Mesp1* dependent. Possible explanations for this phenomenon are: (1) improper repression of earlier regulators, such as *Eomes*; (2) compounded, *Mesp1*-dependent secondary effects influencing de-repression or ectopic activation; or (3) exposure of *Mesp1* KO CPCs to improper embryonic signaling cues as a result of their aberrant posterior localization and migration. Future studies with additional genetic models and analysis of earlier developmental time points are needed to disentangle these possibilities.

Overall, our work shows that complex transcriptional networks and interdependent hierarchies govern CPC emergence and differentiation. We characterize an initial, transcriptionally resilient phase of CPC specification and identify that the epigenomic landscape necessary for CPCs to transition from LPM to CPs and CMs is dependent on effects of preceding *Mesp1* activity. Our results point to generalizable transcriptional regulatory principles during gastrulation for the allocation of precursor cells from embryonic germ layers towards restricted fates, and differentiation to distinct functional cell types.

## MATERIALS AND METHODS

### Mouse models

Animal studies were performed in strict compliance with the UCSF Institutional Animal Care and Use Committee. Mice were housed in a standard 12 h light/dark animal husbandry barrier facility at the Gladstone Institutes. The *Mesp1^Cre/+^* knock-in mice were the original strain obtained from Yumiko Saga (Saga et al., 1999), which includes the PGK-Neo cassette. *Rosa26R^Ai14^* mice were from the Jackson Laboratory (strain #007914; Madisen et al., 2010). Tdgf1::*lacZ* mice containing a transgene for *Tdgf1* enhancer with a *lacZ* reporter were obtained from Brian Black (Barnes et al., 2016).

Control embryos were generated from crosses of *Mesp1^Cre/+^;Rosa26R^Ai14^;Hipp11^Smarcd3-F6::eGFP^* males to C57BL/6J wild-type, *Mesp1^Cre/+^* or *Mesp1^Cre/+^;Rosa26R^Ai14^;Hipp11^Smarcd3-F6::eGFP^* females. *Mesp1* KO embryos were generated from crosses of *Mesp1^Cre/+^;Rosa26R^Ai14^;Hipp11^Smarcd3-F6::eGFP^* males to *Mesp1^Cre/+^* or *Mesp1^Cre/+^;Rosa26R^Ai14^;Hipp11^Smarcd3-F6::eGFP^* females. Transgenic embryos for single-cell transcriptomic and epigenomic sequencing experiments were all on a C57BL/6J background. Transgenic embryos for whole-mount *in situ* hybridizations and immunohistochemistry validations were on C57BL/6J backgrounds or a mixed CD1/C57BL/6J background in order to facilitate better littermate stage matching via larger litters, with litters born to *Mesp1^Cre/+^* CD1/C57BL/6J hybrid females mated to *Mesp1^Cre/+^;Rosa26R^Ai14^;Hipp11^Smarcd3-F6::eGFP^* C57BL/6J males. ‘Control’ denotes embryos with at least one wild-type allele in the *Mesp1* locus and includes genotypes *Mesp1^Cre/+^;Rosa26R^Ai14^;Hipp11^Smarcd3-F6::eGFP^*, *Mesp1^Cre/+^;Rosa26R^Ai14/+^ ;Hipp11^Smarcd3-F6::eGFP/+^*, *Mesp1^+/+^;Rosa26R^Ai14^;Hipp11^Smarcd3-F6::eGFP^*, and *Mesp1^+/+^;Rosa26R^Ai14/+^ Hipp11^Smarcd3-F6::eGFP/+^.* Heterozygosity of *Mesp1^Cre/+^* or *Mesp1^+/+^* is noted when control embryos were utilized in scRNA-seq (Fig. S1) or scATAC-seq (Fig. S11) library generation. ‘*Mesp1* KO’ denotes embryos with homozygosity of the Cre insertion disrupting the *Mesp1* locus and includes genotypes *Mesp1^Cre/Cre^;Rosa26R^Ai14^;Hipp11^Smarcd3-F6::eGFP^* and *Mesp1^Cre/Cre^;Rosa26R^Ai14/+^;Hipp11^Smarcd3-F6::eGFP/+^*.

Control embryos for activity assessment of the *Tdgf1* enhancer had genotypes *Mesp1^Cre/+^;Tdgf1*::*lacZ* or *Tdgf1*::*lacZ*, and *Mesp1* KO embryos had the genotype *Mesp1^Cre/Cre^;Tdgf1*::*lacZ*.

### Cloning and generation of TARGATT transgenic knock-in mice

The *Smarcd3*-F6 fragment was isolated and cloned with inclusion of an *nlsEGFP* under control of an *Hsp68* minimal promoter for TARGATT (Applied Stem Cells) insertion to the *Hipp11* locus as previously described (Devine et al., 2014) to create the *Hipp11^Smarcd3-F6::eGFP^* mouse (Devine et al., 2014). Purified construct DNA was injected into embryo pronuclei along with mRNA for the *Phi31o* transposase according to manufacturer's protocols.

### Timed matings and whole embryo dissections

To achieve timed matings, male and female mice were housed together in the evening and pregnancy was assessed by vaginal plug the following morning. Gestational stage was determined relative to noon on the day of plug detection, defined as day E0.5. Females were confirmed pregnant by abdominal ultrasound (Vevo 3100, Visual Sonics) the afternoon of day 6 or morning of day 7 and sacrificed according to IACUC standard procedure at noon on day 7, or the early morning of day 8. The embryonic ages captured in individual litters ranged from E6.0 to E7.5 on day 7, and E7.5 to E7.75 on day 8. The diversity of ages in litters aided in the construction of a fine time course for both mutant and control timelines.

Embryos were dissected and, at later stages when yolk was present, also de-yolked, in ice-cold PBS (Life Technologies, 14190250) with 1% fetal bovine serum (FBS; Thermo Fisher Scientific, 10439016) on ice. Embryos were screened using an upright epifluorescence dissecting microscope (Leica MZFLIII microscope, Lumen Dynamics XCite 120LED light source, Leica DFC 3000G camera) for presence of both red and green fluorescent reporters, indicative of *Mesp1* lineage tracing from *Mesp1^Cre^;Rosa26R^Ai14^* alleles and expression of the *Smarcd3*-F6::eGFP transgene reporter from the *Hipp11^Smarcd3-F6::eGFP^* allele, respectively. Embryos were staged according to Downs and Davies (1993), at the discretion of experimentalist, and with input from colleagues. For difficult-to-capture control stages used in construction of the wild-type scRNA-seq timeline, absence of the *Mesp1* lineage (Ai14) reporter was permitted and noted for those embryos (Fig. S1). Additionally, *Mesp1^+/+^* alleles were specifically included in addition to *Mesp1^Cre/+^* as controls for scATAC-seq library generation in the event that locus-specific effects of Cre insertion required additional consideration, which we did not find to be the case as both control genotypes appeared identically in the dataset (Fig. S11). DNA for genotyping was extracted using QuickExtract DNA Extraction Solution (Lucigen, QE09050) from harvested yolk sac tissue if available or else from a micro-dissected nick of the extra-embryonic anterior proximal region. Genotyping was performed to distinguish *Mesp1* KO embryos from control embryos using Phire Green Hot Start II DNA Polymerase (Thermo Fisher Scientific, F124L) according to manufacturer's protocols using primers to detect wild-type bands (control, P1+P3) and Cre alleles (*Mesp1* KO, P1+P2): *Mesp1* FWD, P1: GGCCATAGGTGCCTGACTTA; Cre2 REV, P2: CCTGTTTTGCACGTTCACGG; *Mesp1* REV, P3: ACCAGCGGGA-CTCAGGAT.

### Embryo preparation for single-cell library generation

Owing to the small size and lack of morphological distinction between tissue types of embryos at these early stages, whole embryos were dissected and harvested for single-cell library generation.

Whole embryos were incubated in 200 μl 0.25% TrypLE (Thermo Fisher Scientific, 12563029) solution for 5 min at 37°C and triturated gently. The dissociated cell suspension was quenched with 600 μl of PBS with 1% FBS, singularized by passage through a 70 μm cell strainer (BD Falcon, 352235), pelleted by centrifugation at 150 ***g*** for 3 min, and resuspended in 34 μl of PBS with 1% FBS. At least two embryos were collected per genotype per embryonic stage in all datasets except for the *Mesp1* KO embryos in the scRNA-seq dataset where this was not possible, and the use of relative developmental stages was employed in analysis along with replicate validations by *in situ* hybridization for differentially expressed genes.

### Single-cell transcriptome library preparation and sequencing

Libraries for scRNA-seq were prepared according to manufacturer's instructions using the 10x Genomics Chromium controller, Chromium Single Cell 5′ Library and Gel Bead Kit v1 (10x Genomics, 1000006) and Chromium Single Cell A Chip Kit (10x Genomics, 1000151). A targeted maximum recovery of 10,000 cells per sample were loaded onto the 10x Genomics Chromium instrument, and each sample was indexed with a unique sample identifier (10x Genomics Chromium i7 Multiplex Kit, 120262). Final libraries were pooled and sequenced shallowly according to 10x protocol parameters on a NextSeq500 (Illumina), and then re-pooled for deeper sequencing on HighSeq4000 (Illumina) and/or NovaSeq using an S4 lane (Illumina). Littermate, stage-matched comparisons of control and *Mesp1* KO libraries were always sequenced together in the same library pool. All scRNA-seq libraries were sequenced to a mean read depth of at least 50,000 total aligned reads per cell.

### Processing raw scRNA-seq data

Raw sequencing reads were processed using the 10x Genomics Cell Ranger v3.0.2 pipeline. Reads were demultiplexed using cellranger mkfastq and aligned with cellranger count to the Mm10 reference genome containing additional sequences for Ai14 and eGFP. Cellranger aggr ‘aggr’ was used to aggregate and read depth normalize multiple GEM libraries for either the WT atlas dataset or the atlas dataset containing control and *Mesp1* KO embryo libraries.

### Seurat analysis of scRNA-seq data

Outputs from the Cell Ranger pipeline were analyzed using the Seurat Package v3.0.2 in R (Butler et al., 2018; Stuart et al., 2019; Satija et al., 2015). The dataset containing all wild-type embryos and the ‘WTvsMut’ dataset containing control (WT) and *Mesp1* KO embryos were analyzed as separate Seurat objects. A single aggregated counts matrix for each separate dataset were used as inputs for Read10X and CreateSeuratObject functions. Quality control steps were performed to remove dead cells or doublets.

#### WT atlas

For the WT atlas, cells with <10% mitochondrial reads, unique molecular identifier (UMI) counts less than 50,000, and detected genes between 200 and 6300 were retained. SCTransform (Hafemeister and Satija, 2019) was used to normalize and scale data with regressions performed with respect to mitochondrial percentage, number of genes, and number of UMI counts detected. Principal components analysis (PCA) and batch correction were performed using FastMNN (Haghverdi et al., 2018) split by experimental group (experiment number denoted with library prefixes ALK06, ALK08, ALK07, ALK05, ALK04); 94,824 cells were clustered based on the top 50 principal components and visualized using RunUMAP, FindNeighbors, and FindClusters and outputs were visualized as UMAP embeddings generated with DimPlot. Cell types were annotated at clustering resolution 0.4 using the FindAllMarkers function with Wilcoxon rank-sum test (min.pct=0.1, logfc threshold=0.25) to identify cluster-specific marker genes. Relevant mesoderm cell types were subsetted based on cluster-wise detection of *Smarcd3*-F6::eGFP and Ai14 transgenes for CPCs and the *Mesp1* lineage, respectively. The resulting 34,724 were re-clustered and re-annotated at resolution 1.2 to create the cardiac mesoderm WT atlas.

#### Whole-embryo control versus *Mesp1* KO atlas

For the WTvsMut atlas, cells with <10% mitochondrial reads, UMI counts <50,000, and detected genes between 200 and 7000 were retained. SCTransform was used to normalize and scale data with regressions performed with respect to mitochondrial percentage, number of genes, and number of UMI counts detected. PCA and batch correction were performed using FastMNN split by experimental group as in the WT dataset. Cells were clustered as described for the WT atlas above, with iterative clustering performed following removal of low-quality clusters. This WTvsMut dataset represents 96,027 cells containing 79,725 control and 16,302 *Mesp1* KO cells. Cluster cell types were annotated at resolution 1.0 using FindAllMarkers as described above.

The relevant developmental stages were annotated within Seurat meta data. Cells from six embryos staged E6.0-E6.5 (ALK06_2_E60_con_rep1, ALK06_4_E60_con_rep2, ALK08_20_E60_con_rep3, ALK08_14_ lateE60_con_rep1, ALK07_15_E65_con_rep1, ALK08_6_E65_Mesp1KO_rep1) were denoted as ‘early’ stages. Cells from four embryos staged late E6.5 to early E7.5 (ALK07_3_lateE65_con_rep1, ALK07_14_E70_ con_rep1, ALK08_11_E70_Mesp1KO_rep1, ALK07_7_earlyE75_con_ rep1) were denoted as ‘middle’ stages. Cells from five embryos staged late E7.5 to early E7.75 when the cardiac crescent is formed (ALK07_6_ lateE75_con_rep1, ALK04_3_lateE75_con_rep2, ALK05_7_E775_con_ rep1, ALK05_2_lateE75_Mesp1KO_rep1, ALK07_8_E775_con_rep2) were denoted as ‘late’ stages. Although we set out to acquire replicates of both genotypes per each stage as the most optimal statistical scenario, the 25% yield of *Mesp1* KO embryos within C57BL/6J litter sizes at these early gastrulation stages proved prohibitive. Thus, we relied on validations of key scRNA-seq findings via the orthogonal approach of multiplexed whole-mount *in situ* hybridizations.

#### *Smarcd3*-F6^+^ control versus *Mesp1* KO atlas

To analyze putative CPCs, all cells expressing the *Smarcd3-*F6-eGFP transgene were subsetted from the full WTvsMut atlas and re-clustered into their own Seurat object containing 4868 cells (4276 control and 592 *Mesp1* KO cells). The FindAllMarkers function was used to identify cluster marker genes of represented cell types at resolution 1.7. Analysis of control and *Mesp1* KO genotypes irrespective of cell type was performed using FindMarkers function between genotypes with Wilcoxon rank-sum test (min.pct=0.1, logfc threshold=0.25). Cluster-wise differential gene expression testing was performed using the FindMarkers function and Wilcoxon rank-sum test (min.pct=0.1, logfc threshold=0.25) between genotypes within specific cell-type clusters, and visualized with the VlnPlot function. Differential gene expression results irrespective of cell type were visualized using the DotPlot function separated by genotypes and also genotypes separated by developmental stages.

#### Mesoderm control versus *Mesp1* KO atlas

Relevant mesoderm cells were subsetted from the full WTvsMut atlas based on cluster-wise detection via FeaturePlot and VlnPlot at cluster resolution 1.0 of *Smarcd3*-F6::eGFP and Ai14 transgenes for CPCs and the *Mesp1* lineage, respectively. The resulting 35,792 cells (29,924 control and 5868 *Mesp1* KO cells) of the WTvsMut mesoderm dataset was re-clustered and annotated at resolution 1.5 using the FindAllMarkers function as above to identify cell-type marker genes as described above.

Embryos representing the relative developmental stages of ‘early’ (5504 cells; 4472 control and 1032 *Mesp1* KO), ‘middle’ (7666 cells; 6734 control and 932 *Mesp1* KO) and ‘late’ (22,622 cells; 18,718 control and 3904 *Mesp1* KO) as described above were subsetted into respective individual Seurat objects, re-clustered as described, and cell-type clusters were further re-annotated (early, middle and late objects all at resolution 0.7). Clusters representing cell types relevant for cardiac development were identified through cluster-wise enrichment of *Smarcd3*-F6::eGFP transgene expression overlayed in UMAP space via FeaturePlot. Differential gene expression testing between genotypes within cardiogenic cell-type clusters was performed using the FindMarkers function with Wilcoxon rank-sum test (min.pct=0.1, logfc threshold=0.25). Differentially expressed genes with adjusted *P*-values <0.05 were plotted as violin plots in Seurat except for cases in which we wished to highlight the total absence of transcript in one genotype condition.

#### Whole-embryo control versus *Mesp1* KO atlas for scATAC-seq integration

For the scRNA-seq WTvsMut atlas for integration with scATAC-seq data, libraries from middle-stage embryos (ALK07_3_lateE65_con_rep1, ALK07_14_E70_con_rep1, ALK08_11_E70_Mesp1KO_rep1, ALK07_7_earlyE75_con_rep1) and late-stage embryos (ALK07_6_lateE75_con_rep1, ALK04_3_lateE75_con_rep2, ALK05_7_E775_con_rep1, ALK05_2_lateE75_Mesp1KO_rep1, ALK07_8_E775_con_rep2) were subsetted from the aggregated WTvsMut counts matrix. Cells with <7.5% mitochondrial reads, UMI counts <50,000, and detected genes between 200 and 7000 were retained. SCTransform was used to normalize and scale data with regressions performed with respect to mitochondrial percentage, number of genes, and number of UMI counts detected. PCA and batch correction were performed using FastMNN split by experimental group. After initial clustering as previously described, cell clusters representing low-quality cells were removed and clustering was iterated again. The resulting dataset represents 82,536 cells containing 68,717 control and 13,819 *Mesp1* KO cells. Cluster cell types were annotated at resolution 1.2 using FindAllMarkers as described above.

#### Mesoderm control versus *Mesp1* KO atlas for scATAC-seq integration

Relevant mesoderm cells were subsetted from the whole-embryo matched scATAC-seq WTvsMut atlas based on cluster-wise detection via FeaturePlot and VlnPlot of *Smarcd3*-F6::eGFP and Ai14 transgenes for CPCs and the *Mesp1* lineage, respectively. The resulting 30,427 cells (26,054 control and 4373 *Mesp1* KO cells) of the scATAC-seq matched mesoderm WTvsMut dataset were re-processed from RNA assay slot with the standard Seurat workflow NormalizeData, FindVariableFeatures, and ScaleData. SCTransform was not used in this mesoderm scRNA-seq dataset because we found that, although cell type label-transfer with scATACseq was successful as previously described for the whole-embryo integration, downstream scATAC-seq analyses leveraging the scRNA-seq gene integration matrix performed in the mesoderm scATAC-seq dataset were incompatible with SCT-normalized values. PCA and batch correction were performed using FastMNN split by experimental group. From here, clustering was performed as previously described and cell types were annotated at resolution 1.2 using the FindAllMarkers function as above to identify cell-type marker genes as described above.

Differential gene expression testing between genotypes within cell-type clusters and between cell-type clusters was performed using the FindMarkers function with Wilcoxon rank-sum test (min.pct=0.1, logfc threshold=0.25). These lists of differentially expressed genes served as inputs to the (peak, gene) association analyses with scATAC-seq differential peaks using rGreat (see ‘Association between scATAC-seq differential peaks and scRNA-seq differentially expressed genes’ section in Materials and Methods).

#### Cell type annotations

Cell-type annotations were named within the context of each dataset within the UMAP presented. Naming of cell types within a dataset was determined from differentiating gene expression profiles of clusters relative to other clusters within that dataset. When multiple clusters presented similar gene expression profiles denoting similar cells, clusters were annotated with the cell type with the addition of a further identifying suffix to enable cross-referencing with corresponding gene expression lists presented in the dataset-associated supplementary tables (Tables S1-S6). In circumstances where annotations could not be confidently derived from cluster gene expression profiles, clusters were named with a ‘C’ prefix and their UMAP resolution-relative cluster number.

### Single-cell transcriptomic cell trajectories and pseudotime analysis

Pseudotime analysis was performed using the URD package (version 1.0.2 and 1.1.1) (Farrell et al., 2018). The WTvsMut mesoderm Seurat object containing all three relative developmental stages, processed as described in ‘Mesoderm control versus *Mesp1* KO atlas’ section of Materials and Methods, was converted to an URD object using the seuratToURD function. Cell-to-cell transition probabilities were constructed by setting the number of near neighbors (knn) to 189 and sigma to 10. Pseudotime was then calculated by running 80 flood simulations with *Pou5f1*^+^ epiblast cells containing ‘early’ staged embryos (cluster 1 of WTvsMut mesoderm Seurat object at resolution 1.5 as shown in Fig. S6) as the ‘root’ cells. Clusters containing the most defined mesodermal derivative cell types and containing the ‘late’ staged embryos were set as the ‘tip’ cells (C15-, C16-HPCs, C11-, C7-Endothelial, C20-CFMeso, C2-Allantois, C12-CMs, C29-CardiacMeso, C0-postLPM, C22, C26-LPM, C14-PrSoM-like, C4-postPrxM1, C18-Meso). The resulting URD tree was subsequently built by simulated random walks from each tip. Overlay of relative developmental stages from embryo data was used to show consensus in pseudotime estimations of cell trajectories. Overlay of *Smarcd3*-F6::eGFP and various cardiac marker genes, such as *Nkx2-5*, *Myl7*, *Smarcd3*, *Tnnt2*, and various *Gata* transcription factors, were used to identify the relevant cardiac-fated branching segments of the URD tree.

To identify differentially expressed genes in fate-related cells of the cardiac branches, cell barcodes from relevant branch segments were extracted from the URD object and assigned their relevant segment branch identities in the corresponding Seurat object. Differential gene testing using the Wilcoxon rank sum test (min.pct=0.1, logfc threshold=0.25) was then performed between genotypes within a segment or between noted segments related in their pseudotemporal progression. Differentially expressed genes with adjusted *P*-values <0.05 were plotted as violin plots in Seurat and representative genes were overlayed on the URD tree to visualize expression patterns in pseudotime space.

### scATAC-seq library generation

For scATACseq library generation, we used the 10x Genomics Chromium, scATACseq library kit v1 (10x Genomics, 1000110) and Chromium Chip E (10x Genomics, 1000156) according to the manufacturer's protocols. Embryos were dissected and dissociated into single cells as described above and cells were resuspended in pre-chilled lysis buffer for isolation of single nuclei. In our hands, the nuclear isolation step was technically challenging to perform on embryos <E7.5 owing to the low number of starting cells within these small embryos resulting in too few harvested nuclei to proceed with generation of a library. This low-nucleus yield from small amounts of starting cells represents a technical hurdle we could not overcome in this study and thus we were unable to include young embryonic stages in our scATAC-seq dataset. A maximum targeted recovery of 10,000 nuclei per sample were subjected to transposition and loaded into the 10x Genomics Chromium instrument. Final libraries were pooled and sequenced shallowly according to 10x Genomics protocol parameters on a NextSeq500 (Illumina). Littermate, stage-matched comparisons comprising a total of five control and four *Mesp1* KO embryos were ultimately re-pooled and sequenced together for deep sequencing on a NovaSeq6000 S4 lane (Illumina). All libraries were sequenced to depths of at least 24,000 median fragments per cell, and at most 35,000 median fragments per cell.

### Processing raw scATAC-seq

Raw sequencing reads were processed using the 10x Genomics Cell Ranger v1.2.0 software pipeline. Reads were demultiplexed using cellranger atac mkfastq. Cell barcodes were filtered and aligned to the Mm10 reference genome using cellranger atac count. The resulting output indexed fragment files from each library were not aggregated and served as the inputs for downstream computational analysis in ArchR (Granja et al., 2021).

### ArchR analysis of scATAC-seq

Downstream computational analysis of scATAC-seq data was performed with the ArchR software package v1.0.1 in R (Granja et al., 2021). Initial Arrow files were generated for all samples from inputs of respective indexed fragment files and sample meta-data. Samples from embryos aged E7.5 were called ‘middle’ stage (libraries ALK10_5_E75_con_rep1, ALK10_3_ E75_con_rep2, ALK10_1_lateE75_con_rep1, ALK10_7_E75_Mesp1 KO_rep1, ALK10_2_E75_Mesp1KO_rep2). Samples from embryos aged E7.75 were called ‘late’ stage (libraries ALK09_3_E775_con_rep1, ALK09_2_E775_con_rep2, ALK09_1_E775_Mesp1KO_rep1, ALK10_ 6_E775_Mesp1KO_rep2). The function createArrowFiles was run on each sample, removing cells with a TSS enrichment score <4 and fragments <5000. This initialization also creates a genome-wide TileMatrix of 500 base pair bins and a weighted calculation of accessibility within and surrounding gene loci annotated from the Mm10 genome, called a GeneScoreMatrix. Whereas Cell Ranger v1.2.0 implements removal of multi-cell capture, ArchR recommends an additional round of cell doublet removal using functions addDoubletScores and filterDoublets. Individual ArrowFiles for each sample were aggregated into a single WTvsMut whole embryo ArchRProject containing 46,819 cells (26,295 control, 20,524 *Mesp1* KO) with a median TSS enrichment score of 10.675 and median of 30,703 fragments per cell. Dimensionality reduction was performed with addIterativeLSI (two iterations, resolution 0.2, 30 dimensions). Clustering was performed using addClusters with the ‘Seurat’ method (resolution 0.8) and addUMAP was used to embed values for dimensionality reduced visualizations with the function plotEmbedding. Relative cell-type annotation of clusters was performed with consideration of combined information from GeneScore plots and label transfer from the complementary annotated whole embryo WTvsMut scRNA-seq Seurat analysis object of stage-matched control and *Mesp1* KO embryos for the relative middle (embryos ALK07_3_lateE65_con_rep1, ALK07_14_E70_con_ rep1, ALK08_11_E70_Mesp1KO_rep1, ALK07_7_earlyE75_con_rep1) and late (embryos ALK07_6_lateE75_con_rep1, ALK04_3_lateE75_con_rep2, ALK05_7_E775_con_rep1, ALK05_2_lateE75_Mesp1KO_rep1, ALK07_8_E775_con_rep2) stages. For scRNA-seq integration, the addGeneIntegrationMatrix function utilizes Seurat's FindTransferAnchors to perform canonical correlation analysis. Relevant mesoderm clusters (C15, C9, C24, C17, C16, C18, C12, C11, C8) were identified based on relative overlay of scRNA-seq cell type labels onto scATAC-seq clusters and GeneScoreMatrix for key marker genes, and subsetted into a WTvsMut mesoderm ArchRProject containing 25,848 cells (14,212 control and 11,636 *Mesp1* KO).

Dimensionality reduction was performed on the subsetted WTvsMut mesoderm ArchRProject with addIterativeLSI (four iterations, resolution 0.2, 30 dimensions), which was then batch corrected using addHarmony. Harmonized clustering was then performed using addClusters with the ‘Seurat’ method (resolution 0.8) and addUMAP was performed. Clusters were visualized using plotEmbedding. Relative cell-type annotation of clusters was again performed following integration with the mesoderm WTvsMut complementary, annotated, Seurat analysis scRNA-seq object from stage-matched control and *Mesp1* KO embryos for the relative middle and late stages. The addGeneIntegrationMatrix function was used to generate GeneIntegration plots, which were compared with GeneScore plots for understanding of cluster markers. A Jaccard Similarity Analysis from the predicted scRNA-seq integration for scATAC-seq clusters annotation was performed similarly to as described (Sarropoulos et al., 2021) to assess the strength of predictive labels, and the resulting proportions were visualized with the pheatmap function from the ComplexHeatmap R package (Gu et al., 2016). Cluster identities from the mesoderm subset scATAC-seq dimensionality reduction were utilized for downstream cluster-wise analyses.

#### Peak calling and motif enrichment

Peaks were called using pseudo-bulkification and MACS2. Cell replicates for pseudobulks were created using addGroupCoverages on scATAC-seq clusters (40 minimum and 500 maximum cells in a replicate, minimum two replicates per cluster, 0.8 sampling ratio, kmerlength for Tn5 bias correction of 6). Peaks were called using addReproduciblePeakSet (500 peaks per cell, 1.5E5 maximum peaks per cluster) with MACS2 (−75 base pair shift per Tn5 insertion, 150 base pair extension after shift, excluding mitochondrial chromosome genes and chromosome Y genes, with a q-value significance cutoff 0.1). Peaks were then merged using ArchR's iterative overlap method. Cluster-enriched marker peaks were identified with getMarkerFeatures (FDR≤0.05, log2FC≥1) and visualized with plotMarkerHeatmap. Cluster motif enrichment was ascertained with addMotifAnnotations using the CIS-BP database motif set. Cluster enriched motifs were visualized with peakAnnoEnrichment (FDR≤0.05, log2FC≥1) and then the top seven motifs per cluster were plotted with plotEnrichHeatmap and ComplexHeatmap. Single-cell resolution motif enrichment was computed using the chromVAR package (Schep et al., 2017) by adding background peaks (addBgdPeaks) and then motif z-score deviations were computed per cell with addDeviationsMatrix. Motif enrichments were visualized in UMAP embeddings with plotEmbedding.

#### Pseudotime ordering of cardiogenic trajectory

A pseudotime trajectory approximating the differentiation of progenitor cell types to mature cell types was curated using the addTrajectory function (preFilterQuantile=0.9, postFilterQuantile=0.9) to order cells along the trajectory backbone C6, C5, C12, C13, C7, C8, C14. This backbone represents the biologically relevant cardiogenic differentiation path: epiblast, EomesME, Mesp1ME, LPM2, LPM1, CMs/CPs. We leveraged ArchR's series of pseudotime vector calculations to fit and align individual cells based on their Euclidean distances to the defined backbone's cell-type clusters' mean coordinates in order to fit a continuous trajectory path in batch corrected LSI dimensional space. This resulting path with scaled, per-cell pseudotime values was then visualized in UMAP space using the plotTrajectory function. We then performed an integrative analysis to identify positive TF regulators along trajectory pseudotime. We integrated gene accessibility scores and gene expression data with motif accessibility across pseudotime using the correlateTrajectories function and visualized correlated matrices in trajectory space with the plotTrajectoryHeatmap function.

#### Assessment of positive TF regulators

A putative positive regulator represents a TF for which gene expression is positively correlated to changes in accessibility of its corresponding motifs. Using the previously calculated motif z-score deviations, we stratified motif z-scores variation between all clusters to identify the maximum motif z-score delta. We next used the correlateMatrices function to correlate motifs to gene expression in batch-corrected LSI dimensional space, then used these correlations to identify motifs with maximized deviance from expected accessibility averages in other cells, and ranked TFs accordingly. We required positive TF regulators to have correlations >0.5 (and adjusted *P*-value<0.01) between their gene expression and corresponding motifs, and deviation z-scores with maximum inter-cluster variation difference in the top quartile (quantile 0.75). Correlations were plotted for visualization using the ggplot function. Although the ranking association with analysis might be vulnerable to generating false negatives, wherein potential TF drivers are not recognized, we found overlay of motifs with TF gene expression and gene score values along the cardiogenic trajectory and in UMAP cluster space served to sufficiently identify the highest confidence drivers.

#### Differential peaks and differential motif enrichment comparisons between cell types

Pairwise comparisons between cell types of accessible peak differences was performed using the getMarkerFeatures function [Wilcoxon test, TSS enrichment and log10(nFrags) bias, 100 nearby cells for biased-matched background, 0.8 buffer ratio, 500 maximum cells] by setting one cell type as the lead comparison (useGroup) and one cell type as the relative comparison (bgdGroup). These pairwise comparisons of differential peaks were saved as .RDS objects and served as inputs to the (peak,gene) association analyses with rGreat (see ‘Association between scATAC-seq differential peaks and scRNA-seq differentially expressed genes’ section of Materials and Methods). Differentially enriched peaks [FDR≤0.05, abs(log2FC)≥1] were visualized as MA plots. Motif enrichment of differential peaks was determined using the peakAnnoEnrichment function (FDR≤0.05 and log2FC≥1 for useGroup enrichment or else log2FC≤−1 for bgdGroup enrichment) to determine motifs enriched in differential peaks between cell-type groups. Enriched motifs were rank-sorted and colored by significance of enrichment, then plotted using the ggplot function.

#### Assessment of peak-to-gene linkages

Peak-to-gene linkage analysis to assess correlations between chromatin accessibility and gene expression was performed using the addPeak2GeneLinks function on batch-corrected LSI dimensions (correlation cut off>0.45, FDR<1E-4, resolution 1000 bp for optimized browser track visualization). Peak-to-gene linkages for differentially expressed genes (identified in scRNA-seq analyses) were visualized with cell type cluster browser tracks using plotBrowserTrack.

### Association between scATAC-seq differential peaks and scRNA-seq differentially expressed genes

The rGREAT1(v1.26.0) bioconductor R package (https://github.com/jokergoo/rGREAT) was used to generate gene lists linked to scATAC-seq differential peaks based on gene regulatory domains defined as 5 kb upstream, 1 kb downstream of the TSS and up to 100 kb to the nearest gene. The log Fold Change (logFC) for the (peak,gene) pairs where the peak was differentially accessible (FDR≤0.05, log2FC≥1) were plotted to show how the log fold change of the gene expression is associated with the log fold change of the accessibility of peaks. The (peak,gene) pairs in the top-right quadrant (Q3) of the plot correspond to differentially open peaks linked with genes expression of which is upregulated. Similarly, the (peak,gene) pairs in the bottom-left quadrant (Q1) correspond to differentially closed peaks linked with genes expression of which is downregulated. Fisher's test was performed on the counts of (peak,gene) pairs in each of the four quadrants: upregulated genes:differentially open peak regions, downregulated genes:differentially closed peak regions, upregulated genes:differentially closed peak regions and downregulated genes:differentially open peak regions. This provided an estimate of the ratio of the odds of upregulated genes linked to differentially open peak regions versus the odds of upregulated genes linked to differentially closed peak regions.

### Comparative analysis of scATAC-seq with MESP1 ChIP-seq

MESP1 ChIP-seq fastqs from Lin et al. (2022) were downloaded from Gene Expression Omnibus (GSE165102) and processed using nf-core's ChIP-seq pipeline (v1.2.2) (Ewels et al., 2020) with parameters from original authors' methods (Lin et al., 2022). ChIP-seq timepoint replicates were intersected and then time points were merged for subsequent analyses using bedtools (v2.30.0) (Quinlan and Hall, 2010). BED files for scATAC-seq cell type enriched peaks were formatted from associated markerTest PeakSet GRanges objects and cell type bigWigs were generated using ArchR's getGroupBW function (Granja et al., 2021). Overlapping peaks between MESP1 ChIP-seq and cell type-enriched scATAC-seq and MESP1-independent peak regions were determined using bedtools intersect and subtract, respectively (Quinlan and Hall, 2010). TF binding motifs were annotated using Homer (v4.11) findMotifsGenome.pl (Heinz et al., 2010).

### Whole-mount fluorescence *in situ* hybridization experiments

Validation of spatial gene expression and differentially expressed genes was conducted in stage-matched, littermate whole-mount embryos. The assay for whole-mount embryo *in situ* hybridization was adapted from the optimized whole-mount zebrafish embryo protocol using the RNAscope Multiplex Fluorescent Reagent Kit v2 and ProteasePlus (ACDBio) for embryo permeabilization as previously described (Gross-Thebing et al., 2014; de Soysa et al., 2019). De-yolked whole embryos were fixed in 4% paraformaldehyde solution (Electron Microscopy Sciences 15710) overnight at 4°C. Embryos were then washed twice in PBST and processed through 10 min incubations in a dehydration series of 25%, 50%, 75%, 100% methanol on ice. Embryos were stored in 100% methanol at −20°C on a short-term basis until initiation of the *in situ* hybridization protocol. Yolk sac DNA or anterior proximal extra-embryonic regions prior to fixation were used for genotyping. ACDBio RNAscope mouse probes used in this study were: *eGFP* (400281-C1, -C2, -C4), *Tdgf1* (506411-C1), *Fgf8* (313411-C1), *Eomes* (429641-C2), *Myl7* (584271-C3), *Anxa2* (501011-C2), *Nkx2-5* (428241-C2), *Hand1* (429651-C3). Whole-mount embryos were imaged in cold PBS using an upright epifluorescence microscope (Leica MZFLIII, Leica DFC 3000G, Lumen Dynamics XCite 120LED) and acquisition software LASX (Leica). To utilize harvested embryos maximally, we leveraged the ability to multiplex assays by assessing two separate target genes with *Smarcd3*-F6 co-expression in the same embryo. The embryos shown for each pair of panels in Fig. 2H,J, Fig. 2I,K, and Fig. 4I,K represent the same respective embryos multiplexed for multiple targets. Differentially expressed genes were analyzed in at least two replicate embryos per genotype per time point queried. Control and *Mesp1* KO embryo comparisons were imaged and processed with identical parameters.

### Whole-mount embryo X-gal staining and imaging

X-gal staining for *lacZ* enhancer activity was performed according to standard protocols (Anderson et al., 2004; Materna et al., 2018; Sinha et al., 2015; Wilkinson and Nieto, 1993). Briefly, embryos were fixed in 4% paraformaldehyde at 4°C and stored in PBS until initiation of standard X-gal staining protocol. Littermate embryos were processed and imaged identically and simultaneously in brightfield using a Leica MZ165 FC stereomicroscope with DFC450 camera. Genotyping was performed by an operator unaware of the sample groupings. Enhancer activity was assessed in four replicates per genotype.

### Whole-mount embryo immunostaining and light-sheet imaging

Dissected embryos were fixed in 4% paraformaldehyde for 1 h at room temperature with gentle agitation, washed in PBS, and stored in PBS+0.2% sodium azide on a short-term basis at 4°C until initiation of immunostaining. Immunostaining was performed in PCR strip tubes. Embryos were incubated in blocking solution: PBS+5% normal donkey serum, 0.2% sodium azide, 0.5% Triton X-100 (Sigma-Aldrich, X100-500 ml) with 100 μg/ml unconjugated Fab fragment donkey anti-mouse (Jackson ImmunoResearch, 715-007-003) for 2 h at 37°C with gentle rocking agitation. Following PBS washes, primary staining was carried out in blocking solution overnight and subsequently washed with PBS. Secondary staining incubation was carried out in blocking solution for 2-3 h protected from light, and embryos were subjected to final PBS washes. All steps of the immunostaining protocol were performed at 37°C with gentle rocking and rotation. Antibodies used in this study were: sheep polyclonal Foxc2 (R&D Systems, AF6989), chicken polyclonal GFP (Aves Labs, GFP-1020), rabbit polyclonal Cre (Millipore, 69050). Light-sheet embryo images were acquired using a Z1 light sheet microscope (Zeiss) and processed as described (Dominguez et al., 2023).

## Supplementary Material

10.1242/develop.201229_sup1 Supplementary information
