# Supplementary material for: A Mesp1-dependent developmental breakpoint in transcriptional and epigenomic specification of early cardiac precursors
Source: Development. 2023 May 2;150(9):dev201229. doi: 10.1242/dev.201229 (PMC10259516; doi:10.1242/dev.201229)
Supplement: Supplementary information [file develop-150-201229-s1.pdf]

Fig. S1

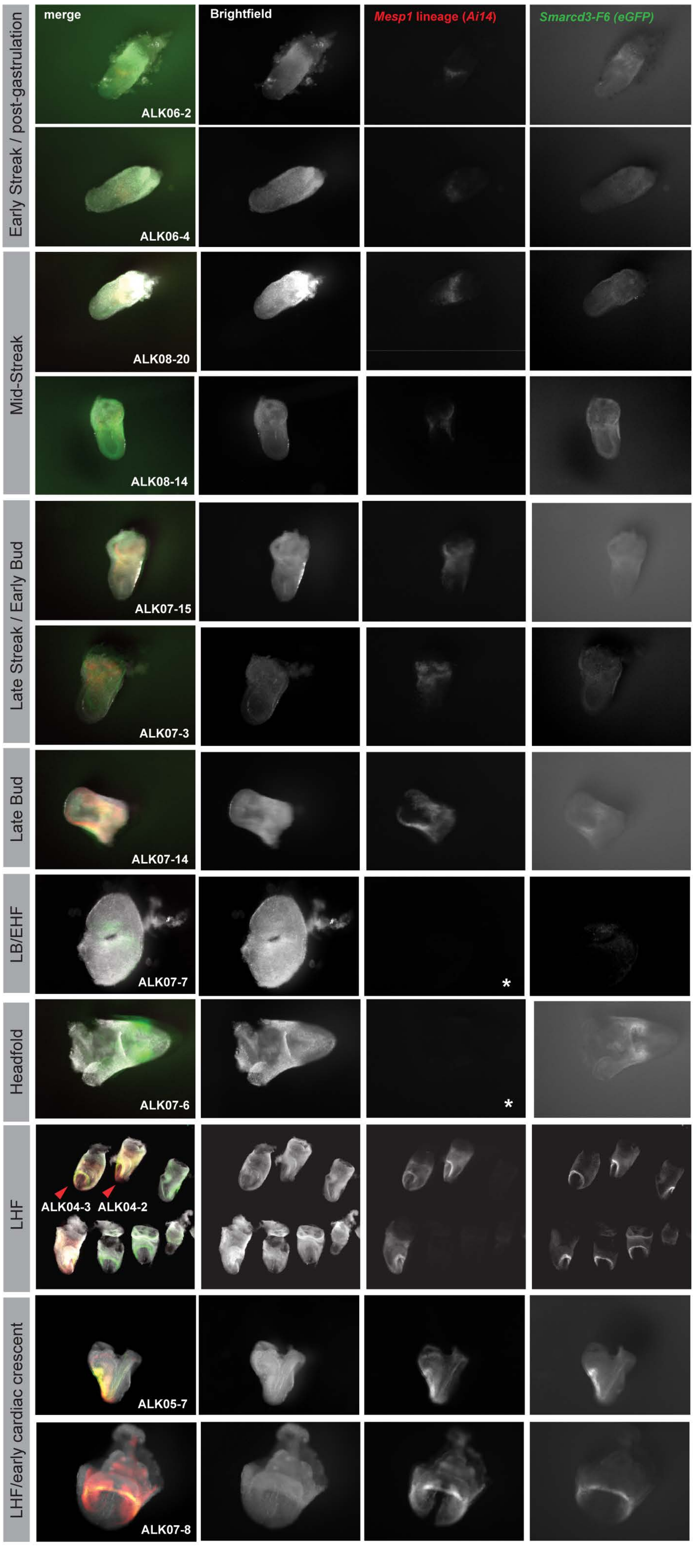

merge
Brightfield
Mesp1 lineage (Ai14)
Smarcd3-F6 (eGFP)
Early Streak / post-gastrulation
Mid-Streak
Late Streak / Early Bud
Late Bud
LB/EHF
Headfold
LHF
LHF/early cardiac crescent
ALK06-2
ALK06-4
ALK08-20
ALK08-14
ALK07-15
ALK07-3
ALK07-14
ALK07-7
ALK07-6
ALK04-3
ALK04-2
ALK05-7
ALK07-8
*
*

Fig. S1. Fluorescent lineage transgenes in whole embryos. Embryos of wildtype gastrulation atlas. *Mesp1* lineage visualized by Ai14 fluorescent reporter transgene. *Smarcd3*-F6 visualized by eGFP fluorescent reporter transgene. Images not acquired and processed identically. Embryos distingushed with * lack Ai14 transgene.

Development • Supplementary information

Fig. S2

A

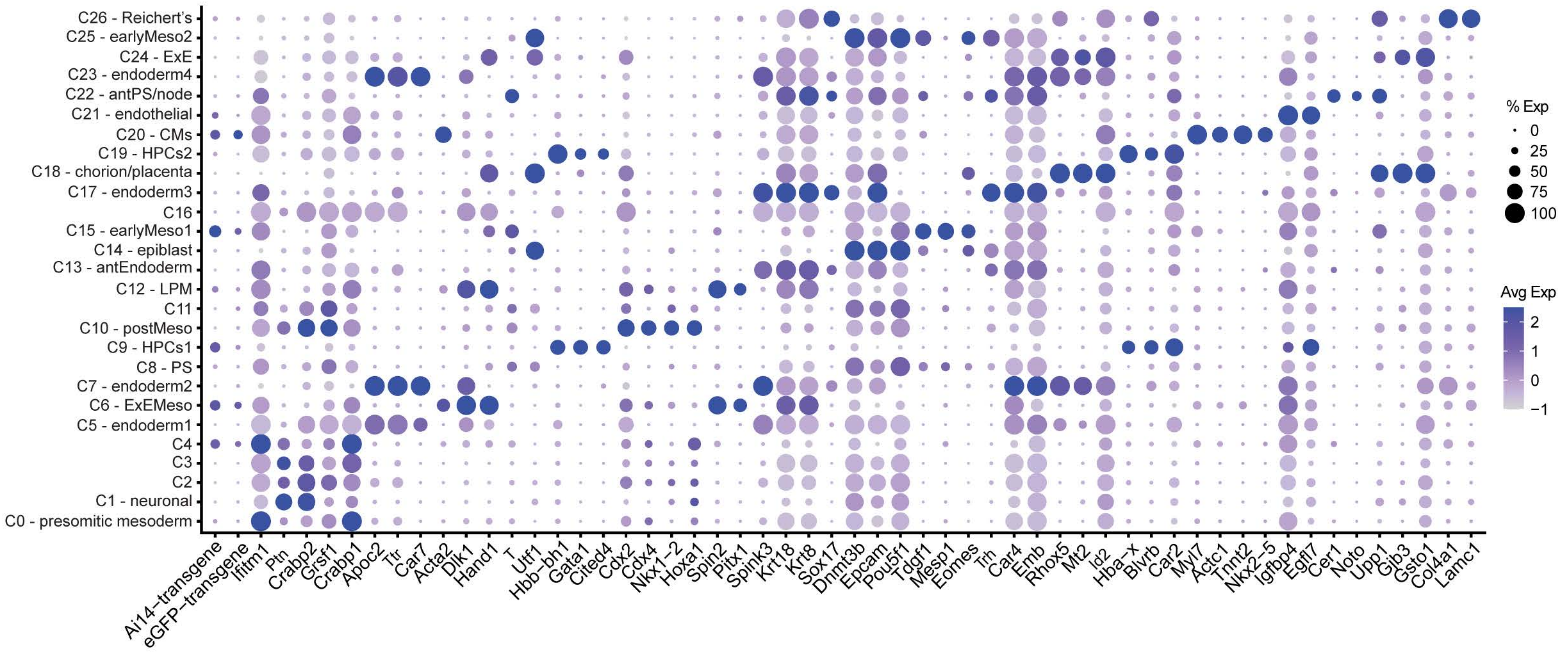

B

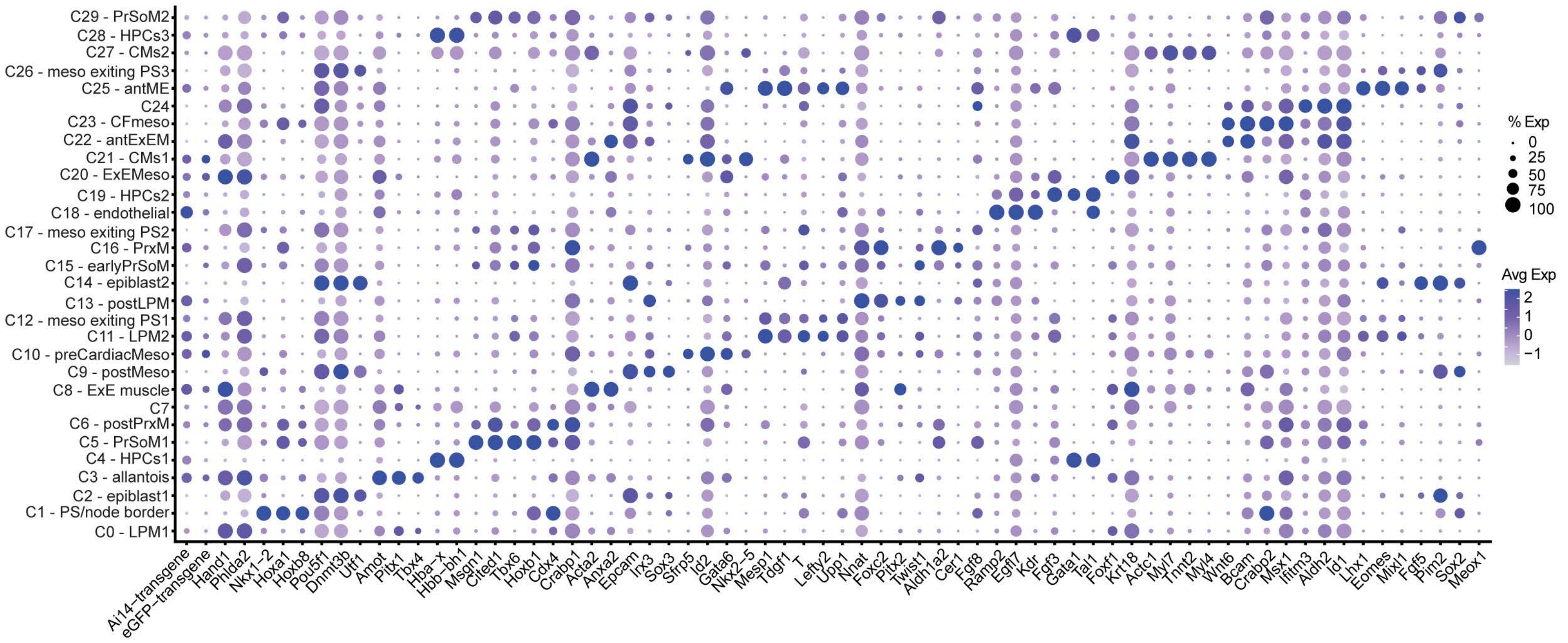

Fig. S2. WT atlases gene expression profiles. (A) Full Embryo WT atlas marker gene dotplot, annotations by cluster. (B) Mesoderm WT atlas marker gene dotplot, annotations by cluster. Dot size represents percent of cells expressing gene, color represents average expression level. Relative cluster number used when annotation not possible.

Development • Supplementary information

Fig. S3

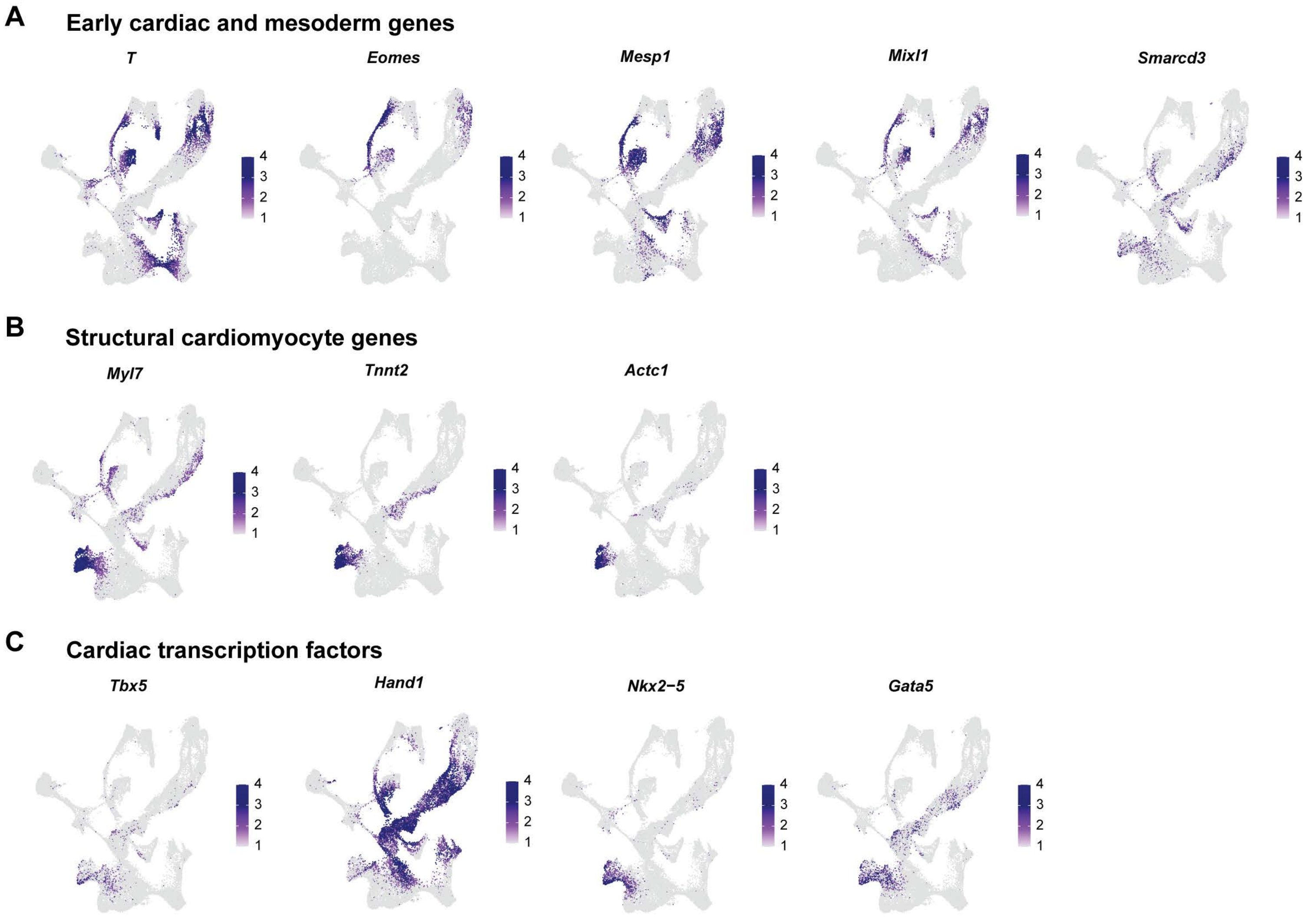

Fig. S3. Co-expression of cardiac mesoderm genes in *Smarcd3*-F6+ cell types. WT mesoderm atlas UMAP feature plots for expression of (A) early cardiac and mesoderm genes *T, Eomes, Mesp1, Mixl1, Smarcd3*, (B) structural cardiomyocyte genes *Myl7, Tnnt2, Actc1*, and (C) cardiac transcription factors *Tbx5, Hand1, Nkx2-5, Gata5*.

Development • Supplementary information

Fig. S4

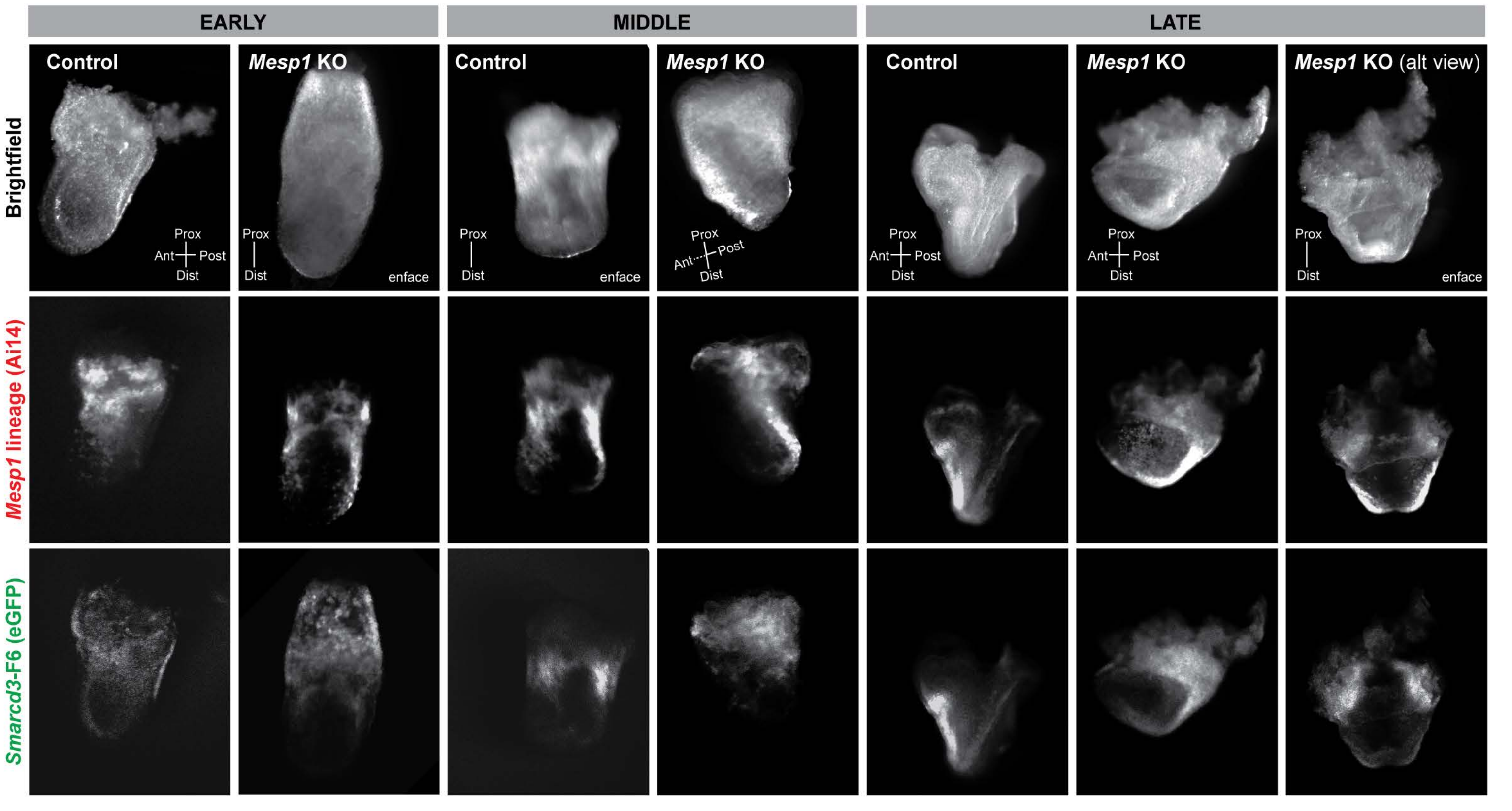

Fig. S4. Posterior *Smarcd3*-F6+ cells in *Mesp1* KO embryos. Representative images of fluorescent transgene reporters in *Mesp1* KO embryos at Early, Middle, and Late relative developmental stages.

Development • Supplementary information

Fig. S5

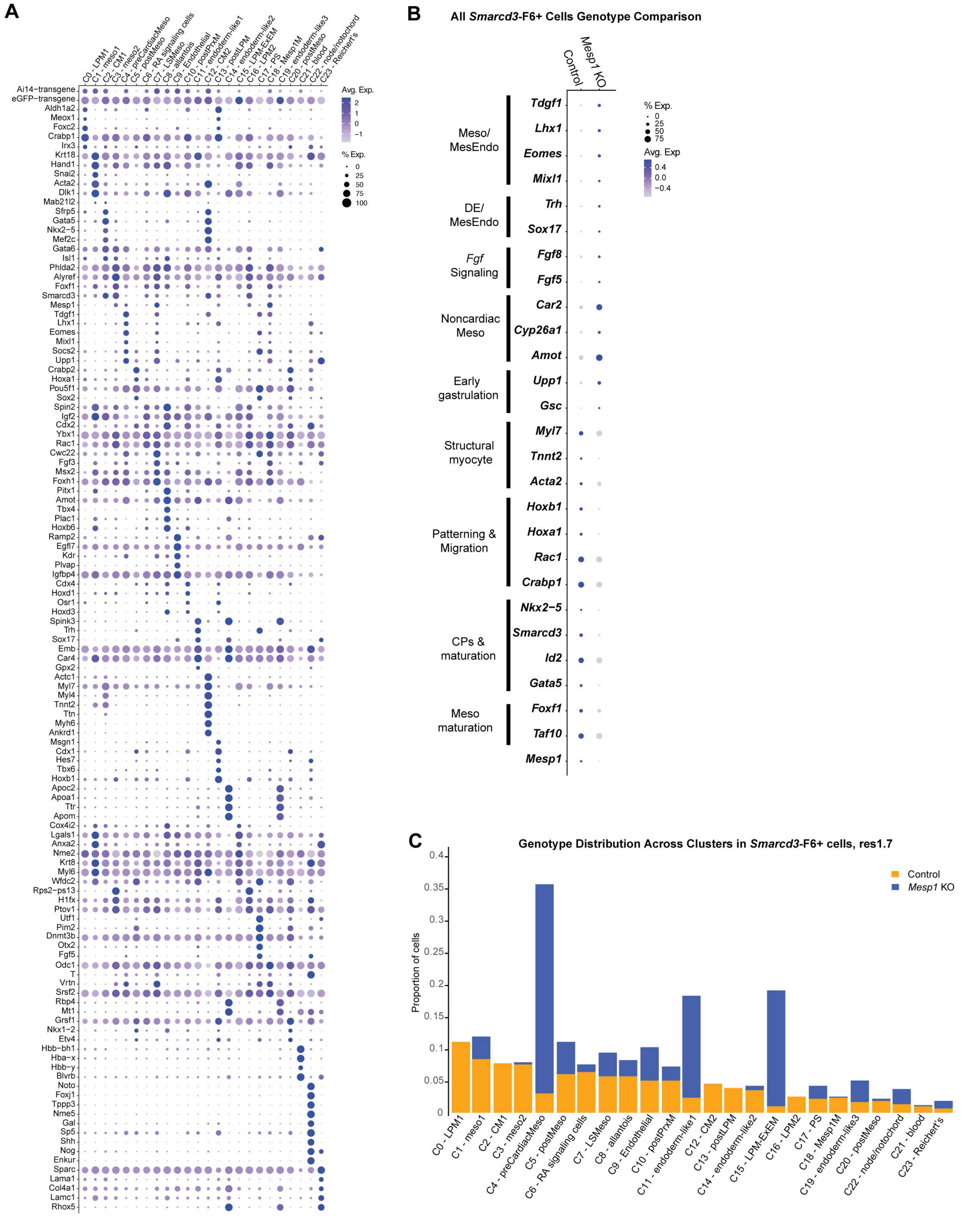

Fig. S5. *Smarcd3*-F6+ cells transcriptional profiles. (A) *Smarcd3*-F6+ cell WTvsMut atlas marker gene dotplot, annotations by cluster. (B) Dotplot of DGE between genotypes across all cells. Dot size represents percent of cells expressing gene, color represents average expression level. (C) Barplot of genotype proportions across clusters.

Fig. S6

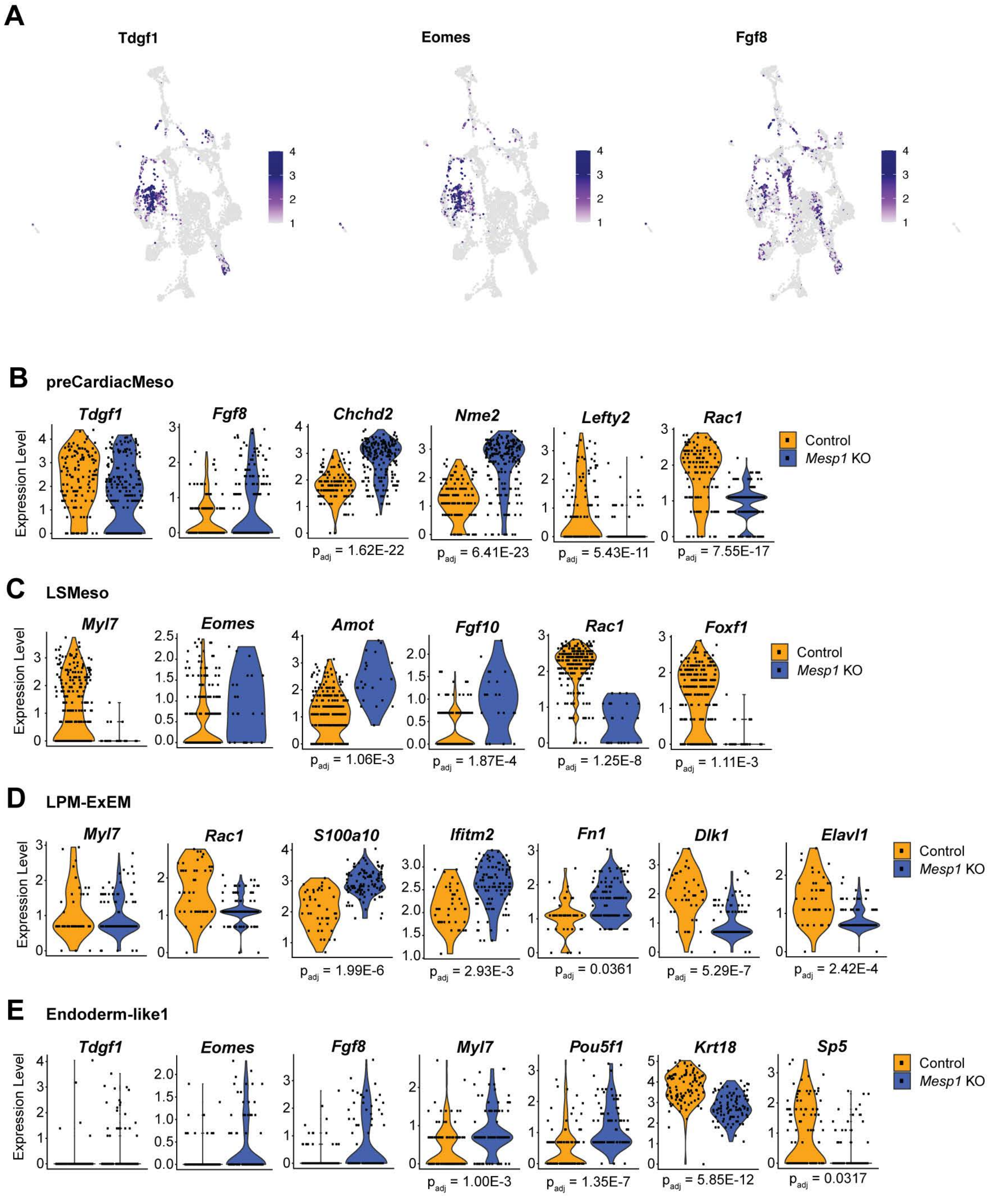

Fig. S6. DGE in *Smarcd3*-F6+ cells from *Mesp1* KO embryos. (A) UMAP feature plots of early cardiac marker gene expression; *Tdgf1*, *Eomes*, *Fgf8*, *Myl7*. (B-E) DGE profiling highlights similar cardiac marker gene expression between genotypes in (B) preCardiacMeso, (C) LSMeso, (D)LPM-ExEM, and (E) endoderm-like1 cells. (B-E) Significant adj p values < 0.05.

Development • Supplementary information

Fig. S7

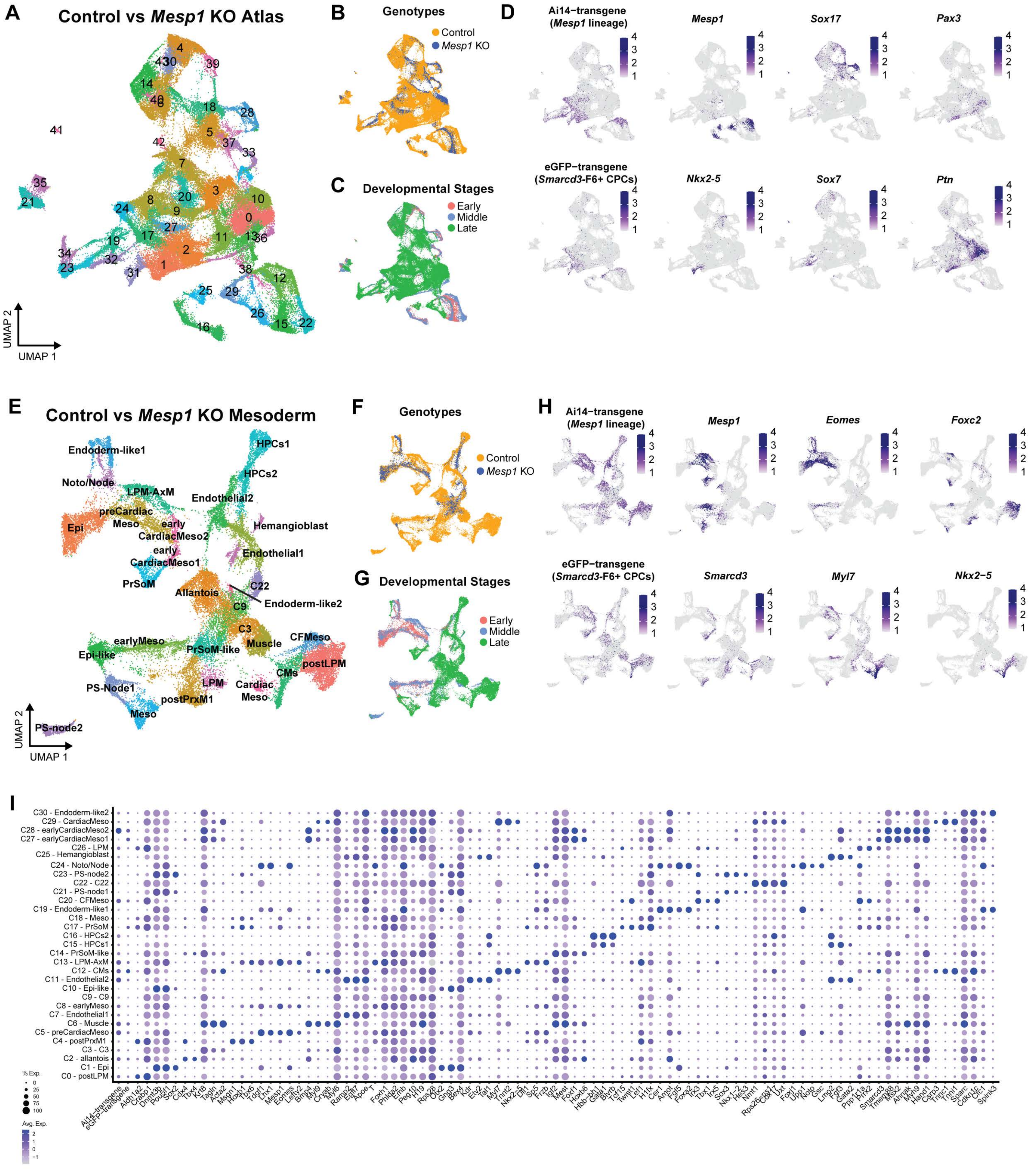

Fig. S7. Identification of emerging cardiac mesoderm in control and *Mesp1* KO embryo scRNA-seq data. (A) Whole embryo atlas UMAP of 96,027 cells with overlay of (B) genotypes and (C) relative developmental stages. (D) UMAPs for expression of *Mesp1* lineage transgene Ai14, CPC-specific *Smarcd3*-F6 transgene eGFP, cardiac mesoderm markers *Mesp1*, *Nkx2-5,* endoderm markers *Sox17*, *Sox7*, neural markers *Pax3*, *Ptn*. (E) Mesoderm atlas UMAP of 35,792 cells with overlay of (F) genotypes and (E) relative developmental stages. (H) UMAPs for cardiac and mesoderm marker gene expression. (I) Doplot of marker genes and annotations by cluster in mesoderm atlas. Dot size represents percent of cells expressing gene, color represents average expression level. Relative cluster number used when annotation not possible.

Fig. S8

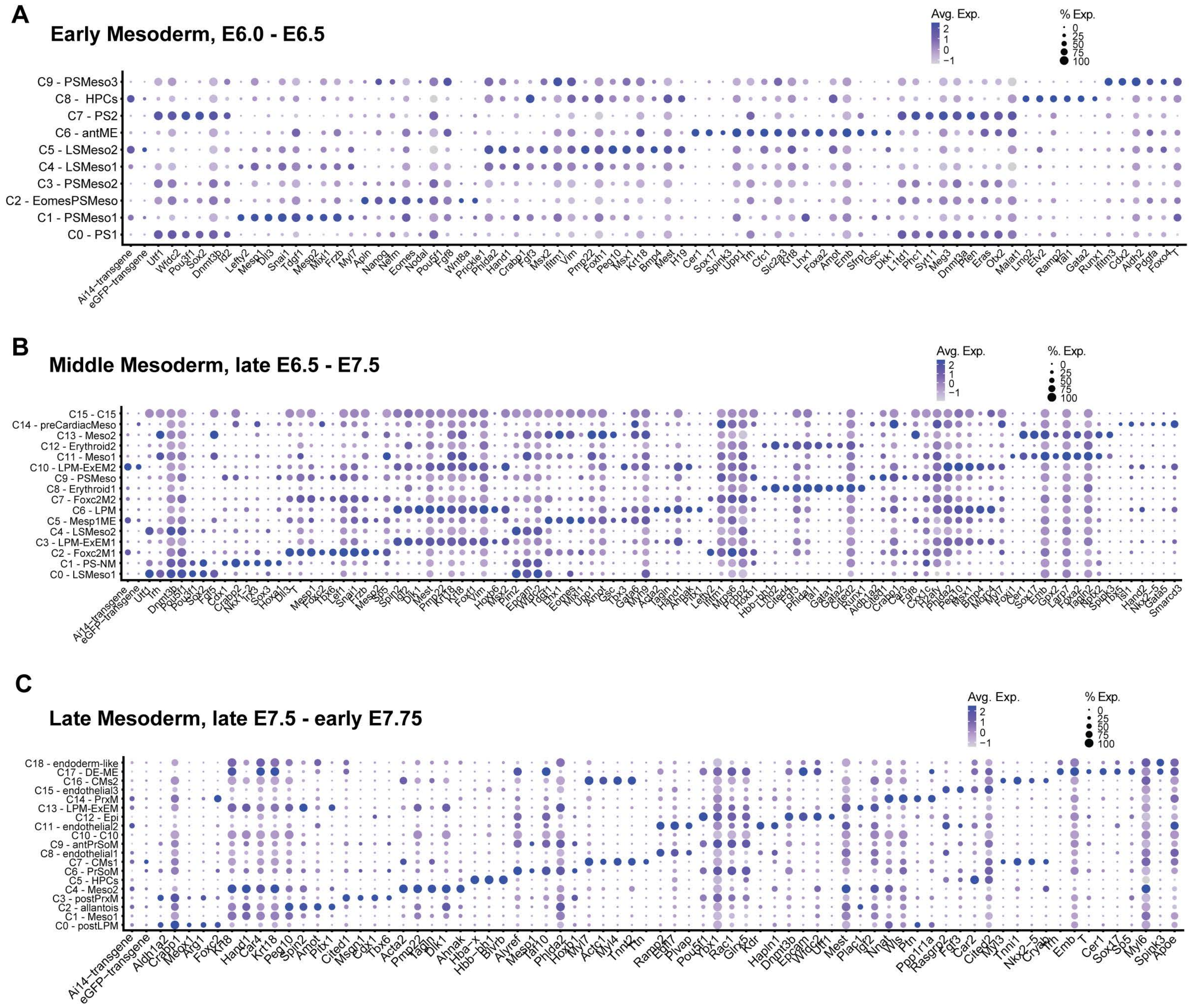

Fig. S8. Cell type labels in mesoderm developmental stages atlases. Dotplot of marker genes and annotations by cluster in atlases for (A) Early mesoderm, (B) Middle mesoderm, (C) Late mesoderm. Dot size represents percent of cells expressing gene, color represents average expression level. Relative cluster number used when annotation not possible.

Development • Supplementary information

Fig. S9

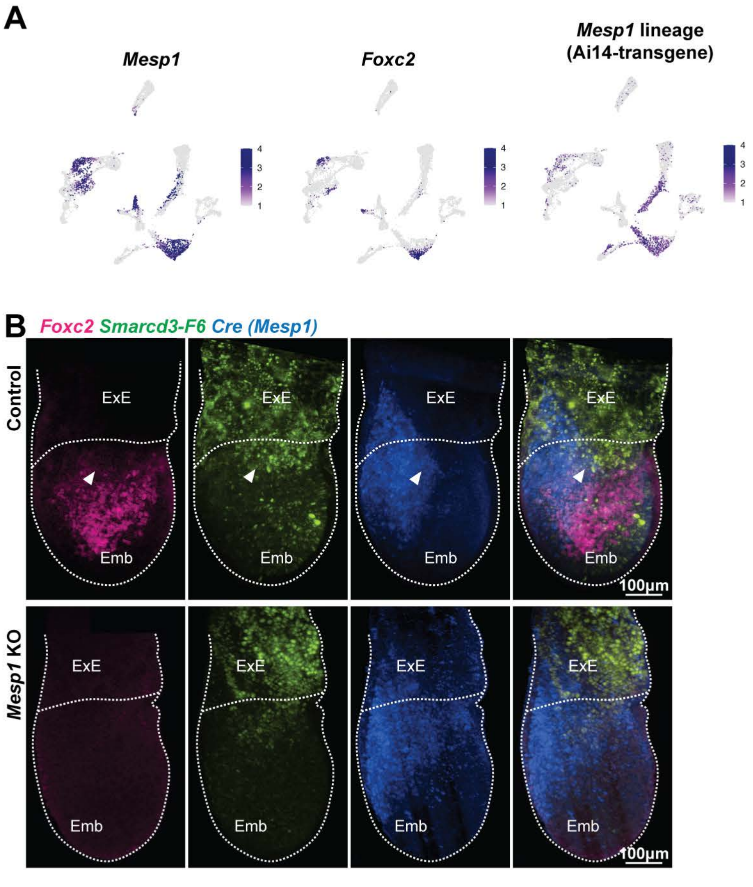

Fig. S9. Disrupted organization of mesoderm in *Mesp1* KO embryos. (A) Middle mesoderm atlas UMAP feature plots for *Mesp1*, *Foxc2*, and Ai14 *Mesp1*-lineage gene expression. (B) Immunostaining and Light Sheet Confocal microscopy for *Foxc2* (magenta), *Smarcd3*-F6 (green) and *Mesp1* via Cre detection (blue) in Middle stage embryos (~E6.75). Arrowheads denote domain boundaries in control and disruption in *Mesp1* KO embryo. Scale bars are 100 µm.

Development • Supplementary information

Fig. S10

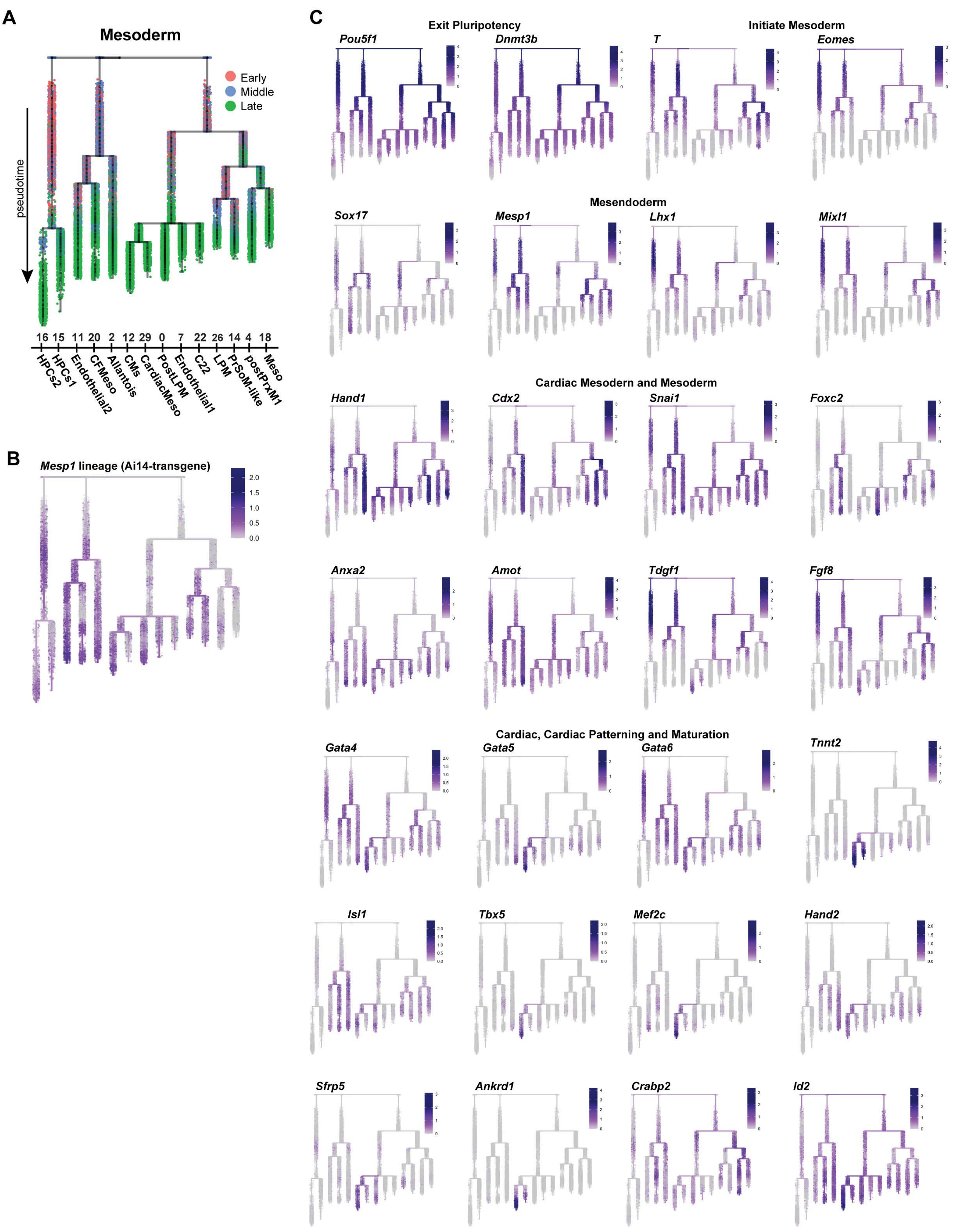

Fig. S10. URD trajectory for pseudotime ordering of control and *Mesp1* KO mesoderm. (A) URD tree labeled with embryo relative developmental stages. (B) URD tree of Ai14 (*Mesp1* lineage) transgene expression. (C) URD trees for expression of various mesodermal genes and TFs involved in cardiogenesis.

Fig. S11

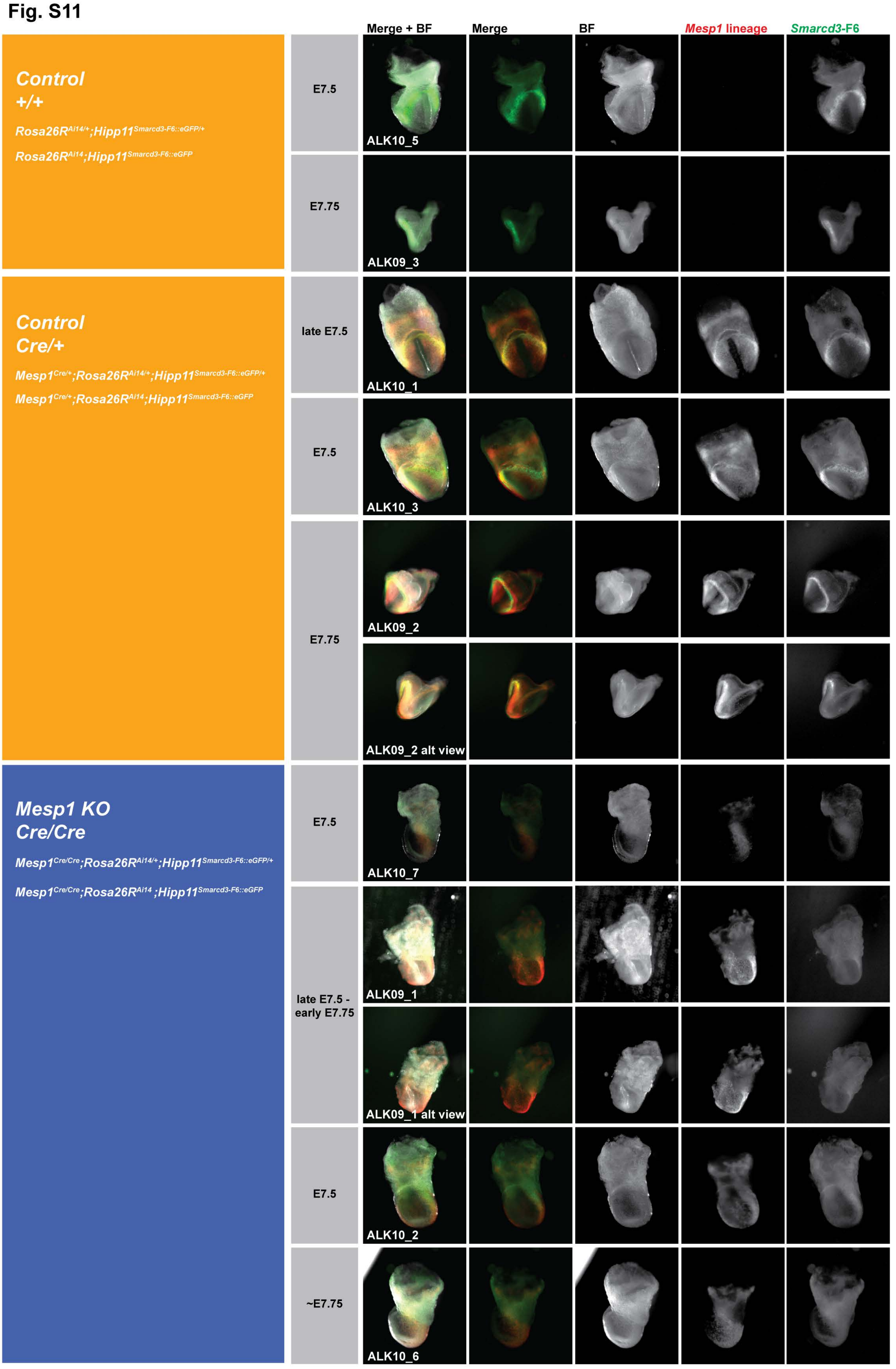

Fig. S11. Middle- and Late-stage scATAC-seq embryos. Embryos utilized in generation of control and *Mesp1* KO scATAC-seq dataset. Endogenous fluorescence for transgenic reporters Ai14 (*Mesp1* lineage) and eGFP (*Smarcd3*-F6+ CPCs) Images not acquired nor processed identically.

Development • Supplementary information

Fig. S12

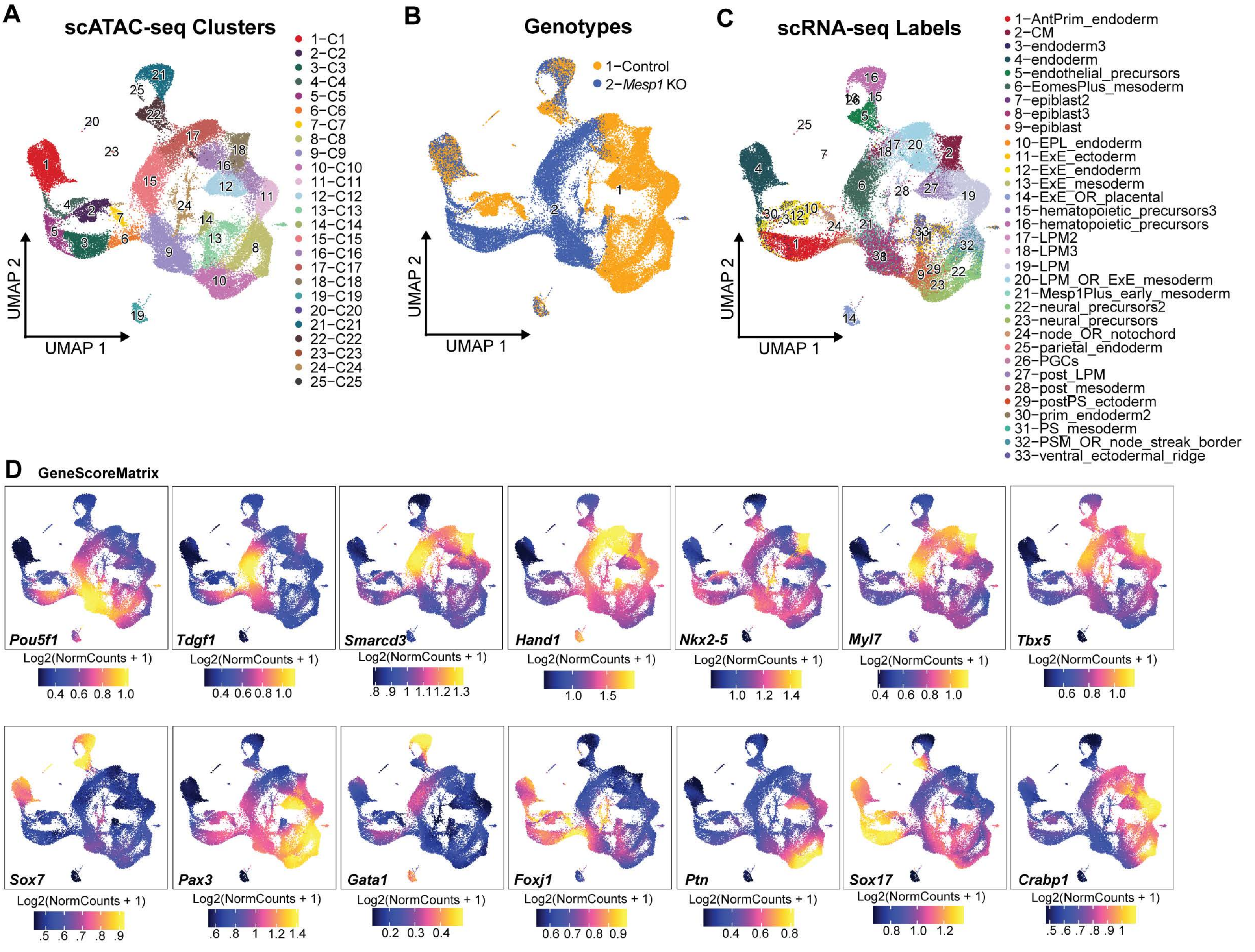

Fig. S12. Identification of mesoderm in scATAC-seq data from whole embryos. (A) Whole embryo scATAC-seq atlas with overlays for (B) genotype and (C) relative cell annotations from integration with scRNA-seq. (D) GeneScoreMatrix plots for chromatin accessibility loci of various mesoderm, cardiac, endoderm, ectoderm, neuronal marker genes.

Fig. S13

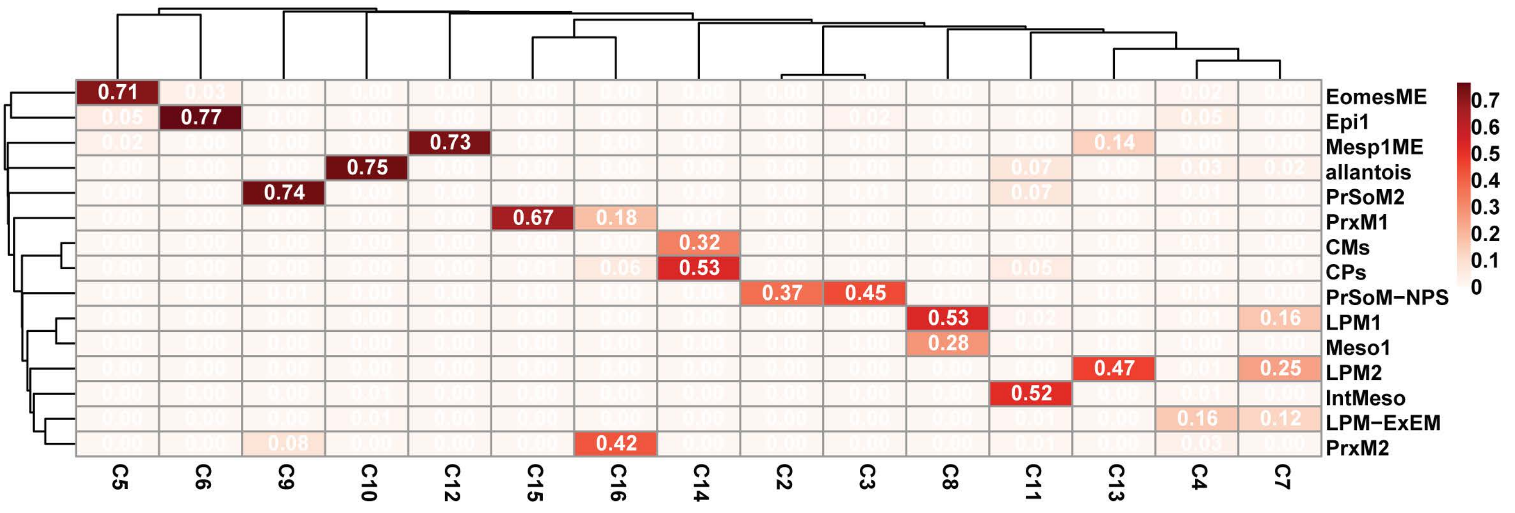

Fig. S13. Jaccard Similarity Index for scATAC-seq cluster annotation. Scaled strength of similarity (Values 0-1) match for scRNA-seq label transfer (rows) onto scATAC-seq clusters (columns) for relative cell type annotations. Greater values indicate stronger label matching.

Development • Supplementary information

Fig. S14

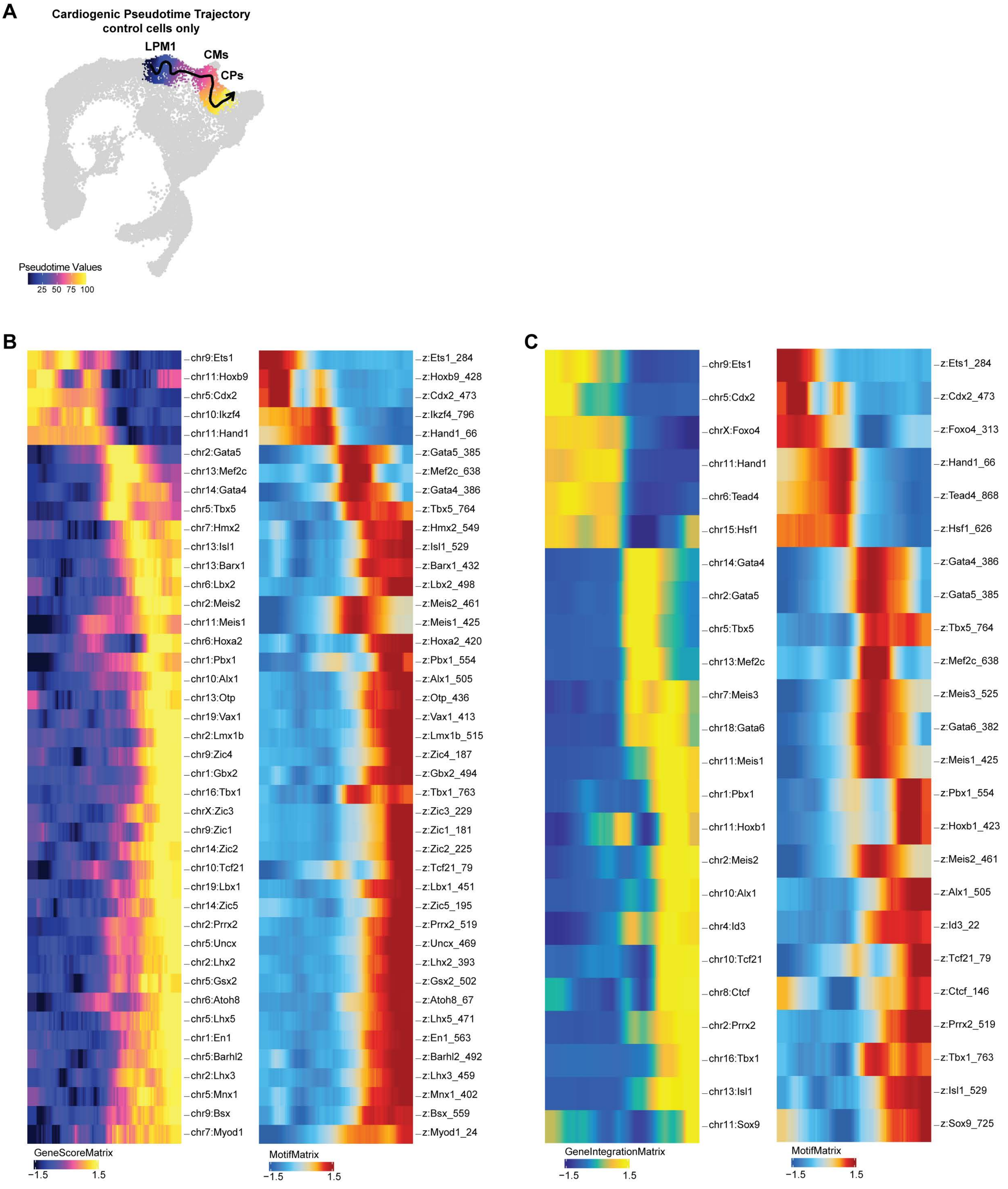

Fig. S14. Control cell scATAC-seq pseudotime trajectory. (A) Control cell cardiac-fate trajectory path colored by pseudotime values. (B-C) Heatmap visualizations of dynamic shifts in pseudotime for correlation matrices between (B) accessibility near TF loci, GeneScoreMatrix, with associated TF motifs, MotifMatrix and (C) TF gene expression, GeneIntegrationMatrix, with associated TF motifs, MotifMatrix.

Development • Supplementary information

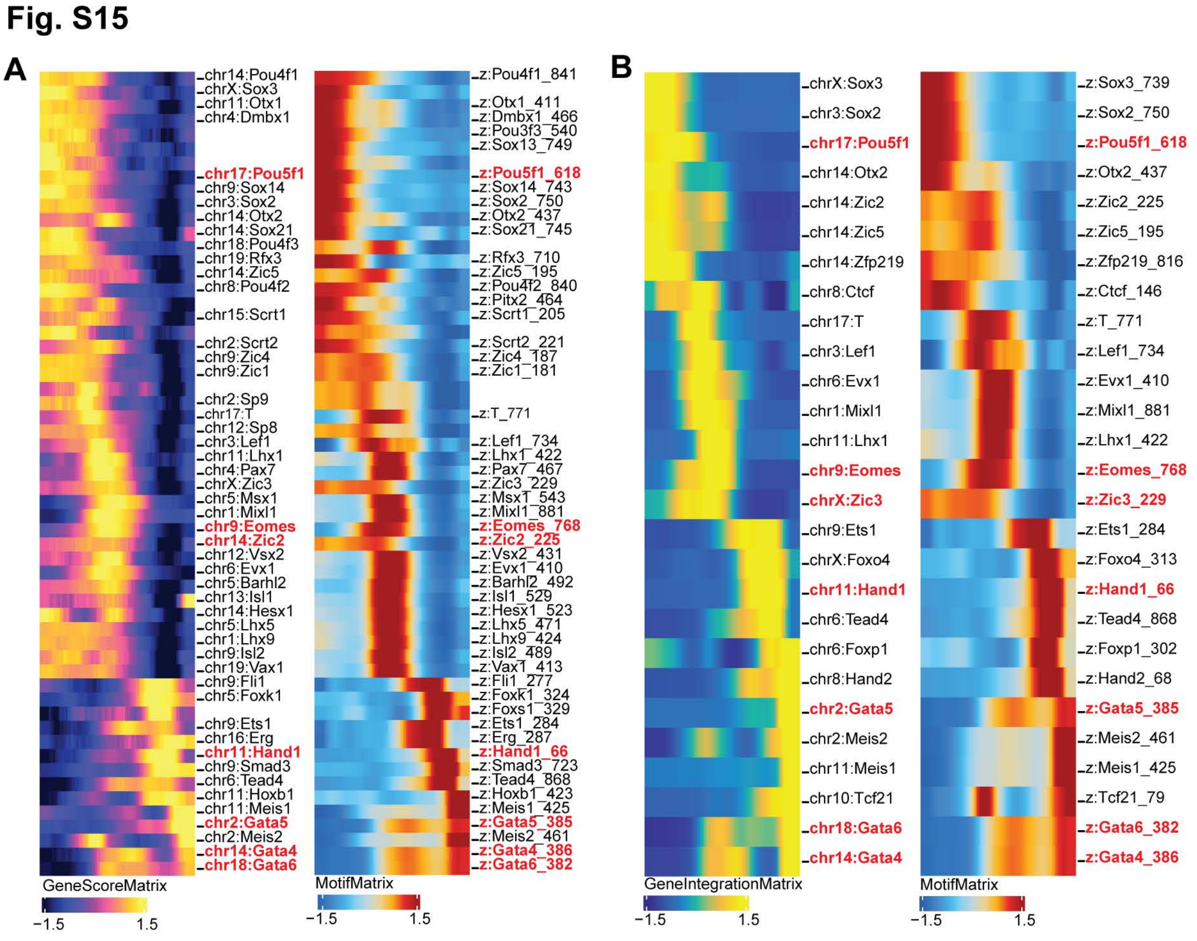

Fig. S15. Mutant integrative scATAC-seq trajectory pseudotime correlation analysis. Heatmap visualizations of dynamic shifts in pseudotime for correlation matrices between (A) accessibility near TF loci, GeneScoreMatrix, with associated TF motifs, MotifMatrix and (B) TF gene expression, GeneIntegrationMatrix, with associated TF motifs, MotifMatrix. Putative positive regulators in red.

Fig. S16

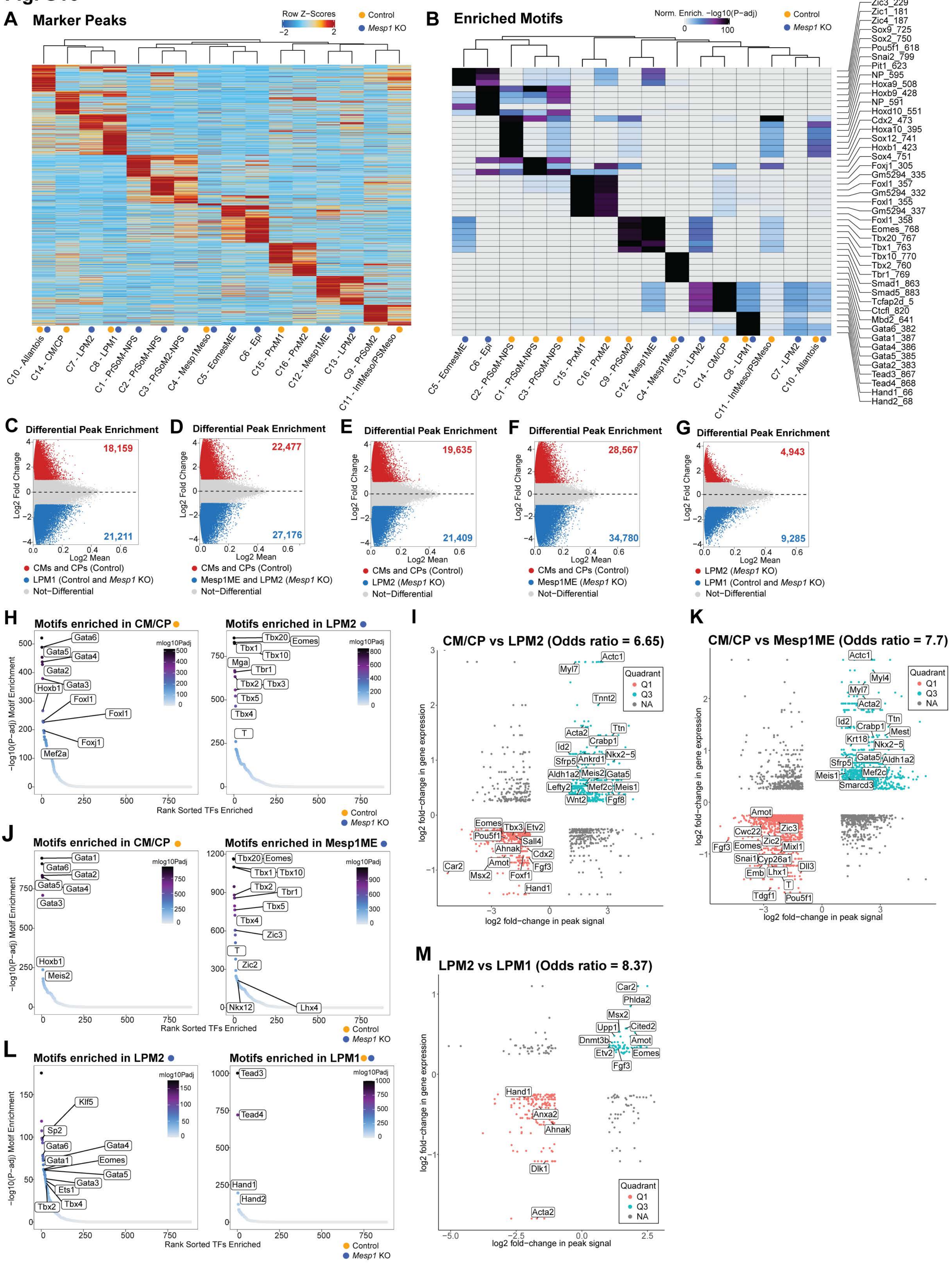
A Marker Peaks
Row Z-Scores
Control
Mesp1 KO
B Enriched Motifs
Norm. Enrich. −log10(P-adj)
C Differential Peak Enrichment
18,159
21,211
CMs and CPs (Control)
LPM1 (Control and Mesp1 KO)
Not−Differential
D Differential Peak Enrichment
22,477
27,176
Mesp1ME and LPM2 (Mesp1 KO)
E Differential Peak Enrichment
19,635
21,409
LPM2 (Mesp1 KO)
F Differential Peak Enrichment
28,567
34,780
Mesp1ME (Mesp1 KO)
G Differential Peak Enrichment
4,943
9,285
Log2 Mean
Log2 Fold Change
H Motifs enriched in CM/CP
Motifs enriched in LPM2
I CM/CP vs LPM2 (Odds ratio = 6.65)
K CM/CP vs Mesp1ME (Odds ratio = 7.7)
J Motifs enriched in CM/CP
Motifs enriched in Mesp1ME
L Motifs enriched in LPM2
Motifs enriched in LPM1
M LPM2 vs LPM1 (Odds ratio = 8.37)
Rank Sorted TFs Enriched
−log10(P−adj) Motif Enrichment
log2 fold−change in gene expression
log2 fold−change in peak signal
Quadrant
Q1
Q3
NA

Fig. S16. Differential peak and motif enrichment in cardiogenic cell types of control and *Mesp1* KO mesoderm cells. (A) Marker Peaks heatmap (FDR <= 0.05, Log2FC >=1) accessibility profiles of mesoderm cell types comprised of control, *Mesp1* KO, or both genotypes. (B) Motif enrichment heatmap (FDR <=0.05, Log2FC >=1) in cluster Marker Peaks. (C-G) MA plots of pairwise differential peak comparisons between cardiogenic cell types with genotypes noted. (H, J, L) Motifs enriched in differentially accessible peaks. (I, K, M) Plots for peak,gene associations; correlations between differential peak accessibility and gene expression in cell type1 vs type2. Q3 peak,gene pairs are significantly more accessible peaks paired with upregulated gene expression in type1 cells. Q1 peak,gene pairs are significantly more accessible peaks paired with upregulated gene expression in type2 cells. Odds ratio denotes observed peak,gene relationship probability. Peak,gene association plots for (I) CMCP vs LPM2, (K) CMCP vs Mesp1ME, (M) LPM2 vs LPM1.

Development • Supplementary information

Fig. S17

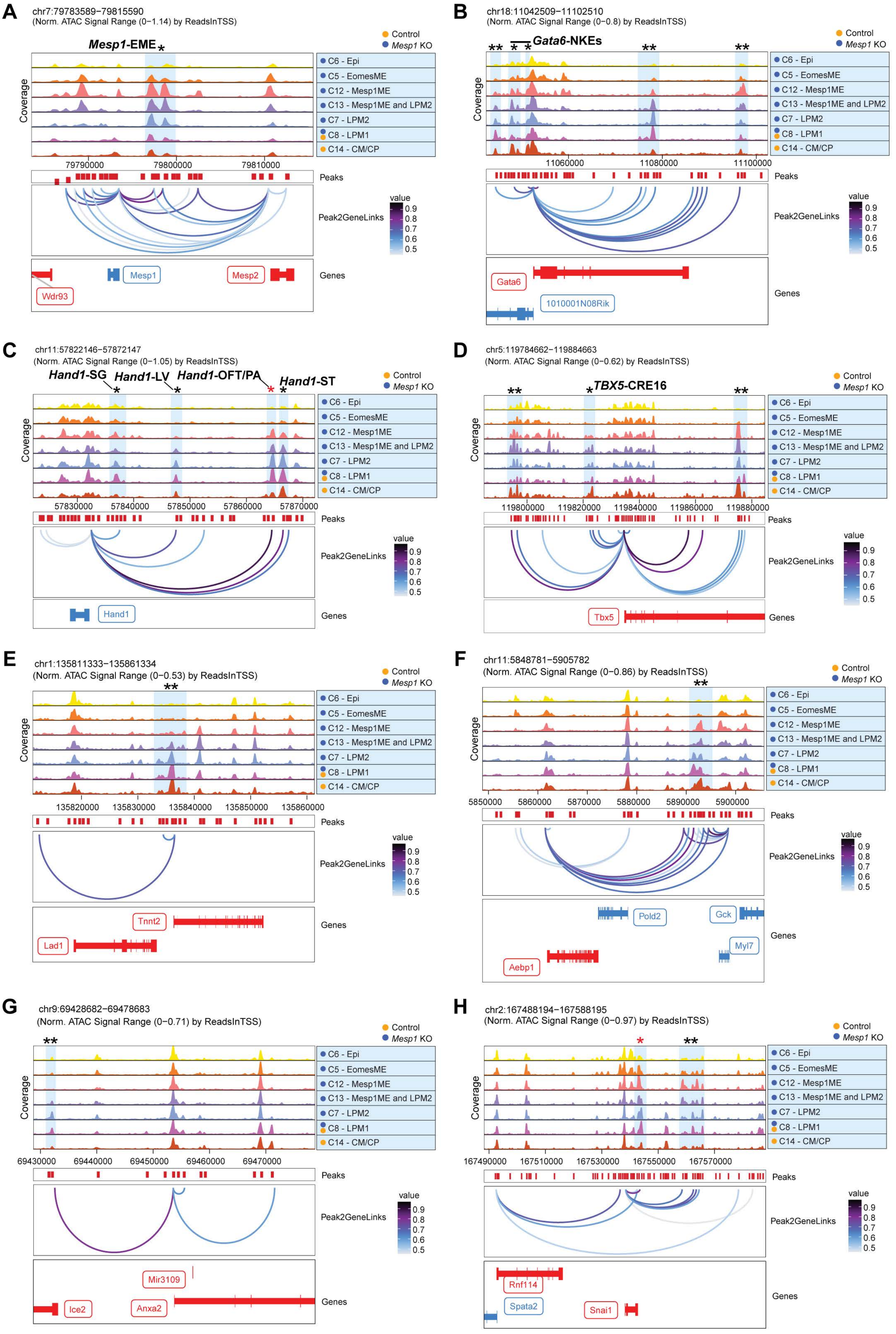

Development • Supplementary information

Fig. S17. Peak2Gene linkage plots for dysregulated genes in *Mesp1* KO embryos. Cell type genome tracks for Peak2Gene linkage predictions of regulatory connections between distal accessible regions (Peaks) and nearby genes. Blue vertical bars denote predicted distal regulatory regions; *denotes characterized elements, named when available; red* denotes regions with Mesp1-binding; **denotes uncharacterized elements. Peak2Gene lineages for (A) *Mesp1* (B) *Gata6,* (C) *Hand1,* (D) *Tbx5,* (E) *Tnnt2*, (F) *Myl7*, (G) *Anxa2*, (H) *Snai1*.

Development • Supplementary information

Fig. S18

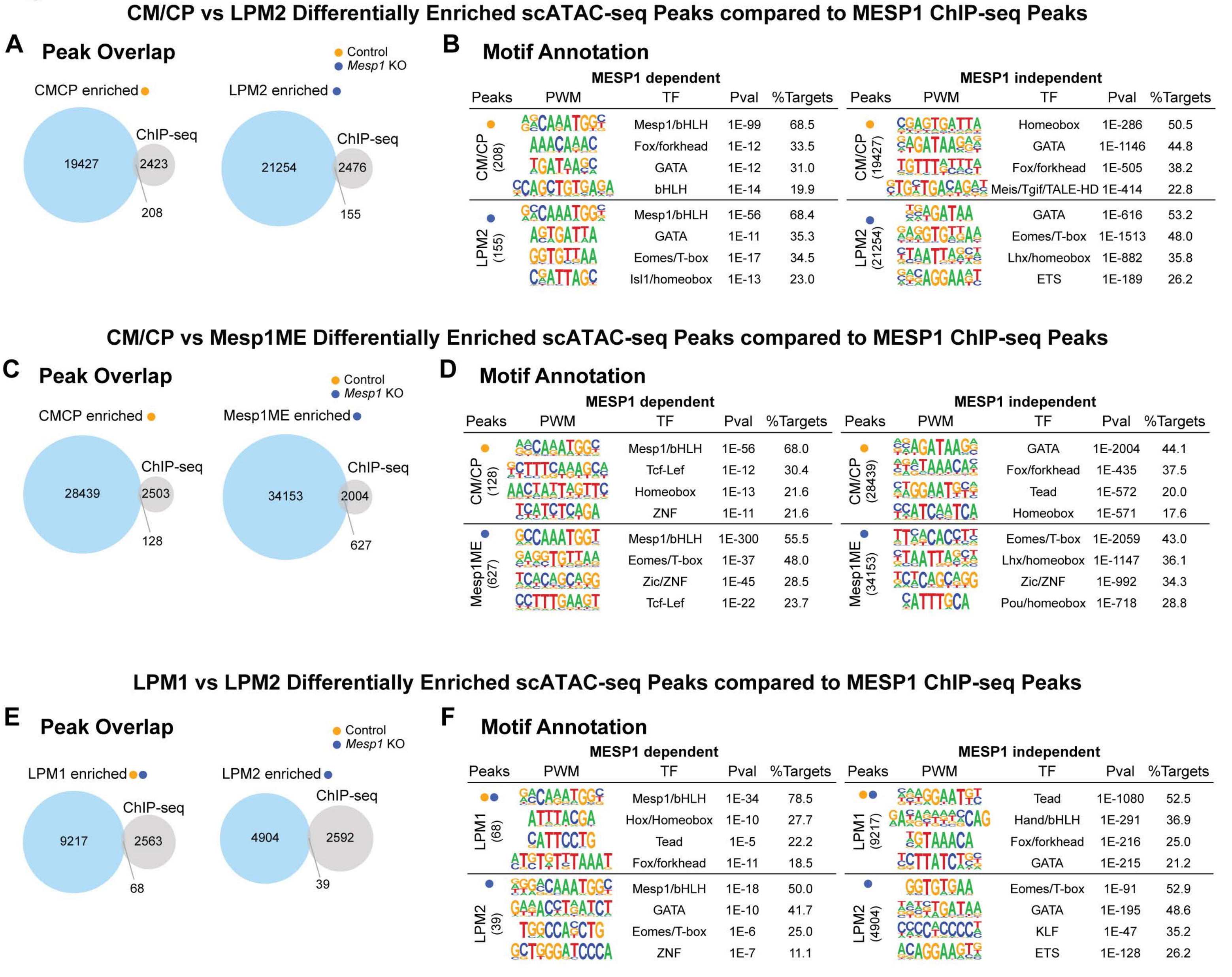

Fig. S18. MESP1 dependence and dysregulated regulatory networks in *Mesp1* KO embryos. (A,C,E) MESP1 ChIP-seq compared to enriched peaks in differentially accessible regions between control and mutant cell types in (A) CM/CP vs. LPM2, (C) CM/CP vs. Mesp1ME, (E) LPM1 vs. LPM2. (B,D,F) TF binding motif annotations for MESP1-dependent and -independent differentially accessible regions.

Development • Supplementary information

Table S1. Wildtype gastrulation single cell atlas gene expression. Corresponds to Fig. 1.

Click here to download Table S1

Table S2. *Mesp1 KO* Smarcd3-F6^{+} single cell atlas and differential gene expression. Corresponds to Fig. 2.

Click here to download Table S2

Table S3. *Mesp1* KO gastrulation and mesoderm single cell atlas and differential gene expression. Corresponds to Fig. 3.

Click here to download Table S3

Table S4. *Mesp1* KO mesoderm pseudotime differential gene expression. Corresponds to Fig. 4.

Click here to download Table S4

Table S5. *Mesp1* KO mesoderm single cell chromatin accessibility atlas and transcriptional regulators. Corresponds to Fig. 5.

Click here to download Table S5

Table S6. Differential expression and accessibility of key genomic regulators in *Mesp1* KO cardiogenic cell types. Corresponds to Fig. 6.

Click here to download Table S6
