# Supplementary material for: A Mesp1-dependent developmental breakpoint in transcriptional and epigenomic specification of early cardiac precursors
Source: Development. 2023 May 2;150(9):dev201229. doi: 10.1242/dev.201229 (PMC10259516; doi:10.1242/dev.201229)
Supplement: Supplementary file 1 [file develop-150-201229_review_history.pdf]

A *Mesp1*-dependent developmental breakpoint in transcriptional and epigenomic specification of early cardiac precursors

Alexis Leigh Krup, Sarah A. B. Winchester, Sanjeev S. Ranade, Ayushi Agrawal, W. Patrick Devine, Tanvi Sinha, Krishna Choudhary, Martin H. Dominguez, Reuben Thomas, Brian L. Black, Deepak Srivastava and Benoit G. Bruneau
DOI: 10.1242/dev.201229

Editor: Liz Robertson

Review timeline

Original submission:	23 August 2022
Editorial decision:	20 September 2022
First revision received:	31 January 2023
Editorial decision:	21 February 2023
Second revision received:	6 March 2023
Accepted:	21 March 2023

Original submission

First decision letter

MS ID#: DEVELOP/2022/201229

MS TITLE: A Mesp1-dependent developmental breakpoint in transcriptional and epigenomic specification of early cardiac precursors

AUTHORS: Alexis Leigh Krup, Sarah Winchester, Sanjeev Ranade, Ayushi Agrawal, W. Patrick Devine, Tanvi Sinha, Krishna Choudhary, Martin Dominguez, Reuben Thomas, Brian L Black, Deepak Srivastava, and Benoit Bruneau

I have now received all the referees' reports on the above manuscript, and have reached a decision. The referees' comments are appended below, or you can access them online: please go to BenchPressand click on the 'Manuscripts with Decisions' queue in the Author Area.

As you will see, the referees express considerable interest in your work, but have some significant criticisms and recommend a substantial revision of your manuscript before we can consider publication. If you are able to revise the manuscript along the lines suggested, which may involve further experiments, I will be happy receive a revised version of the manuscript. Your revised paper will be re-reviewed by one or more of the original referees, and acceptance of your manuscript will depend on your addressing satisfactorily the reviewers' major concerns. Please also note that Development will normally permit only one round of major revision. If it would be helpful, you are welcome to contact us to discuss your revision in greater detail. Please send us a point-by-point response indicating your plans for addressing the referee's comments, and we will look over this and provide further guidance.
Please attend to all of the reviewers' comments and ensure that you clearly highlight all changes made in the revised manuscript. Please avoid using 'Tracked changes' in Word files as these are lost in PDF conversion. I should be grateful if you would also provide a point-by-point response detailing how you have dealt with the points raised by the reviewers in the 'Response to Reviewers' box. If you do not agree with any of their criticisms or suggestions please explain clearly why this is so.

© 2023. Published by The Company of Biologists under the terms of the Creative Commons Attribution License (https://creativecommons.org/licenses/by/4.0/).

Reviewer 1

Advance summary and potential significance to field

Important new insight into trancriptional control of heart development
Important datasets that are very valuable to the field

Comments for the author

In the manuscript by Krup et al, the authors explore Mesp1's role in early cardiogenesis at single cell resolution, specifically to address questions surrounding cardiogenic fate decisions in its absence. The authors results suggest that Mesp1 is required for robust activation of early cardiogenic TF's, such as Gata4 and Hoxb1, and allows for progression through cardiogenesis i.e. activation of Nkx2-5. The authors also identified that some mid-to-late cardiogenic programs dictated by Eomes are Mesp1-independent and still progress. The overall conclusion of the work is that transient Mesp1 expression during development is required to push early cardiogenic progenitors through to late-stage progenitors. This manuscript is of high interest for the cardiac development field and addresses the role of Mesp1 in the process. This manuscript also provides novel datasets that can be used widely.

Comments:
1. It's remarkable how distinct the chromatin accessibility landscape is between control and Mesp1 KO cells (Fig. 5B & S11B). Notably absent from the motif analyses is Mesp1 itself. Was the Mesp1 motif enriched among sites that change between control and mutant?
2. Related to question 1, the authors cite a recent paper (Lin et al. 2022) that describes in vitro induction of Mesp1 generates GATA4 accessible sites. Do the authors believe MESP1 acts as a pioneer-like factor to make GATA4 target sites accessible or is GATA4 providing that role? For example, in the list of sites that are not accessible in the mutant compared to the control with GATA4 motifs, do these sites also contain MESP1 motifs? Using the pseudotime of accessibility, are there any clues about order of TF binding/accessibility gains with respect to these sites perhaps using footprinting analysis?
3. In the model described, there exists a breakpoint in an early cardiac progenitor. Weak expression of GATA4 and absence of NKX2-5 prevents these cells from progressing to mature cardiomyocytes in Mesp1 mutants. Based on the results described, such as ectopic localization of transgene expressing cells and gain of cardiac markers in other cell types in the mutants, these cells appear to be determined in their cell fate. Using the scATAC-seq, e.g., the sites that are still accessible in the mutant, and the earlier stage scRNA-seq, are there any clues that may suggest what may be acting upstream or in parallel of Mesp1 to determine cardiac fate? Given the continued expression of the Mesp1-indenepdent Eomes mechanism described, could Eomes be part of the cardiac cell fate determination process?

Reviewer 2

Advance summary and potential significance to field

In their manuscript, Leigh Krup et al. seek to understand cardiac precursor cell (CPC) specification during gastrulation and the role of the transcription factor Mesp1 in this process. They have used single-cell transcriptomics and single-cell chromatin accessibility in control and Mesp1KO mouse embryos. Using a transgene previously characterized as cardiac specific, they show perdurance of expression of the transgene in Mesp1KO embryos, which is however mislocated. They used a double transgenic approach to curate scRNA-seq data for cardiac derivatives. With this approach they show that Smarcd3-F6 is finally expressed (even if weak) more broadly also in non-cardiac derivative (including extraembryonic and presomitic mesoderm). They show that Mesp1KO cells initiate the early steps of cardiac specification but are then blocked in a state where Hand1 and Eomes are highly expressed and cannot differentiate into the cardiomyocyte lineages. These results are complementary to previous analysis of Mesp1KO cells in vivo but the previous analysis was performed at early stages of development. Finally, the main novelty of the paper is the single cell chromatin accessibility analysis in both control and KO cells performed in vivo for the first time at these early stages. It has revealed some Mesp1-dependent and Mesp1-independent regulation of key

© 2023. Published by The Company of Biologists under the terms of the Creative Commons Attribution License (https://creativecommons.org/licenses/by/4.0/).

cardiac genes in vivo and describe very interesting regulatory regions that are progressively activated along cardiac specification.

Comments for the author

Overall, this manuscript is important for the field of cardiac development as there has recently been growing interest in the early steps of cardiac development during gastrulation. It is likely that understanding gastrulation and cardiac specification will be important to better understand congenital heart diseases. We encourage publication with some revisions to improve the manuscript and facilitate its reading for non-specialists.

Generally, the study is very interesting but complicated with the description of a large amount of bioinformatical analyses. This could be easily improved with few text edits or clarifications. It is sometimes unclear in the manuscript (and figures) which dataset is analyzed (all cells, curated for dual transgene expression or Smarcd3-F6+ only) and what are the differences obtained from analyzing Smarcd3-F6+ and dual transgene expression. Similarly, adding stages axes and legends in pictures of embryos would help the reader to better appreciate the results.
The scATAC-seq in vivo is a real asset for this study, specifically to show chromatin regulation of early mesoderm, cardiac and non-cardiac genes in Mesp1KO or control cells. Since Mesp1-Chip seq data in vitro is now available (Lin et al. 2022) crossing the ATAC-seq data with Mesp1 peaks could be important to differentiate Mesp1-direct and Mesp1-indirect effects.

This study has focused on trajectories from epiblast cells toward the cardiomyocyte lineages but could extend, without further experiments, on other cardiac lineages (endothelial) as well as other lineages including the extraembryonic lineages which has been recently linked to cardiac development (Tyser et al. 2021, Wang et al. 2022).

Please find below more specific suggestions for improvement of the manuscript:

Specific comments:
1. For clarification and easy reading of the manuscript, I would suggest to clarify the label of the UMAP representations in the different figures. For example, it is not clear whether the subsetted cell clusters in Fig1D corresponds to all mesoderm clusters or specifically to Ai14 and eGFP + clusters. Adding the label (Smarcd3-F6+) as in Fig 2B will help the reader to understand that the data have been subsetted based on eGFP expression.

2. The first part of the results describes the scRNAseq data. The authors nicely describe in subsetted clusters (Fig 1D) that the Smarcd3-F6+ transgene is expressed in clusters related to early cardiac development (mesoderm exiting the PS, anterior mesoderm, lateral plate mesoderm and cardiomyocytes)
a. The authors do not comment on the expression of Smarcd3-F6 in non-cardiac clusters annotated as the allantois, extraembryonic mesoderm, posterior lateral plate mesoderm and paraxial mesoderm. This is only described later when analyzing Mesp1KO embryos (see lines 135-146), which could be a bit confusing. It would probably be easier for the reader to describe all Smarcd3-F6+ clusters in the first paragraph along with the description of cardiac related clusters.
b. In addition, it is not clear whether the 4,868 Smarcd3-F6+ cells (line 135) refer to control or Mesp1KO cells.
c. A WT vs. KO Mesp1/Smarcd3-F6 lineage time course (from E6-E7.5) would be really interesting so we could visualize exactly what populations are being subsetted for all the bioinfo analyses? Maybe an addition to Fig. S1 or another panel with KO embryos at similar stages?

3. When comparing scRNAseq data from Mesp1KO and control cells, the authors nicely show that Mesp1KO cells are not represented in every cell type.
a. In FigS4D, it might be better representing the data in term of relative proportion (to the total number of cells per genotype) rather than absolute number of cells. From the figure it seems that there are less Mesp1KO than control cells.
b. It seems that while some clusters are not completely lacking Mesp1KO cells (allantois, posterior LPM, endothelium), the proportion is decreased. On the other side some clusters showed a higher proportion of Mesp1KO cells (pre-cardiac mesoderm, LPM, extraembryonic mesoderm). Can the authors comment and extend further their analysis on this point?

© 2023. Published by The Company of Biologists under the terms of the Creative Commons Attribution License (https://creativecommons.org/licenses/by/4.0/).

4. Can Mesp2 compensate for the loss of Mesp1 and explain the transcriptional resilience and vulnerability seen in Mesp1KO cells? Knowing the redundance between Mesp1 and Mesp2, it would be important to show its expression. For the reader, it seems important to specify whether Mesp1Cre/Cre embryos show a decreased expression of Mesp2 and to which level. Is there remaining Mesp2 expression in some clusters?

5. The authors have used URD for pseudotemporal trajectory ordering. The analysis is very interesting and relevant. It nicely shows the branches that lacks Mesp1KO cells.
a. On the other hand, the authors underline that Mesp1KO cells are also found in its own pseudotime branch. This is really relevant to the study and could be further investigated. What is the fate of these cells? Is it a transition state?
Could it correspond to cells found in the anterior part of the embryo in Mesp1KO embryos?
b. Additionally, it is worth noting that the endothelial, allantois and CFmeso branching lack Mesp1KO cells. This could also be further discussed. Since these branches express Smarcd3-F6-eGFP and somehow Myl7 and Nkx2-5, do they correspond to the endothelium/endocardial lineages and part of the juxtacardiac field)?
c. Differential gene expression analysis within branch segment has also revealed the enrichment in Mesp1KO cells for Anxa2, Hand1 and Krt8. Since Hand1 is known to be expressed in extraembryonic tissue and juxtacardiac field, it would be important to validate the misexpression of Hand1 in Mesp1KO embryos. With the phenotype of Mesp1KO where Smarc3-F6 transgene is found expressed in the posterior side of the embryo, is it possible that the tissue found in the anterior part of Mesp1KO embryo is composed of extraembryonic-like mesoderm?

6. ScATAC-seq is really novel and important in this study. The authors have used scATAC-seq between E7.5 and E7.75 and compared control and Mesp1KO cells.
a. As for scRNA-seq and for more clarity, it would be important to precise the number of Mesp1KO and control cells analyzed.
b. The authors performed trajectory analysis and concluded that 'Mesp1KO cells traversed the normal path from epiblast to LPM and failed to abruptly progress toward CPs and CM'. What about other cardiac and non-cardiac smarcd3-F6 lineages? And how to assess that the path is normal since there is no control trajectory from epiblast to LPM with control cells (which should happen at earlier stages in control embryos).
c. Is there a link between transcription factors found as Mesp1-independent/resilient to Mesp1 loss or as Mesp1-dependent/ vulnerable to secondary effects of Mesp1 loss and Mesp1 binding as investigated in vitro by ChipSeq (Lin et al. 2022)?

Minor comment:
- A typo in line 218: Fgf5 is written twice
- In line 354: Is it Hand2 or Hand1? Hand2 is not mentioned in any other part of the manuscript or figures.
- There might be some confusion in Figure calling. Specifically: in line 177 E6-6.5 is figure 2F and in line 181 late E7.5 is also 2F.
- Why is Sox17 added in the model (Fig7) while not really investigated in detail in the manuscript?

Reviewer 3

Advance summary and potential significance to field

This paper provides a large sc-RNAseq and sc-ATACseq dataset looking at cardiac progenitor specification between E6.0 - E7.75, in the context of Mesp1 knockout mutant model. Phenotypic analysis of Mesp1 KO embryos revealed disrupted heart development and the accumulation of Smarcd3-F6 positive cells in the posterior of the developing embryo. By applying different profiling approaches, the authors describe and compare the transcriptional profiles of cardiac progenitors in Wt. and Mesp1 KO embryos and provide fluorescence in situ hybridization images in support of the transcriptional differences analysed. Finally, the authors go on to describe a disrupted regulatory landscape in mesoderm/cardiac progenitors when Mesp1 is deleted by profiling chromatin accessibility. The authors suggest that Mesp1 plays a different role in mesoderm specification than previously reported, acting at a later stage of development to prevent the expression of Nkx2.5 and differentiation towards cardiomyocytes.

© 2023. Published by The Company of Biologists under the terms of the Creative Commons Attribution License (https://creativecommons.org/licenses/by/4.0/).

The potential significance of this work relates to the characterisation of Smarcd3-F6+ positive cells being retained in the posterior of the embryo following Mesp1 KO and the in vivo epigenetic profiling of Wt. and Mesp1 KO mesoderm. The alternative view regarding the role of Mesp1 during cardiac differentiation would be of potential significance to the field however in its current state this interpretation is not supported strongly enough to advance the field.

Comments for the author

Overall, I feel this manuscript, in its current form, lacks clarity and needs significant changes in order to adequately support the authors interpretations and enable meaningful insight into the biological context of these descriptions.
The conclusion that Mesp1 KO CPCs differentiate normally until a breakpoint is not strongly supported. From the analysis shown both at the clustering (Figure 2B-D, Figure 3) and trajectory (Figure 4a) levels, other interpretations regaridng differentiation status could be made. There is a significant underrepresentation of Mesp1 KO cells in cell types which in the embyro precede precardiac mesoderm, to me suggesting Mesp1-KO differentiation is defective effected. This is clearly highlighted in the trajectory analysis were there a very few cells in subset 40 and even the Mesp1-KO cells in subset 34 are localised to the more differentiated/mature aspect of the trajectory. In contrast it is more convincing to look at the HPC subsets and conclude that differentiation proceeds normally. I would therefore offer an alternative interpretation that these findings suggest that Mesp1-KO cells differentiate abnormally, generating CPCs which haven't undergone the normal differentiation trajectory. Could they potentially skip the normal mesodermal differntiation pathway straight to Precardiac mesoderm? Given the limited numbers of Mesp1-KO cells collected from early and middle stages, it is also difficult to interpret anything conclusive regarding the earlier stages of mesoderm differentiation prior to Late E7.5 (late stage) in Mesp1-KO cells.

The clarity and continuity of the manuscript needs substantially improving it is very difficult to follow the manuscript and get a sense of what the authors are referring to in relation to the embryo. Cluster labelling varies depending on the analysis conducted making it difficult to understand how different cell types compare. Could the data be integrated into one clustering nomenclature or overlaid onto the WT clustering so that cell type identities remain constant throughout the manuscript. E.g., How does cluster 22 in the trajectory analysis relate to previous clustering as it is "not representative of any particular wildtype cell type", it would still be of interest to understand what these cells are, do they reflect an abnormal differentiation trajectory? What is the transcriptional profile of these cells, I presume they wouldn't have previously been examined as they are Smarcd3-F6 negative? Cell integration in the ATAC data is confusing when related to previous clustering, Figure 5C shows clusters 13 (Mesp1ME) and 9 (LPM2) are solely/predominantly derived from Mesp1 KO cells however previous analysis shows that clusters with the same name have either a mix of KO and WT cells (Figure 3G – Mesp1ME) or are all WT (Figure 2C – LPM2). How do these annotations relate to previous cell type descriptions?
What do cluster identities represent in the embryo, this is especially relevant given the authors suggest this is a resource paper. E.g., What is ExE Muscle and how does it relate to ExE, ExEMeso, LPM-ExEMeso and AntExe. What does a cardiac progenitor represent compared to a cardiac precursor. Obviously clustering and naming is a subjective approach, but it is very difficult to comprehend the cluster annotations provided and also trust that the marker genes relied on accurately allow such specific cell type annotation. Some cluster identities have not even been provided. In the Wt atlas do primordial germ cells represent a larger proportion of cells then LPM, previous references suggest PGCs represent ~100-150 cells in the embryo at E8.5 (Kim et al. 2011)?
How does this work fit into the scope of previously defined cardiac progenitor populations. For example, Zhang et al. 2021 report a broad Hand1 positive extraembryonic population of cardiac progenitors, whilst Tyser et al 2021. report a more restricted population of cardiac progenitors at the boundary of the LPM-ExE. How does this data fit with these previous observations, especially given 2B and C show that there are Mesp1-KO LPM-ExE cells and Figure 4K shows a massive upregulation of Anxa2 in the extraembryonic portion. How does the role of Mesp1 vary in the embryonic and extraembryonic portions of the embryo.

The rationale provided for certain analysis is lacking or not supported with detailed quantification. E.g., figure 3 selects certain clusters to perform differential analysis, why were these clusters selected. Line 222 states "we focused on the Smarcd3-F6 enriched Mesp1+ Mesendoderm cluster

© 2023. Published by The Company of Biologists under the terms of the Creative Commons Attribution License (https://creativecommons.org/licenses/by/4.0/).

(Mesp1ME) and its developmental predecessors, LSMeso2 cells.” By eye it looks like Smarcd3-F6 is enriched in the LPM-ExE cluster. Quantification detailing these kinds of claims would support the rationale for certain analysis approaches or maybe it would be more informative to examine all Smarcd3 positive clusters (or at least clusters containing a certain percentage and level of Smarcd3 expressing cells). What is the rationale for stating LSMeso 2 are Mesp1ME’s developmental predecessors, why aren’t LSMeso1 or LPM Mesp1ME’s developmental predecessors?
From the images provided it looks as if Smarcd3-F6 expression is reduced in Mesp1-KO embryos? Would this not limit the conclusions drawn/the analysis that can be conducted when assessing this population of cells. What differences would be detected, and interpretations concluded if an unbiased approach applied, not focusing solely on Smarcd3 cell types and their “potential” developmental predecessors.

Do all Mesp1-KO embryos show the same phenotype, the images of Smarcd3-F6 expression show varying expression profiles? Quantification should be provided in terms of the number of embryos examined and phenotypes observed. The Mesp1-KO phenotype could be better characterised, especially in relation to the posterior clump of Smarcd3-F6 GFP cells. Images in Figure1B are too small for the reader to identify the expression domains described.
The methods cite Ajima 2021 and Saga 1999 regarding the Mesp1 mouse model used. Do the authors use any of the new mice models generated in Ajima 2021 or do they solely use the Saga 1999 model with the PGK-Neo cassette? In this regard what is the expression levels of Mesp1 and Mesp2 in the dataset?

What does the following abstract sentence refer to “Mesp1 mutant CPCs failed robustly activate markers of cardiomyocyte maturity and critical cardiac TFs”, in figure 3E even the control Smarcd3 cells have low expression of Nkx2.5 and there aren’t other markers of CM maturity shown. And whilst I agree the trajectory analysis in figure 4 shows that Mesp1-KO cells have reduced Nkx2.5 it and in figure 4C the data shows that Mesp1-KO cells are predominantly giving rise to another cell fate (c22) not described in the manuscript.

First revision

<u>Author response to reviewers' comments</u>

Response to Reviewers
Krup et al, Development resubmission

We thank the reviewers for their helpful comments, which we have fully addressed as outlined below. All suggestions have greatly helped improve the manuscript.

Review #1

Specific Comments:

1.It’s remarkable how distinct the chromatin accessibility landscape is between control and Mesp1 KO cells (Fig. 5B & S11B). Notably absent from the motif analyses is Mesp1 itself. Was the Mesp1 motif enriched among sites that change between control and mutant?

Response: The Mesp1 motif is not among the highest-ranking motifs changed between control and mutant comparisons. We added lines 408-410 within the manuscript text to summarize these results. We included the complete list of motif rankings within differentially accessible peaks for control and mutant comparisons in Supplemental Table 7. Of note, the MESP1 motif does appear in our newly added analysis of sites directly bound by MESP1 (see next comment).

2.Related to question 1, the authors cite a recent paper (Lin et al. 2022) that describes in vitro induction of Mesp1 generates GATA4 accessible sites. Do the authors believe MESP1 acts as a pioneer-like factor to make GATA4 target sites accessible or is GATA4 providing that role? For example, in the list of sites that are not accessible in the mutant compared to the control with GATA4 motifs, do these sites also contain MESP1 motifs? Using the pseudotime of accessibility, are

© 2023. Published by The Company of Biologists under the terms of the Creative Commons Attribution License (https://creativecommons.org/licenses/by/4.0/).

there any clues about order of TF binding/accessibility gains with respect to these sites perhaps using footprinting analysis?

Response: Similar to the reviewers' cited results, we provide a summary referencing the conclusions from Lin and Swedlund et al, 2022 in lines 352-355. We observed Gata4 motif enrichment in cells from both genotypes (Fig. 5H), and we observed accessibility of Gata4-bound regions in EomesME, Mesp1ME, and LPM2 mutant cells, such as the self-regulated Gata4-G2 enhancer site (Fig. 6H). As suggested by the reviewers, we performed footprinting analysis within our scATACseq data and found the results inconclusive. We performed an additional comparative analysis with MESP1 ChIP-seq data from Lin and Swedlund et al, 2022 and differentially accessible scATAC-seq peak regions between Mesp1 mutant and control of our study, and included these analyses in a new Fig. 7 where we noted the presence of GATA motifs within both MESP1¬-dependent and MESP1-independent peak regions (Fig. 7B,D, Fig. S18B,D,F). Within the context of our Mesp1 mutant study we do not find definitive evidence to offer further interpretation on the dependency between MESP1 and GATA4 target site accessibility. Targeted mechanistic experiments beyond the scope of this study's question would be required to evaluate Mesp1-Gata4 dependency, and we envision that this study's in vivo generated datasets would complement these future investigations.

3.In the model described, there exists a breakpoint in an early cardiac progenitor. Weak expression of GATA4 and absence of NKX2-5 prevents these cells from progressing to mature cardiomyocytes in Mesp1 mutants. Based on the results described, such as ectopic localization of transgene expressing cells and gain of cardiac markers in other cell types in the mutants, these cells appear to be determined in their cell fate. Using the scATAC-seq, e.g., the sites that are still accessible in the mutant, and the earlier stage scRNA-seq, are there any clues that may suggest what may be acting upstream or in parallel of Mesp1 to determine cardiac fate? Given the continued expression of the Mesp1-indenepdent Eomes mechanism described, could Eomes be part of the cardiac cell fate determination process?

Response: We share the reviewer's interpretation that our results highlight the role of Eomes as an inducing transcriptional regulator for aspects of cardiac specification and thus part of the cardiac fate determination process at early stages. We describe this concept in lines 360-371, 550-553. In addition to the results of this work, we cite studies describing EOMES' role as both an early mesoderm and early cardiac transcriptional regulator inducing genes such as Mesp1 and Myl7 (Tosic et al, 2019; Costello et al, 2011; Alexanian et al, 2017; Guo et al, 2018; Probst et al, 2020). However, Eomes appears to be insufficient for maturation and full-differentiation of cardiac-fated cells, and the requirement for Mesp1 during mesoderm migration highlighted in Dominguez et al, 2022, which was also present in dysregulation of multiple migration-associated genes in this work, illustrates a need for both transcription factors to ensure proper differentiation of the cardiac lineage. Furthermore, when we compared MESP1 ChIP-seq data from Lin and Swedlund et al, 2022 with our scATAC-seq peaks, we noted a MESP1-bound peak region upstream of Eomes (Fig. 7J, red asterisk) within a candidate enhancer we predicted in our scATAC-seq peak2gene analysis in both mutant and control cells (Fig. 5I, Fig. 7J double asterisks). This MESP1-bound region coupled with our observations of upregulated and enduring Eomes expression in Mesp1 KO embryos (Fig. 2I, 2J) suggests a feedback relationship between Mesp1 and Eomes during early cardiogenesis.

Reviewer #2

General Comments:

It is sometimes unclear in the manuscript (and figures) which dataset is analyzed (all cells, curated for dual transgene expression or Smarcd3-F6+ only) and what are the differences obtained from analyzing Smarcd3-F6+ and dual transgene expression.

Response: We updated titles and descriptions of Ai14, Smarcd3-F6 double positive cells in relevant scRNA-seq figures to differentiate the cardiac-containing mesoderm subset of our data (double positive cells, Fig. 3, Fig. 4) from the CPC-only subset analyses (Smarcd3-F6+ cells, Fig. 2) and clarified in the text (line 193). We describe our rationale for pursuing analysis of the double positive cells (lines 191-193, Fig. 3) to understand disruptions of the cardiac-containing mesoderm beyond Smarcd3-F6+ cells, although our conclusions from data presented in Fig. 3 were similar to

© 2023. Published by The Company of Biologists under the terms of the Creative Commons Attribution License (https://creativecommons.org/licenses/by/4.0/).

the analyses we performed on Smarcd3-F6+ CPCs only (Fig. 2). We summarized this similarity between the analyses in lines 252-256.

Similarly, adding stages, axes and legends in pictures of embryos would help the reader to better appreciate the results.

Response: We have amended all figures with embryo images to contain axes denoting anatomy and embryonic ages.

The scATAC-seq in vivo is a real asset for this study, specifically to show chromatin regulation of early mesoderm, cardiac and non-cardiac genes in Mesp1KO or control cells. Since Mesp1-Chip seq data in vitro is now available (Lin et al. 2022) crossing the ATAC-seq data with Mesp1 peaks could be important to differentiate Mesp1-direct and Mesp1-indirect effects.

Response: We thank the reviewers for this constructive comment and have included new analyses comparing the Lin and Swedlund et al, 2022 MESP1 ChIP-seq dataset with our scATAC-seq data, detailed in Fig. 7, Fig. S18, and associated results sections lines 494-526. We found both MESP1-dependent and MESP1-independent differentially accessible peaks between control and Mesp1 KO cells.

This study has focused on trajectories from epiblast cells toward the cardiomyocyte lineages but could extend, without further experiments, on other cardiac lineages (endothelial) as well as other lineages including the extraembryonic lineages which has been recently linked to cardiac development (Tyser et al. 2021, Wang et al. 2022).

Response: Given the density of data presented in the manuscript we felt the focus on the cardiomyocyte lineage offered greater narrative clarity. We concur on the utility of this data for expanded studies and hope that our datasets presented here can be a resource for the field to support future studies of other cardiac and non-cardiac mesodermal lineages.

Specific Comments:

1.For clarification and easy reading of the manuscript, I would suggest to clarify the label of the UMAP representations in the different figures. For example, it is not clear whether the subsetted cell clusters in Fig1D corresponds to all mesoderm clusters or specifically to Ai14 and eGFP + clusters. Adding the label (Smarcd3-F6+) as in Fig 2B will help the reader to understand that the data have been subsetted based on eGFP expression.

Response: We have amended Fig. 1D to denote dual transgene subsetting, and we describe in further detail in Methods under analysis section “Wildtype Atlas”, lines 916-931.

2.The first part of the results describes the scRNAseq data. The authors nicely describe in subsetted clusters (Fig 1D) that the Smarcd3-F6+ transgene is expressed in clusters related to early cardiac development (mesoderm exiting the PS, anterior mesoderm, lateral plate mesoderm and cardiomyocytes)
a.The authors do not comment on the expression of Smarcd3-F6 in non-cardiac clusters annotated as the allantois, extraembryonic mesoderm, posterior lateral plate mesoderm and paraxial mesoderm. This is only described later when analyzing Mesp1KO embryos (see lines 135-146), which could be a bit confusing. It would probably be easier for the reader to describe all Smarcd3-F6+ clusters in the first paragraph along with the description of cardiac related clusters.

Response: We thank the reviewers for this suggestion, and have amended the text to clarify the cell types detected by Smarcd3-F6 within the initial description of the WT atlas in lines 107-113 and adjusted lines 137-140 to avoid redundancies.

b.In addition, it is not clear whether the 4,868 Smarcd3-F6+ cells (line 135) refer to control or Mesp1KO cells.

Response: We amended the text in what is now lines 128-129, and 130 to clarify that the 4,868 Smarcd3-F6+ cells dataset represents both control and Mesp1 KO cells.

© 2023. Published by The Company of Biologists under the terms of the Creative Commons Attribution License (https://creativecommons.org/licenses/by/4.0/).

c.A WT vs. KO Mesp1/Smarcd3-F6 lineage time course (from E6-E7.5) would be really interesting so we could visualize exactly what populations are being subsetted for all the bioinfo analyses? Maybe an addition to Fig. S1 or another panel with KO embryos at similar stages?

Response: We have added Fig. S4 with representative images of endogenous transgene fluorescence in Mesp1 KO embryos and littermate controls. Additional representations of Smarcd3-F6+ cells in Mesp1 KO and control embryos as detected by fluorescent RNA in situ are also present in Fig. 2, Fig. 4, and Fig. 5.

3.When comparing scRNAseq data from Mesp1KO and control cells, the authors nicely show that Mesp1KO cells are not represented in every cell type. a.In FigS4D, it might be better representing the data in term of relative proportion (to the total number of cells per genotype) rather than absolute number of cells. From the figure it seems that there are less Mesp1KO than control cells.

Response: We thank the authors for this suggestion and have amended Fig. S4D to represent relative proportion of cells per genotype in each scRNA-seq cluster. Due to challenges of acquiring stage-matched, littermate controls at exact timepoints from a mutant embryo with 25% Mendelian ratios, we were unable to acquire equal numbers of Mesp1 KO and control cells. In our Methods section line 959 we note the Smarcd3-F6+ Control vs. Mesp1 KO dataset contains 4,276 control cells and 592 Mesp1 KO cells.

b.It seems that while some clusters are not completely lacking Mesp1KO cells (allantois, posterior LPM, endothelium), the proportion is decreased. On the other side some clusters showed a higher proportion of Mesp1KO cells (pre-cardiac mesoderm, LPM, extraembryonic mesoderm). Can the authors comment and extend further their analysis on this point?

Response: We agree that the genotypes are disproportionately represented in cell types of both the Smarcd3-F6+ and double positive larger cardiac mesoderm control vs. Mesp1 KO atlases. We provide comment on this observation in lines 150-162 wherein we highlight that clusters such as precardiac mesoderm with a higher proportion of and presence of later embryo stage Mesp1 KO cells suggests that mutant cells get “stuck” in this stage of cardiac lineage differentiation, and the more differentiated cardiomyocyte cell types are comprised of later embryo stage control cells, wherein the cardiac lineage has progressed further.

4.Can Mesp2 compensate for the loss of Mesp1 and explain the transcriptional resilience and vulnerability seen in Mesp1KO cells? Knowing the redundance between Mesp1 and Mesp2, it would be important to show its expression. For the reader, it seems important to specify whether Mesp1Cre/Cre embryos show a decreased expression of Mesp2 and to which level. Is there remaining Mesp2 expression in some clusters?

Response: We queried the possibility of Mesp2 compensation within our Mesp1 KO cells and saw no differential expression (up or down) of Mesp2 relative to control in any of our scRNA-seq cell type genotype comparisons. We include the full results lists from all differential gene expression testing in Supplemental Tables 3-4. Thus, we concluded that Mesp2 is not behaving in a manner indicative of mutant related compensation or affect in our mouse model. Our results here are additionally explained by the recent Ajima et al, 2021 paper wherein the authors demonstrate that the pgk-neo cassette of the Cre insertion at the Mesp1 allele prevents compensation by Mesp2. The pgk-neo containing allele is the mouse we utilize here, and thus the Mesp1Cre/Cre genetic disruption represents an isolated knockout event for Mesp1. We clarify the mouse model we use in lines 803-804 of the Methods section.

5.The authors have used URD for pseudotemporal trajectory ordering. The analysis is very interesting and relevant. It nicely shows the branches that lacks Mesp1KO cells.
a.On the other hand, the authors underline that Mesp1KO cells are also found in its own pseudotime branch. This is really relevant to the study and could be further investigated. What\is the fate of these cells? Is it a transition state? Could it correspond to cells found in the anterior part of the embryo in Mesp1KO embryos?

© 2023. Published by The Company of Biologists under the terms of the Creative Commons Attribution License (https://creativecommons.org/licenses/by/4.0/).

Response: We agree this mutant deviation in the developmental progression is intriguing. Indeed, the results from the URD analysis assisted in us narrowing the developmental window for the subsequent experiments and analyses that ultimately led to our model of a developmental breakpoint where the mutant cells fail to differentiate further towards cardiac fate maturity. We were unable to definitively characterize the identity of these C22 cells from their gene expression profiles, noting more clearly in amended lines 270-271 that these cells are "defined by multiple mesodermal genes not representative of any previously characterized cardiac mesoderm cell type". The full gene lists are available in Tables S4B, S5. In addition to the differentially expressed genes highlighted in Fig. 4D-H, Table S5, in Fig. S10C we show that while cells of this C22 fate branch express Tnnt2, the expression is less enriched than the control cell-dominated CM and CardiacMeso fate branches, and the C22-fated cells express cardiac patterning and maturation genes to a lesser extent or hardly at all relative to control cells but do express mesoderm genes such as Tdgf1 and Snai1 that are also expressed in earlier mutant and control cell-containing pseudotemporal fate braches of the trajectory. When we consider these results in conjunction with our previous results where 1) The Smarcd3-F6+ pre-cardiac mesoderm cell cluster contained early and middle stage control and mutant embryos, yet only late-stage mutant embryos but not late-stage controls (Fig. 2B-D) and 2) Mutant cells of the Late mesoderm (Ai14, Smarcd3-F6 double positive cells) showed a pattern of increasingly altered gene expression as development progresses (Fig. 3 and lines 251-252) and 3) The aberrant posterior localization of the Smarcd3-F6+ cells in Mesp1 KO embryos relative to controls, we concluded that these C22 cells did not diverge to a new fate but rather represent a scenario where the CPCs did not mature properly. We feel the immaturity of the mutant cardiac mesoderm cells is due in part to their failure to migrate into the appropriate anterior embryonic position, which we show in a companion piece, Dominguez et al, 2022. When CPCs should be coalescing at the anterior midline by E7.75, Mesp1 KO CPCs have not and are instead being exposed to inappropriate signaling events in the posterior embryo which likely contributes to their ectopic expression of other mesodermal genes not present in control CPCs. We validated co-expression domains for CPC gene Myl7 with Smarcd3-F6+ cells in Fig. 4I, and show ectopic expression of non-cardiac Anxa2 within Smarcd3-F6+ cells in mutant embryos but not control (Fig. 4K). We note that key cardiac transcription factors such as Nkx2-5 were not expressed in cardiac regions of mutant embryos (Fig. 4J), indicating that in addition to the Mesp1-dependent migratory defect, a disrupted genomic and transcriptional regulatory landscape was also contributing to the immaturity and ectopic expression profiles of mutant CPCs, which we evaluate in the subsequent scATAC-seq analyses.

b.Additionally, it is worth noting that the endothelial, allantois and CFmeso branching lack Mesp1KO cells. This could also be further discussed. Since, these branches express Smarcd3-F6-eGFP and somehow Myl7 and Nkx2-5, do they correspond to the endothelium/endocardial lineages and part of the juxtacardiac field)?

Response: We evaluated expression of juxtacardiac field marker Mab21l2 in our URD trajectory and did not see any JCF-indicative enrichment of expression in the endothelial2, allantois, nor CFmeso branches, thus we do not interpret these branches as representative of the juxtacardiac field. We agree with the reviewer's interpretation that endothelial could correspond to an endocardial lineage, but we found that pursuit of this line of questioning would be beyond the scope of this current work on CPC specification and maturation without Mesp1. Fig. 4B to shows that these branches do also contain Mesp1 KO cells.

c.Differential gene expression analysis within branch segment has also revealed the enrichment in Mesp1KO cells for Anxa2, Hand1 and Krt8. Since Hand1 is known to be expressed in extraembryonic tissue and juxtacardiac field, it would be important to validate the misexpression of Hand1 in Mesp1KO embryos. With the phenotype of Mesp1KO where Smarc3-F6 transgene is found expressed in the posterior side of the embryo, is it possible that the tissue found in the anterior part of Mesp1KO embryo is composed of extraembryonic-like mesoderm?

Response: We thank the reviewers for this suggestion and have amended Fig. 4 to now include a new panel L where we show Hand1 expression domains corresponding with extraembryonic tissue proximal to the embryo proper in control and mutant embryos. Hand1 expression diffusely overlaps with posterior Smarcd3-F6+ CPCs in mutant embryos, however in littermate controls is most enriched in a restricted region proximal to Smarcd3-F6+ CPCs of the anterior cardiac crescent. We note a proximal anterior enrichment of Hand1 expression extending into the elongated anterior

© 2023. Published by The Company of Biologists under the terms of the Creative Commons Attribution License (https://creativecommons.org/licenses/by/4.0/).

ridge of the embryo proper, similar to the localization present anteriorly to the cardiac crescent in control embryos. These anterior expression domains in both genotypes are similar and reminiscent of patterns shown for the JCF in wildtype embryos in Tyser et al, 2021. Given that we see no evidence of migration from the extraembryonic mesoderm down into the anterior portion of the embryo in Mesp1 KO mutants in single cell tracking from live imaging in our related work (Dominguez et al, 2022) and an anterior enrichment of Hand1 is also present in control embryos, we do not have evidence to believe that the anterior portion of Mesp1 KO embryos is composed of extraembryonic-like mesoderm. We characterize an upregulation of Hand1 and Anxa2 in the same mutant cell populations (Fig. 4D), and while Anxa2 is expressed in the anterior extraembryonic regions of control embryos and in mutants, Anxa2 expression expands down into posterior CPC regions of the embryo proper in mutants (Fig. 4K) similarly to domains we see for Hand1 expression. We feel that these spatial validations for the upregulation of these genes in our scRNA-seq differential expression testing is in line with earlier observations (Fig. 2-3) of increasing dysregulation of gene expression in mutant CPCs as gastrulation progresses, which we further explore from the perspective of genomic landscape in subsequent scATAC-seq analyses.

6.ScATAC-seq is really novel and important in this study. The authors have used scATAC-seq between E7.5 and E7.75 and compared control and Mesp1KO cells.
a.As for scRNA-seq and for more clarity, it would be important to precise the number of Mesp1KO and control cells analyzed.

Response: We detail the number of Mesp1 KO and control cells analyzed by scATAC-seq in our Methods section line 1079 for the WTvsMut whole embryo dataset (46,819 cells; 26,295 control, 20,524 Mesp1 KO, Fig. S11). From this dataset we subsetted the WTvsMut mesoderm dataset presented in Fig. 5-7 which contained 25,848 cells; 14,212 control and 11,636 Mesp1 KO and is detailed in lines 1121-1122 of the Methods section.

b.The authors performed trajectory analysis and concluded that ‘Mesp1KO cells traversed the normal path from epiblast to LPM and failed to abruptly progress toward CPs and CM’. What about other cardiac and non-cardiac smarcd3-F6 lineages? And how to assess that the path is normal since there is no control trajectory from epiblast to LPM with control cells (which should happen at earlier stages in control embryos).

Response: Our focus within the scATAC-seq analyses described was informed by preceding scRNA-seq results highlighting increasingly dysregulated transcriptional signatures specifically within the cardiomyocyte lineage. Thus, with scATAC-seq we sought to assess CPC fate path potential towards more mature cardiomyocyte fates. While other trajectories can be queried from this data, we followed the line of investigation initially set up in the scRNA-seq URD analysis in an effort to keep our narrative and trajectory analyses focused. Given that scATAC-seq profiles of stage-matched, littermate Mesp1 KO embryo cells appear as developmentally “earlier” mesoderm cell type genomic landscapes relative to their control littermates, we did try to harvest additional younger control and mutant embryos which, consistent with our scRNA-seq characterization, would more developmentally similar in their genomic landscapes. Unfortunately, we found that these smaller embryos yielded insufficient numbers of nuclei that were prohibitive to the construction of a representative library. We commented on this need in our discussion lines 583-584 and added lines 1069-1073 in Methods to clarify this technical limitation within our study. With this caveat of our data in mind we focused our analysis to present a pseudotime path that was curated from what is expected based on the cardiac developmental literature and clarified this distinction in lines 334-343. We further noted in lines 341-342 that while cell clusters on the ‘starting’ side were comprised of mutant cells, cluster LPM1 contained both genotypes, and from our profiling in Fig. 5 and associated supplementals appears to have the most immature cardiac profile of mutant mesoderm cells in this dataset. We have included a control cell cardiac trajectory analysis traversing LPM1 to the CM/CP cell cluster and included these analyses in an additional Fig. S14, finding that the integrative analysis of gene expression and motif changes along this control trajectory appear comparable to the ending LPM1 to CM/CP transition noted in the mutant trajectory presented in Fig. 5F.

c.Is there a link between transcription factors found as Mesp1-independent/resilient to Mesp1 loss or as Mesp1-dependent/ vulnerable to secondary effects of Mesp1 loss and Mesp1 binding as investigated in vitro by ChipSeq (Lin et al. 2022)?

© 2023. Published by The Company of Biologists under the terms of the Creative Commons Attribution License (https://creativecommons.org/licenses/by/4.0/).

Response: We thank the reviewers for suggesting this analysis and have included a new Fig. 7 and Fig. S18 examining MESP1 binding peaks from the in vitro ChIP-seq data in Lin and Swedlund et al, 2022 with our differentially accessible regions by in vivo scATAC-seq comparing mutant and control cell types. We found that motifs of transcription factors such as NKX2-5, homeobox, TEAD, T-box, and GATA factors present in MESP1 independent peaks, indicating that the absent or decreased expression of factors Nkx2-5, Gata4 in Mesp1 KO cells is likely an indirect result of Mesp1 loss, and future studies will be needed to explore these mechanisms. We found evidence for EOMES/T-box motifs in both MESP1-dependent and -independent peak regions, indicating that while aspects of EOMES' participation in cardiac specification (such as activating Myl7 expression) can happen without Mesp1 as we observed and is shown by Tosic et al, 2019, additional feedback or shared regulatory activities require Mesp1 expression and transcription factor activity. We found a MESP1-binding peak within a candidate enhancer sequence we characterized from our scATAC-seq upstream of Eomes and with modestly increased accessibility in mutant C12-Mesp1ME, C13-Mesp1ME and LPM2, and control C14-CM/CP cell types. We speculate this enhancer region could represent a site for repressive MESP1 activity, however future work is needed to ascertain this possibility.

Minor comment:
-A typo in line 218: Fgf5 is written twice

Response: Thank you, duplication has been removed.

-In line 354: Is it Hand2 or Hand1? Hand2 is not mentioned in any other part of the manuscript or figures.

Response: Thank you for catching this typo; Hand1 would be the correct gene and has been corrected in the text.

-There might be some confusion in Figure calling. Specifically: in line 177
E6-6.5 is figure 2F and in line 181 late E7.5 is also 2F.

Response: These lines were meant to call Fig. 2F, G to highlight that from embryonic stages E6.5 (2F) to E7.5 (2G), Tdgf1 the expression is retained in mutants longer than control. To avoid confusion and improve clarity we have adjusted our in-text figure calls in now what are lines 171-172 to just call the later embryonic stage.

- Why is Sox17 added in the model (Fig7) while not really investigated in detail in the manuscript?

Response: We thank the reviewers for this feedback and agree that Sox17 has not been investigated in any real detail in this manuscript. To more accurately reflect the contents of what we did investigate, we have corrected the model (now Fig. 8) to exclude Sox17 because the only place we show Sox17 is as a differentially expressed gene in Fig. 2E.

Reviewer #3

General comments:

This paper provides a large sc-RNAseq and sc-ATACseq dataset looking at cardiac progenitor specification between E6.0 - E7.75, in the context of Mesp1 knockout mutant model. Phenotypic analysis of Mesp1 KO embryos revealed disrupted heart development and the accumulation of Smarcd3-F6 positive cells in the posterior of the developing embryo. By applying different profiling approaches, the authors describe and compare the transcriptional profiles of cardiac progenitors in Wt. and Mesp1 KO embryos and provide fluorescence in situ hybridization images in support of the transcriptional differences analysed. Finally, the authors go on to describe a disrupted regulatory landscape in mesoderm/cardiac progenitors when Mesp1 is deleted by profiling chromatin accessibility. The authors suggest that Mesp1 plays a different role in mesoderm specification than previously reported, acting at a later stage of development to prevent the expression of Nkx2.5 and differentiation towards cardiomyocytes. The potential significance of this work relates to the characterisation of Smarcd3-F6+ positive cells being retained in the posterior of the embryo following Mesp1 KO and the in vivo epigenetic profiling of Wt. and Mesp1 KO mesoderm. The

© 2023. Published by The Company of Biologists under the terms of the Creative Commons Attribution License (https://creativecommons.org/licenses/by/4.0/).

alternative view regarding the role of Mesp1 during cardiac differentiation would be of potential significance to the field however in its current state this interpretation is not supported strongly enough to advance the field.

Response: We thank the reviewer for this careful assessment related to previous literature. We believe the evidence presented and ultimate conclusion of our paper are slightly different from how this summary is presented in this reviewer comment, and are mostly consistent with the initial work of Saga et al, while different the conclusions reach by Lescroart et al. We aim to be careful in our summary of previous study results by acknowledging Mesp1's reported diverse roles during mesoderm specification in lines 65-73 of the introduction. We seek to align our study findings within the context of Mesp1's previously described roles in lines 543-564 of our discussion section. One main difference in our findings from previously reported conclusions in Lescroart et al, 2018 is that our data shows cardiac precursor cells exit pluripotency and embark incompletely and imperfectly towards cardiac fates despite lack of Mesp1, which we state in lines 560-564 of our discussion. We do not suggest a direct regulatory effect of MESP1 on Nkx2-5 at later stages of development as we did not find sufficient evidence in our results to support this claim. Rather, in lines 561-564 we interpret that our observations of failed Nkx2-5 induction and inappropriate expression for various Gata factors during the cardiogenic process signals a breakpoint where Mesp1 KO cells cannot progress further towards more mature cardiac fates. In lines 578-584 we acknowledge that because these failures appear in Mesp1 KO cells, we interpret the defects to be Mesp1-dependent despite occurring at developmental stages well after transient expression of Mesp1 has declined. We further speculate on possible mechanisms including improper repression of early regulators such as Eomes which we found to be upregulated in Mesp1 KO cells and lasting longer than in controls, other secondary effects influencing de-repression or ectopic activation of the noted non-cardiac mesodermal genes, and secondary effects caused by the improper anterior migration of Mesp1 KO CPCs, such as exposure to inappropriate signaling gradients. We do not believe we offer evidence or make claims of uncharacterized Mesp1 roles in this study. Rather, we feel we are presenting a detailed description of epigenomic and transcriptional changes during early cardiogenesis in the context of Mesp1 deficiency. Notably, our results complement findings from other works such as Saga et al, 1999 where the cardiac lineage is specified without Mesp1, with mutant embryos developing discernable, albeit unfused, heart tubes. Additionally, a recent work from Ajima et al, 2021 utilizes a new allele to highlight that Mesp1 is not required for heart specification. While the basis of the range of in vivo phenotypes for the original Mesp1 Cre allele from Saga et al, 1999 amongst labs is not known, the presence of the cardiac lineage in Mesp1 KO embryos has been previously observed.

Specific Comments:

Overall, I feel this manuscript, in its current form, lacks clarity and needs significant changes in order to adequately support the authors interpretations and enable meaningful insight into the biological context of these descriptions. The conclusion that Mesp1 KO CPCs differentiate normally until a breakpoint is not strongly supported. From the analysis shown both at the clustering (Figure 2B-D, Figure 3) and trajectory (Figure 4a) levels, other interpretations regaridng differentiation status could be made.

Response: We share the reviewers caution and do not claim differentiation is normal at early stages in Mesp1 KO embryos. We show differential gene expression between genotypes in our scRNA-seq analyses at early (e6.0-E6.5), middle (E6.5-E7.5) and late (E7.5-E7.75) stages of our assayed timeline in Figures 2-4 to highlight the increasingly disrupted transcriptomic profiles of Mesp1 KO cells.

We acknowledge that differences are present in early embryos in lines 145-149*: "Dividing the "all cells" genotype analysis into relative developmental stages to separate "Early" embryos (E6.0-E6.5), "Middle" embryos (late E6.5-E7.5), and "Late" embryos (late E7.5 to early E7.75), revealed that genotype discrepancies in cardiac-related gene expression are minor at Early stages and increasingly divergent with embryonic age (Fig. 2E)."

In lines 156-162* we highlight how both heterogeneity and transcriptomic severity increase as the embryo develops: "Many of the control-only cell types are comprised of Late-stage embryo cells (Fig. 2B-D, Fig. S5C), indicating that cell type heterogeneity is affected with loss of Mesp1 in

© 2023. Published by The Company of Biologists under the terms of the Creative Commons Attribution License (https://creativecommons.org/licenses/by/4.0/).

Smarcd3-F6+ cells with increasing severity as development progresses. Furthermore, while the preCardiacMeso and LSMeso cell types are represented by both genotypes in Early- and Middle-staged embryos, the Late-stage embryo cells represented in the preCardiacMeso are exclusively Mesp1 KO (Fig. 2B-D, Fig. S5C), indicating retention of precursor transcriptional profiles."

In Fig. S5B-E and Table S3 we show gene expression differences at various developmental stages between genotypes, including early stage embryos.

*These lines have been edited without changing their meaning to minimize words used in order to comply with resubmission word limits

There is a significant underrepresentation of Mesp1 KO cells in cell types which in the embyro precede precardiac mesoderm, to me suggesting Mesp1-KO differentiation is defective effected. This is clearly highlighted in the trajectory analysis were there a very few cells in subset 40 and even the Mesp1-KO cells in subset 34 are localised to the more differentiated/mature aspect of the trajectory. In contrast it is more convincing to look at the HPC subsets and conclude that differentiation proceeds normally.

Response: We share the reviewer's interpretation that Mesp1 KO cells are underrepresented in certain cell types, and feel this conclusion is supported by the data presented in Fig. 2-4 and summarized in some of the lines we noted above commenting on decreased heterogeneity and increasing phenotype severity with developmental progress. We were cautious to keep our interpretations of the various fate branches in the URD trajectory of Fig. 4 within the bounds of what URD pseudotemporal analysis specifically measures. While we do comment on the relatively earlier pseudotime positions of Mesp1 KO cells in CM and CardiacMeso fate branches in lines 266-269, our subsequent analyses and conclusions are agnostic to vertical position within a single branch. URD trajectory analysis allows us to compare genotypes within a fate branch or compare different segments at comparable pseudotime fate branches but since URD is designed to evaluate developmental branch points, we feel limited in our ability to derive biologically meaningful conclusions regarding differentiation status from comparisons of cell heights along a single fate branch. Thus we kept our differential gene expression testing between genotypes within the same segment or between branching segments at a comparable pseudotemporal branch point level.

I would therefore offer an alternative interpretation that these findings suggest that Mesp1-KO cells differentiate abnormally, generating CPCs which haven't undergone the normal differentiation trajectory.

Response: In lines 295-309 we summarize our findings of the results section "Mesp1 knockout cardiac mesoderm cells progress incompletely and imperfectly towards cardiomyocyte fates" in agreement with the reviewer's presented alternative interpretation that Mesp1 KO CPCs differentiate abnormally.

Could they potentially skip the normal mesodermal differntiation pathway straight to Precardiac mesoderm?

Response: We did not find compelling evidence for Mesp1 KO cells skipping any steps of differentiation. Indeed, we characterized mutant CPCs within precardiac mesoderm clusters (Fig. 2) and further evaluation of our "Middle" and "Late" developmental stage embryos in the scRNA-seq mesoderm dataset (Fig. 3) reveals that mutant embryos contains cells representative of epiblast, mesendoderm, and lateral plate mesoderm subtypes. These cell types are consistent with cardiac mesoderm lineage differentiation prior to cardiomyocyte progenitors. As mutant cells were not present in any of the more mature cardiac lineage cell types such as cardiomyocytes, we interpreted these results to indicate Mesp1 KO CPCs fail to progress further in differentiation rather than skipping differentiation steps.

Given the limited numbers of Mesp1-KO cells collected from early and middle stages, it is also difficult to interpret anything conclusive regarding the earlier stages of mesoderm differentiation prior to Late E7.5 (late stage) in Mesp1-KO cells.

© 2023. Published by The Company of Biologists under the terms of the Creative Commons Attribution License (https://creativecommons.org/licenses/by/4.0/).

Response: We also note that we were unable to make conclusions about genotype-related cell type population abundance from our scRNA-seq data owing to unequal numbers of Mesp1 KO and control cells in our scRNA-seq dataset. We acknowledge this technical limitation that we were unable to overcome through generation of additional Mesp1 KO embryos to our scRNA-seq dataset within the bounds of reasonable expense and timeframe in our Methods section in lines 881-884. For this limitation we relied on the orthogonal method of fluorescent RNA in-situ hybridization to validate key differentially expressed genes and examine their domains of expression and colocalization with the Smarcd3-F6 CPC marker within embryos, such as in Fig. 2F-K, Fig. 4I,K,L, and Fig. 5I.

The clarity and continuity of the manuscript needs substantially improving it is very difficult to follow the manuscript and get a sense of what the authors are referring to in relation to the embryo. Cluster labelling varies depending on the analysis conducted making it difficult to understand how different cell types compare.

Response: We thank the reviewer for this feedback and have amended embryo images to include anatomic axes and ages. We share a consolidated key of all abbreviations used to denote cell types in Table S1, and make all gene lists used for cluster annotation available in the supplementary tables associated with each figure's datasets. While each dataset's figure legends and Methods sub-section denotes how gene lists were generated for cluster annotations separately for each dataset, we have added a "Cell Type Annotations" subsection to Methods in lines 1026-1035 to clarify the dataset-relative annotating of clusters in a way that will be easier for readers to find. We hope the datasets generated through this work will be useful as resources for future studies, and have sought to make our data annotation as transparent as possible.

Could the data be integrated into one clustering nomenclature or overlaid onto the WT clustering so that cell type identities remain constant throughout the manuscript. E.g., How does cluster 22 in the trajectory analysis relate to previous clustering as it is "not representative of any particular wildtype cell type", it would still be of interest to understand what these cells are, do they reflect an abnormal differentiation trajectory? What is the transcriptional profile of these cells, I presume they wouldn't have previously been examined as they are Smarcd3-F6 negative?

Response: Regrettably, we have found that while consolidation of high-dimensional whole embryo data into a single dataset is useful to atlas initial broad categories of cell types represented, as we did with our scRNA-seq in Fig. 1 and Fig. S7, we risk 'over-smoothing' the very transcriptional nuances we generated the dataset to investigate. In our datasets we represent whole gastrulating embryos containing multiple cell types and differentiating lineages of progenitor cells. The analyses we presented are not easily interpreted when performed on a large singular dataset because biologically important differences would be masked by the decreased data resolution. Whole embryo datasets were generated because microdissection of specific lineages is not possible from morphology in early-stage gastrulation embryos, so we leveraged cell atlasing with our bioinformatic sorting of lineage reporter transgenes to subset data relevant to the specific developmental and genotype-related phenotypes we set out to investigate. The cardiac-lineage-specific analyses we performed on the various data subsets presented from the full WTvsMut dataset were informed by increasingly focused questions derived through the chain of hypothesis-generating analyses presented here in our narrative. We hope the WT and WTvsMut datasets generated through this work will be useful as resources for future studies, and for this desire have sought to make our data annotation as transparent as possible by including all gene lists available as supplemental tables.

We show that C22 contains Smarcd3-F6+ cells and comes from WTvsMut mesoderm dataset (Fig. S6E-H). We show top genes expressed in C22 and the other genes of the WTvsMut mesoderm dataset in Fig. S7I, and share the full gene lists in Supplemental Table S4B. C22 in Fig S7E is the same C22 used to curate the fate tip in the URD analyses of Fig 4, which we summarize in lines 261-264 defining URD roots and tips. To not overly burden the density of the main paper, we offer a more detailed explanation in Methods subsection "Single cell transcriptomic cell trajectories and pseudotime analysis" lines 1037-1061, and have specifically clarified the parent dataset utilized in line 1039. We apologize for misleading readers with an unclear summary of how this cluster C22 is defined by multiple mesodermal genes and have amended (now) lines 269-271 to now read "defined by multiple mesodermal genes not representative of any previously characterized cardiac mesoderm cell type".

© 2023. Published by The Company of Biologists under the terms of the Creative Commons Attribution License (https://creativecommons.org/licenses/by/4.0/).

What do cluster identities represent in the embryo, this is especially relevant given the authors suggest this is a resource paper. E.g., What is ExE Muscle and how does it relate to ExE, ExEMeso, LPM-ExEMeso and AntExe. What does a cardiac progenitor represent compared to a cardiac precursor. Obviously clustering and naming is a subjective approach, but it is very difficult to comprehend the cluster annotations provided and also trust that the marker genes relied on accurately allow such specific cell type annotation. Some cluster identities have not even been provided. In the Wt atlas do primordial germ cells represent a larger proportion of cells then LPM, previous references suggest PGCs represent ~100-150 cells in the embryo at E8.5 (Kim et al. 2011)?

Response: We appreciate the reviewers pointing out this oversight. While we based the original annotation largely on the enrichment of Ifitm1, as well as Crabp1 and Aldh1a2, we recognize our error in not considering the number of cells does not make sense for PGCs. We consulted the literature further and have amended this annotation to presomitic mesoderm. Whenever possible, we relied on consulting the literature, co-expression of known cell type markers, and transcription factor expression to annotate clusters as specific cell types. As the reviewers point out, clustering and naming can rely on the subjective best judgement of the interpreter, which is why we provide all our gene lists as supplemental tables. For our purposes in this paper, we were most interested in identifying clusters expressing our transgene reporters and subsetting for a more targeted cardiac and mesoderm analysis, which we were able to achieve from our data.

We utilize the term cardiac precursor for Smarcd3-F6+ cells to acknowledge the cardiac-restricted fate of these cells originally characterized in Devine et al, 2014. We deliberately use the term cardiac precursor/CPC instead of cardiac progenitor to avoid overstating the identity of these cells in deference to historic naming conventions wherein cells expressing Nkx2-5 were termed cardiac progenitors. Although the multiple more recent lineage tracing studies cited have expanded the definition of a cardiac progenitor to highlight that cardiac-restricted fates are prefigured in progenitor cells well prior to onset of Nkx2-5 expression (thus rendering this Nkx2-5 definition a potential semantic trap) we felt 'cardiac precursor' best represented the Smarcd3-F6+ cells while avoiding confusion related to Nkx2-5 expression.

How does this work fit into the scope of previously defined cardiac progenitor populations. For example, Zhang et al. 2021 report a broad Hand1 positive extraembryonic population of cardiac progenitors, whilst Tyser et al 2021. report a more restricted population of cardiac progenitors at the boundary of the LPM-ExE. How does this data fit with these previous observations, especially given 2B and C show that there are Mesp1-KO LPM-ExE cells and Figure 4K shows a massive upregulation of Anxa2 in the extraembryonic portion.

Response: We have added a new panel Fig. 4L to show enriched expression of Hand1 in the JCF in control embryos, and Hand1 expression domains diffusely overlapping with Smarcd3-F6+ regions in Mesp1 KO embryos. Our presentation of Anxa2 in Fig. 4K shows both upregulation in extraembryonic regions and co-expression in Smarcd3-F6+ cells of the posterior embryo proper in Mesp1 KO embryos, in contrast to the restricted anterior extraembryonic pattern seen in controls. The co-expression with Smarcd3-F6+ domains in the embryos validates the upregulation of Anxa2 and Hand1 in Mesp1 KO CM-fated cells relative to controls (Fig. 4D). Further lineage tracing experiments beyond the scope of this work would be needed to disentangle the mutant extraembryonic upregulation unique to Anxa2 but not Hand1.

How does the role of Mesp1 vary in the embryonic and extraembryonic portions of the embryo. The rationale provided for certain analysis is lacking or not supported with detailed quantification. E.g., figure 3 selects certain clusters to perform differential analysis, why were these clusters selected. Line 222 states "we focused on the Smarcd3-F6 enriched Mesp1+ Mesendoderm cluster (Mesp1ME) and its developmental predecessors, LSMeso2 cells." By eye it looks like Smarcd3-F6 is enriched in the LPM-ExE cluster. Quantification detailing these kinds of claims would support the rationale for certain analysis approaches or maybe it would be more informative to examine all Smarcd3 positive clusters (or at least clusters containing a certain percentage and level of Smarcd3 expressing cells). What is the rationale for stating LSMeso 2 are Mesp1ME's developmental predecessors, why aren't LSMeso1 or LPM Mesp1ME's developmental predecessors?

© 2023. Published by The Company of Biologists under the terms of the Creative Commons Attribution License (https://creativecommons.org/licenses/by/4.0/).

Response: This study risks being overly data-dense and complex, which all reviewers noted, and thus we were only able to present certain comparisons from our analyses in the figures and text. We opted to maintain our focus on the clear deficits observed in CPCs, although we offer additional comparisons such as the noted LPM-ExEM cells in Fig. S6 and Table S3. Our focus on CPCs relative to extraembryonic mesoderm was also supported by results from a companion live imaging work Dominguez et al, 2022 which did not illuminate extraembryonic compensation for the cardiac mesoderm in Mesp1 KO mutants.

From the images provided it looks as if Smarcd3-F6 expression is reduced in Mesp1-KO embryos?

Response: The reviewer is correct. We note this observation in lines 168-169: “Notably, Mesp1 KO embryos at early stages showed decreased or delayed expression of Smarcd3-F6”

Would this not limit the conclusions drawn/the analysis that can be conducted when assessing this population of cells. What differences would be detected, and interpretations concluded if an unbiased approach applied, not focusing solely on Smarcd3 cell types and their “potential” developmental predecessors.

Response: We shared this awareness for potentially biasing ourselves and sought to address this possibility by conducting the broader mesoderm analysis shown in Fig. 3. We amended lines 190-192 to better clarify this rationale: “Following characterization of Mesp1 KO effects in Smarcd3-F6+ cells specifically, we investigated potential alterations to the broader mesoderm, inclusive of Smarcd3-F6+ cells and the cardiac mesoderm marked by Ai14.”

Do all Mesp1-KO embryos show the same phenotype, the images of Smarcd3-F6 expression show varying expression profiles? Quantification should be provided in terms of the number of embryos examined and phenotypes observed.

Response: The varying expression domains and levels for Smarcd3-F6 is also present in control embryos and is related to the stages (earlier vs later) of the embryos as the cardiac lineage develops. Representative images of endogenous fluorescent reporters are provided for both control and Mesp1 KO embryos in Fig. 1B, Fig. S1, Fig. S10, and a new Fig. S#. Fluorescent RNA in situ hybridization is used in our studies to validate scRNA-seq results and qualitative expression domains relative to Smarcd3-F6+ cells. Quantitative statistics are made available in all relevant differential testing figures and supplemental tables for scRNA-seq and scATAC-seq data. Embryo replicate details are provided in the Methods sections for each experimental paradigm and in lines 881-884, 1076-1078, and in the GEO dataset.

The Mesp1-KO phenotype could be better characterised, especially in relation to the posterior clump of Smarcd3-F6 GFP cells. Images in Figure1B are too small for the reader to identify the expression domains described.

Response: We have enlarged the images in Fig. 1B for the WT embryos as much as bounds of figure can reasonably allow, and have made additional larger representative images of endogenous fluorescent reporters available for both control and Mesp1 KO embryos in Fig. 1B, Fig. S1, Fig. S11, and a new Fig. S4. Phenotypes are consistent and demonstrated in representative validations of expression domains for multiple transcripts, including Smarcd3-F6 via eGFP, by fluorescent RNA in situ in Fig. 2A, 2F-K, Fig. 4I-L, Fig. 5I, and by immunofluorescence in Fig. S9B. We utilize an enhancer transgene to validate the Tdgf1 differentially accessible enhancer region in Fig. 6K.

The methods cite Ajima 2021 and Saga 1999 regarding the Mesp1 mouse model used.
Do the authors use any of the new mice models generated in Ajima 2021 or do they solely use the Saga 1999 model with the PGK-Neo cassette?

Response: We use the Saga, et al 1999 mouse model with the pgk-neo cassette. This has been clarified in the methods section.

In this regard what is the expression levels of Mesp1 and Mesp2 in the dataset?

© 2023. Published by The Company of Biologists under the terms of the Creative Commons Attribution License (https://creativecommons.org/licenses/by/4.0/).

Response: We queried the possibility of Mesp2 compensation within our Mesp1 KO cells and saw no differential expression (up or down) of Mesp2 relative to control in any of our scRNA-seq cell type genotype comparisons. We include the full results lists from all differential gene expression testing in Supplemental Tables 3-4. Thus, we concluded that Mesp2 is not behaving in a manner indicative of mutant related compensation or affect in our mouse model.

What does the following abstract sentence refer to “Mesp1 mutant CPCs failed robustly activate markers of cardiomyocyte maturity and critical cardiac TFs”, in figure 3E even the control Smarcd3 cells have low expression of Nkx2.5 and there aren’t other markers of CM maturity shown. And whilst I agree the trajectory analysis in figure 4 shows that Mesp1-KO cells have reduced Nkx2.5 it and in figure 4C the data shows that Mesp1-KO cells are predominantly giving rise to another cell fate (c22) not described in the manuscript.

Response: The noted abstract lines are intended to succinctly summarize the diminished or lacking expression of cardiomyocyte-associated muscle structure genes such as Tnnt2, Actc1, Ttn, Myl7, and others and the diminished or lacking expression of transcription factors with demonstrated roles in cardiac development, particularly during organogenesis phases when heart fields are defining anatomy, such as Nkx2-5, Mef2c, Hand2, Tbx5, Gata4/5/6, and others. We show these results in more detail across multiple figures and tables.

The low expression of Nkx2-5 in control embryos is because we are assaying relatively early developmental time points during cardiogenesis. Devine et al, 2014 shows that Smarcd3-F6 is activated prior to a number of cardiac TFs such as Nkx2-5, Mef2c, Tbx5. As previously noted, onset of robust Nkx2-5 expression is occurring in the later and cardiac crescent stages of the timepoints we assayed.

Similar to our responses above and to Reviewer #2’s comment 5a, while we were unable to definitively name these C22 cells from their gene expression profiles, we show that C22 contains Smarcd3-F6+ cells and share the full gene lists in Supplemental Table S4B. In Fig. S10C we show that while cells of this C22 fate branch express Tnnt2, the expression is less enriched than the control cell-dominated CM and CardiacMeso fate branches, and the C22-fated cells express cardiac patterning and maturation genes to a lesser extent or hardly at all relative to control cells but do express mesoderm genes such as Tdgf1 and Snai1 that are also expressed in earlier mutant and control cell-containing pseudotemporal fate branches of the trajectory. When we consider these results in conjunction with our previous results where 1) The Smarcd3-F6+ pre-cardiac mesoderm cell cluster contained early and middle stage control and mutant embryos, yet only late-stage mutant embryos but not late-stage controls (Fig. 2B-D) and 2) Mutant cells of the Late mesoderm (Ai14, Smarcd3-F6 double positive cells) showed a pattern of increasingly altered gene expression as development progresses (Fig. 3 and lines 251-252) and 3) The aberrant posterior localization of the Smarcd3-F6+ cells in Mesp1 KO embryos relative to controls, we concluded that these C22 cells did not diverge to a new fate but rather represent a scenario where the CPCs did not mature properly.

Second decision letter

MS ID#: DEVELOP/2022/201229

MS TITLE: A Mesp1-dependent developmental breakpoint in transcriptional and epigenomic specification of early cardiac precursors

AUTHORS: Alexis Leigh Krup, Sarah Winchester, Sanjeev Ranade, Ayushi Agrawal, W. Patrick Devine, Tanvi Sinha, Krishna Choudhary, Martin Dominguez, Reuben Thomas, Brian L Black, Deepak Srivastava, and Benoit Bruneau

I have now received all the referees' reports on the above manuscript, and have reached a decision. The referees' comments are appended below, or you can access them online: please go to BenchPressand click on the 'Manuscripts with Decisions' queue in the Author Area.

© 2023. Published by The Company of Biologists under the terms of the Creative Commons Attribution License (https://creativecommons.org/licenses/by/4.0/).

As you will see, the referees express considerable interest in your work, but have some significant criticisms and recommend a further revision of your manuscript before we can consider publication. If you are able to revise the manuscript along the lines suggested, I will be happy receive a revised version of the manuscript. As you will see two of the reviewers raise some technical concerns, and one suggests that you discuss alternative interpretations of your findings as to whether loss of Mesp1 results in differentiation stalling along the normal pathway (i.e. a "breakpoint") or whether cells are re-directed on an aberrant differentiation trajectory before failing.

Your revised paper will be re-reviewed by one or more of the original referees, and acceptance of your manuscript will depend on your addressing satisfactorily the reviewers' major concerns. Please also note that Development will normally permit only one round of major revision. If it would be helpful, you are welcome to contact us to discuss your revision in greater detail. Please send us a point-by-point response indicating your plans for addressing the referee’s comments, and we will look over this and provide further guidance.

Please attend to all of the reviewers' comments and ensure that you clearly highlight all changes made in the revised manuscript. Please avoid using 'Tracked changes' in Word files as these are lost in PDF conversion. I should be grateful if you would also provide a point-by-point response detailing how you have dealt with the points raised by the reviewers in the 'Response to Reviewers' box. If you do not agree with any of their criticisms or suggestions please explain clearly why this is so.

Reviewer 1

Advance summary and potential significance to field

all my concerns were addressed this is an important paper

Comments for the author

all my concerns were addressed

Reviewer 2

Advance summary and potential significance to field

In their manuscript, Leigh Krup et al. seek to understand cardiac precursor cell (CPC) specification during gastrulation and the role of the transcription factor Mesp1 in this process. They have used single-cell transcriptomics and single-cell chromatin accessibility in control and Mesp1KO mouse embryos. They show that Mesp1KO cells initiate the early steps of cardiac specification but are then blocked in a state when Eomes is highly expressed and cannot differentiate into the cardiomyocyte lineages. These results are complementary to previous analysis of Mesp1KO cells in vivo but the previous analysis was performed at early stages of development. Finally, the main novelty of the paper is the single cell chromatin accessibility analysis in both control and KO cells performed in vivo for the first time at these early stages with comparison with published in vitro MESP1-Chip-seq data. It has thus revealed some Mesp1-dependent and Mesp1-independent regulation of key cardiac genes in vivo and describe very interesting regulatory regions that are progressively activated along cardiac specification.

Comments for the author

Leigh Krup and colleagues have submitted a revised manuscript addressing some of the concerns exposed in our previous review. This includes clarifications on the data analyzed and comparison with MESP1-Chip-seq dataset, allowing the identification of MESP1-dependent and MESP1-independent differentially accessible peaks between control and Mesp1 KO cells.

The authors have thus added a number of analyses that have significantly improved their manuscript.

© 2023. Published by The Company of Biologists under the terms of the Creative Commons Attribution License (https://creativecommons.org/licenses/by/4.0/).

However, in its current form, we still have few concerns as of how the conclusions of the manuscripts are supported by the data presented. It could be fixed by discussing or understating some of the conclusions.

1. Our first concern relates to the stages of embryos collected for the comparison between controls and Mesp1KO and the strong conclusions on the contrasting results from previous reports.
The authors have now included, in a new Fig. S4, representative images of “early”, “middle” and “late” control and Mesp1-KO embryos. This led us to realize that embryos defined as “early” showed landmarks of mid-gastrulation stages. According to the stages from Downs and Davies 1993 “early” control and Mesp1-KO embryos likely correspond to Middle to Late Streak stages (E7.0-E7.5) since the length of the primitive streak (as judged by the extension of Mesp1 lineage) was between 51 and 100%. It thus raises some concerns on the stages of “early” Mesp1-KO embryos used for scRNAseq. Furthermore, while the cell number in the embryo at gastrulation range from 660 at E6.5 to approximately 15,000 at E7.5 (Lawson et al. 1991; Tzouanacou et al. 2009), it is surprising that about 1000 Mesp1KO mesodermal cells (Methods lines 857-868) could be collected at the earliest stages of gastrulation from a single embryo. In addition, from the methods (lines 823-835), it seems, from the annotations, that all embryos were not strictly age-matched (4 control samples annotated E6.0 and defined as Early streak or Mid-streak in FigS1 1 control annotated E6.5 defined as Late streak/Early Bud in FigS1 and the Mesp1-KO embryo annotated E6.5). We are thus wondering whether a bias could not have been introduced by comparing early controls with a mutant embryo at a slightly later stage. The definition of the stages in general would need to be revised for clarification as classification of gastrulation-stage embryos by Downs and Davies the reference used by the authors to stage the embryos, starts from E6.75.
This is important since the authors have concluded that their data are in contrast with previous reports where analysis with Mesp1-KO cells was performed at the early stage of gastrulation (E6.75). It does not change the analysis between control and Mesp1-KO embryos but it slightly changes the conclusions. We could thus alternatively interpret the data presented in the manuscript as indicative that Mesp1-KO cells finally exit pluripotency at mid-gastrulation and that the failure to exit pluripotency previously highlighted in early stages indicates a delay rather than a complete breakpoint.

2. The second point relates to interpretation and discussion of combined scRNAseq and multiplex in situ hybridization data. For Hand1 expression, scRNAseq data indicates that Hand1 is upregulated in CM fated cells of Mesp1-KO embryos. The new multiplex in situ hybridization (Fig 4L) nicely shows an upregulation of Hand1 in the juxta-cardiac field (?) and extraembryonic mesoderm but it seems that it is expressed at lower level in the Smarcd3-F6+ posterior domain. We feel that this point should be further discussed in the manuscript. Does this data really validate an upregulation of Hand1 in CM fated cells of Mesp1-KO embryos or is it finally cell type dependent? Doesn’t the data (expansion of Anxa2 and Hand1 into the posterior embryo proper) itself suggest a possible acquisition of extra-embryonic fate? It would need to be clarified also in the model proposed in Fig. 8.

Minor comments:
- Paragraph 224-239 is repeated in 240-255.
- Axes are still missing in Fig1B and 2A

<u>Reviewer 3</u>

Advance summary and potential significance to field

This paper provides a sc-RNAseq and sc-ATACseq dataset looking at cardiac progenitor specification between E6.0 - E7.75, in the context of a Mesp1 knockout mutant model. Phenotypic analysis of Mesp1 KO embryos revealed disrupted heart development and the accumulation of Smarcd3-F6 positive cells in the posterior of the developing embryo. By applying different profiling approaches, the authors describe and compare the transcriptional profiles of cardiac progenitors in wild type and Mesp1 KO embryos and provide fluorescence in situ hybridization images to highlight transcriptional differences analysed. Finally, the authors go on to describe a disrupted regulatory landscape in mesoderm/cardiac progenitors when Mesp1 is deleted by profiling chromatin accessibility. The authors show that Mesp1 KO leads to dysregulated mesoderm differentiation and suggest that cardiac prognitor formation is arrested at a specific breakpoint.

© 2023. Published by The Company of Biologists under the terms of the Creative Commons Attribution License (https://creativecommons.org/licenses/by/4.0/).

The potential significance of this work relates to the characterization of Smarcd3-F6+ positive cells being retained in the posterior of the embryo following Mesp1 KO and the in vivo epigenetic profiling of Wild type and Mesp1 KO mesoderm. This is very much a descriptive study, characterizing a previously described mutant and, as the authors state in their response, the findings are “mostly consistent with the initial work of Saga et al”. Whilst this uses recent single cell sequencing approaches to further characterize the model, it does not provide sufficient functional assays to provide significant mechanistic insight.

Comments for the author

I acknowledge the slight modifications and oversight corrections, such as the misannotation of presomitic mesoderm as PGCs, however the data presented still does not conclusively support the conclusions made and the response does not sufficiently address my previous comments and in fact glosses over biologically-relevant points as well as information regarding reporting/reproducibility. Given the descriptive nature of the article, it may be more suited as a resource article although I feel it still would require further experiments and the conclusions regarding a breakpoint to be moved to a discussion point. How does the data support that a breakpoint occurs at E7.5-E7.5 (discussion line 578), when mesoderm patterning is severely disrupted at E6.0/E6.5 (Figure 2), the scRNASeq dataset contains barely any Mesp1-KO cells at the corresponding and fundamental timepoint (Early/Mid; as acknowledged by the author in their response) and there are minimal embryos used for validation. In summary I do not feel this manuscript is suitable for publication, as the data presented is insufficient to support the conclusions made and no experimentally tested mechanistic insight is provided. Below I have reiterated some of my previous limitations with this study which haven’t been sufficiently addressed:

1. Insufficient number of cells characterized. Mesp1-KO cells from early and mid-stage Mesp1-KO embryos need to be increased, especially given the phenotypes shown at E6.0 and E6.5. Whilst the authors state this is a technical limitation, the data is fundamental to the biological characterization and to support the conclusions proposed. How can you make comparisons with a cell type that is not/minimally present (Figure 2C-E; also see point below on data reporting). Does the lack of Mesp1-KO cells represent “technical” under-sampling at early stages or is there a significant reduction in precardiac mesoderm cell types at this early stage of development? This is also true for the pseudo time analysis in Figure 4, given the authors feel limited in their ability to derive biologically meaningful conclusions regarding differentiation status, why is this analysis conducted, comparisons at the clustering level should be sufficient. Why do the authors propose that there are no Mesp-1 KO cells in the early cardiac mesoderm segments? From the images provided it could be suggested that this is biological phenotype.

2. Phenotype characterization is not sufficient. This is especially relevant given the limitations of the single-cell data presented and the variability in embryos shown in Figure 2. Detailed and quantified phenotypic characterization is required to understand the scRNA-seq data presented and also provide more insight into the mechanistic cause of this phenotype. For example assessment at the cellular level of tissue morphology, cell death or cell cycle in a time-resolved manner would aid in understanding/characterizing the phenotype.

3. Data reporting, reproducibility and quantification. As previously requested, I have been unable to find any details regarding the number of embryos/litters used for in situ analysis and high-resolution phenotypic analysis. The authors in their response highlight that the embryo replicate details are provided in the Methods sections for each experimental paradigm and in lines 881-884, 1076-1078, and in the GEO dataset, however this only relates to the sequencing experiments. Do the images in Figure 2I & 2K show the same embryo, it also looks like the same embryos is used in Figure 4I and 4K? How many times were these experiments repeated? I do not understand the response stating that the variability is due to “earlier vs later” stages of embryo development. This highlights exactly why describing/quantifying the phenotype in more embryos is required, so that embryos of “equivalent” age can be compared, in a time resolved manner. Details regarding n numbers for all experiments should be a provided and imaging experiments conducted on multiple embryos from different litters. Data quantification and validation for statistical approaches is fundamental, claiming this would make the article “overly data-dense and complex” does not address this point, maybe some of the redundant analysis could be removed. Single-cell

© 2023. Published by The Company of Biologists under the terms of the Creative Commons Attribution License (https://creativecommons.org/licenses/by/4.0/).

RNAseq datasets allow for unbiased statistical analysis. In figure 2E, what is the exact number of cells compared, is this analysis sufficiently powered, these kinds of details need to be reported.

4. No mechanistic insight. Whilst some bioinformatic results have been validated, there are no functional assays performed to experimentally test or provide novel mechanistic insight.

Second revision

Author response to reviewers' comments

Reviewer 1 Advance summary and potential significance to field
all my concerns were addressed this is an important paper

Reviewer 1 Comments for the author
all my concerns were addressed

Reviewer 2 Advance Summary and Potential Significance to Field: In their manuscript, Leigh Krup et al. seek to understand cardiac precursor cell (CPC) specification during gastrulation and the role of the transcription factor Mesp1 in this process. They have used single-cell transcriptomics and single-cell chromatin accessibility in control and Mesp1KO mouse embryos. They show that Mesp1KO cells initiate the early steps of cardiac specification but are then blocked in a state when Eomes is highly expressed and cannot differentiate into the cardiomyocyte lineages. These results are complementary to previous analysis of Mesp1KO cells in vivo but the previous analysis was performed at early stages of development. Finally, the main novelty of the paper is the single cell chromatin accessibility analysis in both control and KO cells performed in vivo for the first time at these early stages with comparison with published in vitro MESP1-Chip-seq data. It has thus revealed some Mesp1-dependent and Mesp1- independent regulation of key cardiac genes in vivo and describe very interesting regulatory regions that are progressively activated along cardiac specification.

Reviewer 2 Comments for the Author:
Leigh Krup and colleagues have submitted a revised manuscript addressing some of the concerns exposed in our previous review. This includes clarifications on the data analyzed and comparison with MESP1-Chip-seq dataset, allowing the identification of MESP1-dependent and MESP1-independent differentially accessible peaks between control and Mesp1 KO cells.

The authors have thus added a number of analyses that have significantly improved their manuscript.

However, in its current form, we still have few concerns as of how the conclusions of the manuscripts are supported by the data presented. It could be fixed by discussing or understating some of the conclusions.

1. Our first concern relates to the stages of embryos collected for the comparison between controls and Mesp1KO and the strong conclusions on the contrasting results from previous reports. The authors have now included, in a new Fig. S4, representative images of "early", "middle" and "late" control and Mesp1-KO embryos. This led us to realize that embryos defined as "early" showed landmarks of mid-gastrulation stages. According to the stages from Downs and Davies 1993 "early" control and Mesp1-KO embryos likely correspond to Middle to Late Streak stages (E7.0- E7.5) since the length of the primitive streak (as judged by the extension of Mesp1 lineage) was between 51 and 100%. It thus raises some concerns on the stages of "early" Mesp1-KO embryos used for scRNAseq. Furthermore, while the cell number in the embryo at gastrulation range from 660 at E6.5 to approximately 15,000 at E7.5 (Lawson et al. 1991; Tzouanacou et al. 2009), it is surprising that about 1000 Mesp1KO mesodermal cells (Methods lines 857- 868) could be collected at the earliest stages of gastrulation from a single embryo. In addition, from the methods (lines 823-835), it seems, from the annotations, that all embryos were not strictly age-matched (4 control samples annotated E6.0 and defined as Early streak or Mid-streak in FigS1, 1 control annotated E6.5 defined as Late streak/Early Bud in FigS1 and the

© 2023. Published by The Company of Biologists under the terms of the Creative Commons Attribution License (https://creativecommons.org/licenses/by/4.0/).

Mesp1-KO embryo annotated E6.5). We are thus wondering whether a bias could not have been introduced by comparing early controls with a mutant embryo at a slightly later stage. The definition of the stages in general would need to be revised for clarification as classification of gastrulation-stage embryos by Downs and Davies, the reference used by the authors to stage the embryos, starts from E6.75. This is important since the authors have concluded that their data are in contrast with previous reports where analysis with Mesp1-KO cells was performed at the early stage of gastrulation (E6.75). It does not change the analysis between control and Mesp1-KO embryos but it slightly changes the conclusions. We could thus alternatively interpret the data presented in the manuscript as indicative that Mesp1-KO cells finally exit pluripotency at mid-gastrulation and that the failure to exit pluripotency previously highlighted in early stages indicates a delay rather than a complete breakpoint.

Response:
The Mendelian ratio for recovery of *Mesp1* KO embryos is 25%, which in practice required dissections of hundreds of embryo litters to 1) capture mutants, 2) capture litters with stage-matched genotypes, especially at early stages of gastrulation when embryonic ages vary significantly along the uterine horn, and 3) capture the appropriate time point needed for the genomics time courses and/or *in situ* validations. These variables coupled with the increasingly divergent morphology of *Mesp1* KO embryos as development progresses presented a challenging scenario we recognized as a limitation. The acquisition of these embryos required diligent effort across years, including through significant pandemic-related shutdowns and disruptions to animal husbandry and laboratory experiments. We clearly acknowledge and provide descriptions and images of all embryos and replicates in our methods, and sought to overcome any potential limitations of our data by carefully replicating and validating our interpretations by orthogonal experimental methods such as *in situ* and scATAC-seq before presenting conclusions. Additionally, we made use of trajectory inference analyses in both the scRNA-seq and scATAC-seq analyses of this study to rectify biological order of single cell fates in an unbiased manner orthogonal to experimenter-assigned staging and to overcome any potential time gaps that might be present in our time course. We provide descriptions of embryos and genotypes used in construction of our *Mesp1* KO genomics datasets with names matching nomenclature in deposited GEO data in Methods Lines 820-832, Lines 868-872, 971-975, 991-994. Additional clarifications on replicates and multiplexed embryo assays have been added into methods in lines 1134-1140, 1151.

We do not find evidence to substantiate the possibility of a delayed failure to exit pluripotency in *Mesp1* KO CPCs. Our conclusions in this work that mutant CPCs are specified towards cardiac fates upon exit from the primitive streak similar to controls until a breakdown when organogenesis should be occurring is further corroborated by our other recent paper (Dominguez et al, 2023). In Dominguez et al, 2023 we show that *Mesp1*+ cells exit the streak at the same time as control cells, are capable of migratory behaviors, however lack the critical anterior-lateral direction in their movements that is prerequisite to convergence at the anterior midline for formation of the cardiac crescent.
Thus, the developmental breakpoint in mutant CPC progression towards cardiac fates is supported by transcriptional, epigenomic, imaging, and live cell tracking data both in this work and Dominguez et al, 2023. The consensus offered across all modalities is that mutant CPCs are specified out of pluripotency and exit the primitive streak, begin both a transcriptional progression towards cardiac fates and movement anterior-laterally, yet their failure to proceed further in cardiac maturation is marked by de-repression of mesendoderm transcriptional programs, ectopic expression of non-cardiac mesodermal genes, failure to adequately express transcriptional regulators of organogenesis. This failure is further corroborated by observations of mutant migrating cardiac mesoderm cells losing organized directionality in live cell tracking of pre-crescent embryos. We have clarified these distinctions in the text lines 278-280, 535-544.

2. The second point relates to interpretation and discussion of combined scRNAseq and multiplex in situ hybridization data. For Hand1 expression, scRNAseq data indicates that Hand1 is upregulated in CM fated cells of Mesp1-KO embryos. The new multiplex in situ hybridization (Fig 4L) nicely shows an upregulation of Hand1 in the juxta-cardiac field (?) and extraembryonic mesoderm but it seems that it is expressed at lower level in the Smarcd3-F6+ posterior domain. We feel that this point should be further discussed in the manuscript. Does this data really validate an upregulation of Hand1 in CM fated cells of Mesp1-KO embryos or is it finally cell type dependent? Doesn't the data (expansion of Anxa2 and Hand1 into the posterior embryo proper)

© 2023. Published by The Company of Biologists under the terms of the Creative Commons Attribution License (https://creativecommons.org/licenses/by/4.0/).

itself suggest a possible acquisition of extra- embryonic fate? It would need to be clarified also in the model proposed in Fig. 8.
Response:
While we sought to take care in reporting our interpretations of URD trajectory data, differentially expressed genes, and the *in situs* presented in Fig. 4, we have amended lines 278-303 to attempt to more directly address the complexity of these results. We do not find evidence to suggest acquisition of an extra-embryonic fate. The additional *in vivo* fate mapping required to definitively and experimentally differentiate extraembryonic from embryonic mesoderm fates would represent a study in its own right, and would require resources and time beyond the scope of the narrative currently presented in this manuscript. To further reconcile the complexity of possible interpretations for *Hand1* expression in *Mesp1* KO cardiac mesoderm cells despite experimental limitations, we turned to recent live cell tracking of the cardiac mesoderm in *Mesp1* KO embryos (Dominguez et al, 2023) to highlight clear movement differences distinguishing *Mesp1* KO mesoderm cells of the embryo proper from observations in extraembryonic regions, compartmentalized boundaries between distinct cell populations, and further discuss cardiac-mesoderm gene expression changes in these cells that also ectopically express non-cardiac mesoderm gene markers.

Minor comments:
- *Paragraph 224-239 is repeated in 240-255.*

Response:
Thank you for noticing this formatting error, we have removed the accidentally duplicated section.
- *Axes are still missing in Fig1B and 2A*

Response:
Axes have been added.

Reviewer 3 Advance Summary and Potential Significance to Field:
This paper provides a sc-RNAseq and sc-ATACseq dataset looking at cardiac progenitor specification between E6.0 E7.75, in the context of a Mesp1 knockout mutant model. Phenotypic analysis of Mesp1 KO embryos revealed disrupted heart development and the accumulation of Smarcd3-F6 positive cells in the posterior of the developing embryo. By applying different profiling approaches, the authors describe and compare the transcriptional profiles of cardiac progenitors in wild type and Mesp1 KO embryos and provide fluorescence in situ hybridization images to highlight transcriptional differences analysed. Finally, the authors go on to describe a disrupted regulatory landscape in mesoderm/cardiac progenitors when Mesp1 is deleted by profiling chromatin accessibility. The authors show that Mesp1 KO leads to dysregulated mesoderm differentiation and suggest that cardiac prognitor formation is arrested at a specific breakpoint. The potential significance of this work relates to the characterization of Smarcd3-F6+ positive cells being retained in the posterior of the embryo following Mesp1 KO and the in vivo epigenetic profiling of Wild type and Mesp1 KO mesoderm. This is very much a descriptive study, characterizing a previously described mutant and, as the authors state in their response, the findings are “mostly consistent with the initial work of Saga et al”. Whilst this uses recent single cell sequencing approaches to further characterize the model, it does not provide sufficient functional assays to provide significant mechanistic insight.

Reviewer 3 Comments for the Author:
I acknowledge the slight modifications and oversight corrections, such as the misannotation of presomitic mesoderm as PGCs, however the data presented still does not conclusively support the conclusions made and the response does not sufficiently address my previous comments and in fact glosses over biologically-relevant points as well as information regarding reporting/reproducibility. Given the descriptive nature of the article, it may be more suited as a resource article although I feel it still would require further experiments and the conclusions regarding a breakpoint to be moved to a discussion point. How does the data support that a breakpoint occurs at E7.5-E7.5 (discussion line 578), when mesoderm patterning is severely disrupted at E6.0/E6.5 (Figure 2), the scRNASeq dataset contains barely any Mesp1-KO cells at the corresponding and fundamental timepoint (Early/Mid; as acknowledged by the author in their response) and there are minimal embryos used for validation. In summary I do not feel this

© 2023. Published by The Company of Biologists under the terms of the Creative Commons Attribution License (https://creativecommons.org/licenses/by/4.0/).

manuscript is suitable for publication, as the data presented is insufficient to support the conclusions made and no experimentally tested mechanistic insight is provided. Below I have reiterated some of my previous limitations with this study which haven't been sufficiently addressed:

1. Insufficient number of cells characterized. Mesp1-KO cells from early and mid-stage Mesp1-KO embryos need to be increased, especially given the phenotypes shown at E6.0 and E6.5. Whilst the authors state this is a technical limitation, the data is fundamental to the biological characterization and to support the conclusions proposed. How can you make comparisons with a cell type that is not/minimally present (Figure 2C-E; also see point below on data reporting). Does the lack of Mesp1-KO cells represent "technical" under-sampling at early stages or is there a significant reduction in precardiac mesoderm cell types at this early stage of development? This is also true for the pseudo time analysis in Figure 4, given the authors feel limited in their ability to derive biologically meaningful conclusions regarding differentiation status, why is this analysis conducted, comparisons at the clustering level should be sufficient. Why do the authors propose that there are no Mesp-1 KO cells in the early cardiac mesoderm segments? From the images provided it could be suggested that this is biological phenotype.

Response:
The lack of Mesp1 KO cells is not a technical issue. We have made very clear all cell numbers for each comparisons characterized and make use of multiple orthogonal experimental methods and analyses with statistical significance reported within figures and supplemental tables for all interpretations and conclusions presented in our paper. We report statistical significance for all analyses and any conclusions made were supported by replicates, orthogonal approaches, and clear description of methods and reporting of data values. Additionally, we make use of different advanced trajectory inference analyses that query our data in ways agnostic of embryonic staging, with reported statistical significance for all comparisons made. We further utilize published datasets and conclusions and results from related studies, both *in vitro* and *in vivo,* to contextualize our findings succinctly within prior frameworks and understandings of cardiogenesis. The analysis in Fig. 4 is presented in an unbiased way in order to assess the fate potential of *Mesp1* KO cardiac mesoderm cells, and we conclude based on shared cardiac fate paths between both control and mutant genotypes that *Mesp1* KO cells progress incompletely and imperfectly, as shown by statistically significant, in-figure p-values and readily available supplemental data tables with all statistics shown for differential gene expression. We do not claim that there are no *Mesp1* KO cells in the early mesoderm segments; see the *Mesp1* KO blue dots in the cardiac fate tree following segment 34 downwards in Fig. 4A-B, descriptions in lines 250-252, 258-280, 281-284. These data analysis are revisited with orthogonal analyses using scATAC-seq data in Fig. 5 and Fig. 6, including additional trajectory analysis in Fig. 5F using scATAC-seq data. Trajectory inference is a sophisticated and unbiased way of assessing the developmental trajectories. In our case it has clearly shown that early mesoderm emergence is maintained in the absence of MESP1, but that later differentiation fails. This is then supported by the ATAC-seq data. We certainly do not feel limited in our ability to derive biologically meaningful conclusions, and in fact have come up with some very strong and clear conclusions.

2. Phenotype characterization is not sufficient. This is especially relevant given the limitations of the single-cell data presented and the variability in embryos shown in Figure 2. Detailed and quantified phenotypic characterization is required to understand the scRNA-seq data presented and also provide more insight into the mechanistic cause of this phenotype. For example assessment at the cellular level of tissue morphology, cell death or cell cycle in a time-resolved manner would aid in understanding/characterizing the phenotype.

Response:
We focused this study on a question of transcriptional regulation of cardiac specification and present high-resolution quantifications and descriptions of gene expression and chromatin accessibility single cells with lineage resolution during *in vivo* cardiac development. To help address the reviewer's concern, please see our related paper in which we have characterized cellular movement behaviors of the cardiac mesoderm *in vivo* (Dominguez et al 2023), and showed that the *Mesp1* KO cells are alive and well, divide normally, and migrate at the same rate as WT cells, albeit in a disorganized non-directional manner. We have clarified this in the text in lines 535-544.

© 2023. Published by The Company of Biologists under the terms of the Creative Commons Attribution License (https://creativecommons.org/licenses/by/4.0/).

3. Data reporting, reproducibility and quantification. As previously requested, I have been unable to find any details regarding the number of embryos/litters used for in situ analysis and high-resolution phenotypic analysis. The authors in their response highlight that the embryo replicate details are provided in the Methods sections for each experimental paradigm and in lines 881-884, 1076-1078, and in the GEO dataset, however this only relates to the sequencing experiments. Do the images in Figure 2I & 2K show the same embryo, it also looks like the same embryos is used in Figure 4I and 4K? How many times were these experiments repeated? I do not understand the response stating that the variability is due to "earlier vs later" stages of embryo development. This highlights exactly why describing/quantifying the phenotype in more embryos is required, so that embryos of "equivalent" age can be compared, in a time resolved manner. Details regarding n numbers for all experiments should be a provided and imaging experiments conducted on multiple embryos from different litters. Data quantification and validation for statistical approaches is fundamental, claiming this would make the article "overly data-dense and complex" does not address this point, maybe some of the redundant analysis could be removed. Single-cell RNAseq datasets allow for unbiased statistical analysis. In figure 2E, what is the exact number of cells compared, is this analysis sufficiently powered, these kinds of details need to be reported.

Response:
We provide replicate descriptions of embryos and genotypes used in construction of our *Mesp1* KO genomics datasets with names matching nomenclature in deposited GEO data in Methods Lines 820-832, Lines 868-872, 971- 975, 991-994. Additional clarifications on replicates and multiplexed embryo assays have been added into methods in lines 1134-1140, 1151. Descriptions of all statistical tests and associated parameters, exact functions and parameters for respective analyses performed, and careful descriptions of workflows are detailed in Methods. All data tables of all results from comparisons queried and described gene lists are reported in supplemental tables.

4. No mechanistic insight. Whilst some bioinformatic results have been validated, there are no functional assays performed to experimentally test or provide novel mechanistic insight.

Response:
Our intention in providing detailed analysis of chromatin accessibility with mutant and control embryos, correlated appropriate use of statistical analysis with differential gene expression, intersection with transcription factor chromatin occupancy datasets, *in situ* validation of both differentially expressed genes and distal regulatory elements *in vivo* in mutants and controls at relevant stages, and utilization of lineage markers within high resolution genomics datasets was to offer the maximal amount of insight possible into our overarching goal to describe very early cardiac specification. In this study we leveraged classical developmental biology, the physiological relevance of embryonic development *in vivo*, and high-resolution genomics and bioinformatics analyses. We took an Occam's Razor abductive approach to explain how early cardiac specification is governed by both *Mesp1*-dependent and -independent transcriptional events. We pursued a hypothesis requiring the fewest assumptions about master regulators (perdurance of *Smarcd3*-F6+ cells, demonstrated to be early cardiac precursors despite loss of an early TF expressed in the cardiomyocyte lineage, *Mesp1*), including analyses that would be agnostic to our predictions (trajectory inference analyses agnostic to curation by embryonic age), and multiple orthogonal approaches to offer a model for transcriptional events occurring during early cardiac specification.

This reviewer comment does not make clear which functional assays would need to be deployed to satisfy a request for further novel mechanistic insight. We posit that as described above we have provided an extensive and strong developmental genetics study that surpasses the thoroughness of many comparable published works. The manuscript has been further strengthened during the review process by our inclusion of analyses intersecting our *in vivo* scATAC-seq dataset with MESP1 chromatin occupancy, statistical measure of single cell correlations between gene expression and chromatin accessibility, and spatial and temporal validations of claims made within whole mount embryos. We find it further challenging to imagine what more could be accomplished within this story. We firmly believe that as currently presented, our body of work is complete and reaches solid and relevant conclusions with novel mechanistic insights distinguishing aspects of cardiac development previously uncharacterized in the field.

© 2023. Published by The Company of Biologists under the terms of the Creative Commons Attribution License (https://creativecommons.org/licenses/by/4.0/).

Third decision letter

MS ID#: DEVELOP/2022/201229

MS TITLE: A Mesp1-dependent developmental breakpoint in transcriptional and epigenomic specification of early cardiac precursors

AUTHORS: Alexis Leigh Krup, Sarah Winchester, Sanjeev Ranade, Ayushi Agrawal, W. Patrick Devine, Tanvi Sinha, Krishna Choudhary, Martin Dominguez, Reuben Thomas, Brian L Black, Deepak Srivastava, and Benoit Bruneau
ARTICLE TYPE: Research Article

Apologies for the delay in getting back to you - for some technical reason the ms got stuck in the BenchPress system for over a week and I only just discovered this error....

I am happy to tell you that your manuscript has been accepted for publication in Development, pending our standard ethics checks.

© 2023. Published by The Company of Biologists under the terms of the Creative Commons Attribution License (https://creativecommons.org/licenses/by/4.0/).
